# Strategic investment in tuberculosis control in the Republic of Bulgaria

**DOI:** 10.1017/S0950268819001857

**Published:** 2019-11-18

**Authors:** T. N. Doan, T. Varleva, M. Zamfirova, M. Tyufekchieva, A. Keshelava, K. Hristov, A. Yaneva, B. Gadzheva, S. Zhang, S. Irbe, R. Ragonnet, E. S. McBryde, J. M. Trauer

**Affiliations:** 1 Department of Medicine at The Royal Melbourne Hospital, University of Melbourne, Melbourne, Victoria, Australia; 2 Australian Institute of Tropical Health and Medicine, James Cook University, Townsville, Queensland, Australia; 3 Promotion and Prevention of Diseases and Addictions, Ministry of Health, Sofia, Bulgaria; 4 Programmes Management Unit, Ministry of Health, Sofia, Bulgaria; 5 The Global Fund to Fight AIDS, Tuberculosis and Malaria, Geneva, Switzerland; 6 The Burnet Institute, Melbourne, Victoria, Australia; 7 School of Public Health and Preventive Medicine, Monash University, Melbourne, Victoria, Australia

**Keywords:** End TB targets, mathematical modelling, transmission dynamics

## Abstract

As Bulgaria transitions away from Global Fund grant, robust estimates of the comparative impact of the various response strategies under consideration are needed to ensure sustained effectiveness of the tuberculosis (TB) programme. We tailored an established mathematical model for TB control to the epidemic in Bulgaria to project the likely outcomes of seven intervention scenarios. Under existing programmatic conditions projected forward, the country's targets for achieving TB elimination in the coming decades will not be achieved. No interventions under consideration were predicted to accelerate the baseline projected reduction in epidemiological indicators significantly. Discontinuation of the ‘Open Doors’ program and activities of non-governmental organisations would result in a marked exacerbation of the epidemic (increasing incidence in 2035 by 6–8% relative to baseline conditions projected forward). Changing to a short course regimen for multidrug-resistant TB (MDR-TB) would substantially decrease MDR-TB mortality (by 21.6% in 2035 relative to baseline conditions projected forward). Changing to ambulatory care for eligible patients would not affect TB burden but would be markedly cost-saving. In conclusion, Bulgaria faces important challenges in transitioning to a primarily domestically-financed TB programme. The country should consider maintaining currently effective programs and shifting towards ambulatory care to ensure program sustainability.

## Introduction

Although the World Health Organisation (WHO) European Region contributes only 3% to the global tuberculosis (TB) burden, 85% of TB cases in the Region arise in Eastern European and Central Asian countries [1]. With an incidence rate of 23 cases per 100 000 population in 2016, Bulgaria is one of 18 ‘high priority’ countries for TB control in the WHO European Region [2]. Multidrug-resistant TB (MDR-TB) remains a problem in the country and constituted 1.2% of new TB cases and 22.8% of re-treatment TB cases in 2016 [1]. Around 62% of all TB cases in Bulgaria are culture-confirmed, of which ~86% receive drug susceptibility testing (DST) [3].

The TB control program in Bulgaria is characterised by extensive involvement of non-governmental organisations (NGOs), which have been active since 2009. Their TB care activities include spatial mapping of high-risk groups, screening for active TB and latent TB infection (LTBI) in high-risk groups, awareness raising and providing support to patients under treatment. Since 2009, regular community campaigns have been carried out across the country to raise public awareness about TB, and to offer individuals with respiratory symptoms the opportunity to present themselves to public hospitals for free-of-charge TB diagnosis and treatment (the ‘Open Doors’ program).

Roma communities are among the most important TB risk groups in Bulgaria, accounting for around 5–10% of the total population, but contributing 30 and 50% respectively of all reported DS-TB and MDR-TB cases in the country [3]. Diabetes is also a major threat to TB control efforts in Bulgaria, with the prevalence of type II diabetes among adults in Bulgaria reaching 8.4% in 2015 [4, 5].

In line with the WHO Sustainable Development Goals (SDGs) and the End TB Strategy targets [6], Bulgaria aims to achieve TB elimination by 2050. Interim targets aim to reduce incidence and TB-related mortality by 40% in 2025, and by 90 and 95% respectively in 2035. However, a return to domestically-funded TB programs following the completion of the current Global Fund to Fight AIDS, Tuberculosis and Malaria (henceforward ‘the Global Fund’) grant, from which several key TB control activities in the country are financed, may jeopardise the program's sustainability. Specifically, the risk of discontinuation of NGO activities and the Open Doors program could worsen control. As Bulgaria transitions from external donor support, the country is considering approaches to strengthen the National Strategic Plan and improve the sustainability and efficiency of its TB program. To do so, the country needs reliable estimates of the epidemiological and financial impact of disease control interventions. Mathematical modelling provides a platform to quantify and compare the projected costs and epidemiological impact of different intervention scenarios to guide TB strategic policy [7]. We describe the application of the ‘AuTuMN’ software platform to TB control and planning in Bulgaria [7].

## Methods

### General approach

The AuTuMN software platform has been previously described [7]. The full details of the epidemiological model are described in the Supplementary Material and summarised as follows. As no individual patient data were used, ethical approval was not required.

Given the slow-moving and complex nature of the TB epidemic, we aimed for a high degree of consistency with historical TB dynamics in Bulgaria and permitted a high degree of model complexity (1199 compartments). To achieve this, we first automatically loaded publicly available data for birth, death, vaccination and TB-related programmatic measures and then fit functions to map parameter values to time for these quantities using polynomial spline functions (Supplementary Section 3).

### Demographic model

Individuals first enter the model with the time-variant birth rate fit to World Bank data (Supplementary Fig. S1), with the proportions of births vaccinated and unvaccinated split according to the WHO/UNICEF-reported vaccination coverage (Supplementary Fig. S2). Population-wide natural mortality was also parameterised to data from the World Bank (Supplementary Fig. S3) and applied to all compartments. All compartments were stratified by age, with age groups being: under 5 years, 5 to 15 years, 15 to 25 years and 25 years and above.

### Model of Mycobacterium tuberculosis transmission and progression

From the point of infection onwards, all compartments were also sub-divided by strain (i.e. DS-TB and MDR-TB). The force of infection was calculated as the effective number of infectious persons, which included those yet to present for care, missed (false-negative) presentations who had not yet re-started care seeking and those patients who were in very early stage treatment. All paediatric and smear-negative TB patients were considered to have markedly reduced infectiousness compared to adults [8–10]. BCG programs in Bulgaria have varied over the years with revaccination sometimes performed. However, in the absence of clear evidence for the effectiveness of this approach, we assume that BCG-vaccinated children retain partial immunity from infection until age 15 which wanes thereafter (Supplementary Section 8). Latent infection was considered to confer partial immunity, modifying the force of infection applied to fully susceptible persons; while the relative susceptibility/immunity attributable to a previously treated disease episode was considered highly uncertain and was estimated through the calibration process.

Following infection, the latent period to active disease was simulated using two sequential compartments, with infected persons initially entering a high-risk early latent state before transitioning to a lower risk late latent compartment [11, 12]. The proportion of infected persons progressing to active disease from early latency was greater for children than adults [11] and each new episode of TB was distinguished according to smear status into smear-positive, smear-negative pulmonary and extrapulmonary. Diabetic persons had an approximately threefold increased risk of progression to active TB following infection (Supplementary Section 14) [13]. An untreated episode of active TB was assumed to last for three years, with a greater case fatality rate for smear-positive than for smear-negative and extrapulmonary TB (Supplementary Section 9) [14].

### Population sub-groups

We incorporate three population risk groups of particular importance to the epidemiology of TB in Bulgaria: persons with diabetes mellitus, the prison population and Roma communities. Diabetes is implemented as affecting progression rates to disease following infection, whereas the other two groups are considered to mix assortatively and have higher contact rates to reflect poorer living conditions (Fig. S21). The proportion of the population with diabetes was applied to the oldest age group (25 and above) only (Supplementary Fig. S9); while the prison population was only simulated in age groups 15 years and above.

### Simulation of the health care system

The rate of TB detection by the health care system was calculated such that the proportion of cases detected was determined by the reported time-variant case detection rate. Treatment outcomes were divided into (1) success, (2) death and (3) unfavourable outcomes other than death. The probability of reaching each of these three outcomes was time-variant, parameterised according to WHO data and differed according to the patient's treatment history.

### Calibration

We calibrated the model by adjusting five important but uncertain epidemiological parameters to align with Bulgaria's reported TB incidence for the period 2010 to 2016 only, as the Bulgarian National Tuberculosis Program (NTP) and country's experts considered estimates prior to 2010 to be less reliable. Model calibration was further verified by comparing model predictions with WHO estimates regarding prevalence and reported mortality. Calibrated parameters were then used to project the future epidemiology under baseline conditions and different counterfactual scenarios.

### Interventions

In addition to the baseline conditions in which all time-variant parameters were maintained at their 2016 values, we developed a set of six programmatic interventions in collaboration with the country's NTP (Table 1). These interventions were designed to represent real-world programmatic changes currently under consideration by the Bulgarian NTP. The interventions were modelled by varying relevant model parameters from their baseline values, with the changes informed by reviews of the literature (undertaken by JMT, TND, RR and ESM). Two authors (JMT and TND) assessed the standard of evidence for the interventions implemented blinded to each other's assessments, with complete agreement between the two blinded assessments.

**Table 1.** Simulated interventions

Name and description of intervention	Level of evidence^a^	Applicability of evidence	Primary evidence source	Coverage achievable (by 2020)	Model implementation
1. Short course regimen for MDR-TB	2	Programmatic	Van Deun *et al*. [15] Piubello *et al*. [16] Kuaban *et al*. [17] Aung *et al*., [18]	50%	Treatment duration of 12 months and treatment success rate of 84% (range 84 to 90%).
2. Scale-up DST coverage in culture-positive TB cases	5	Clinical	Nil	100%	Increase proportion of DST in culture-positive cases from baseline value of 86% to 100%.
3. Scale-up food vouchers for all patients under treatment	1	Programmatic	Martins *et al*. [19] Lutge *et al*. [20] Ciobanu *et al*. [21]	100%	Decrease in all treatment outcomes other than success (i.e. death and non-death unfavourable outcomes) by 50% (range 40 to 60%).
4. Transition from inpatient care to ambulatory care for smear-negative and extrapulmonary DS-TB patients	2	Clinical	Bassili *et al*. [22] Fitzpatrick *et al*. [23] Horter *et al*. [24]	100%	No change to the epidemiological model. Treatment outcomes are assumed to be similar for ambulatory care as compared to inpatient care.
5. Discontinue Open Doors program	5	Programmatic	Bulgaria NTP	0%	Reduce case detection proportion by 8% (range 5 to 12%).
6. Discontinue NGO activities	5	Programmatic	Bulgaria NTP	0%	Reduce case detection proportion by 8% (range 5 to 12%) in the general population and by a further 25% (range 15 to 30%) in the Roma community.

DST, drug-susceptibility testing; MDR, multidrug-resistant; NGO, non-governmental organisation; NTP, National TB Program; TB, tuberculosis.

a Level of evidence was graded according to the Oxford Centre for Evidence-Based Medicine 2011 Levels of Evidence. Level 1 indicates strongest evidence, whereas level 5 indicates weakest evidence.

For the purpose of discussion below, we also present an unrealistic scenario which represents complete cessation of all transmission of *Mtb* from 2017 onwards with other parameters as for the baseline scenario.

### Economics

A logistic relationship was used to estimate the expected costs incurred from a given level of coverage of each intervention and the size of the population targeted by that intervention, as previously described [8]. Logistic functions can allow coverage to saturate at high-spending levels, thus better reflecting programmatic reality. Start-up costs for each new intervention simulated were applied separately and spread over the initial years for which that intervention was implemented. The economic inputs estimated in collaboration with the Bulgaria NTP are presented in Supplementary Table S2 and the cost-coverage curves are presented in Supplementary Figure S16.

## Results

### Calibration and predicted epidemic trajectory

**Fig. 1.**
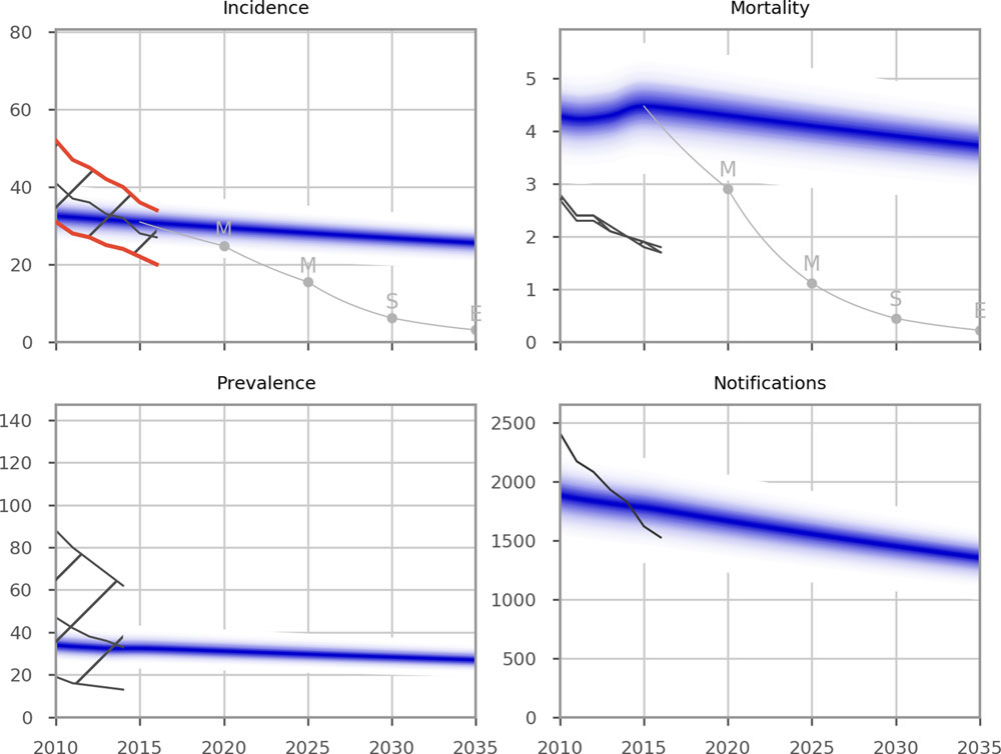
Epidemiological calibration results. The blue shaded areas represent model estimates. The black lines and hatched areas are mean values and confidence limits, respectively, for indicator data from the WHO Global TB report (2016 and 2017). Data from the WHO Global TB Report 2017 were used for the years from 2000 to 2016. As the WHO Global TB Report 2017 did not report data prior to 2000, the WHO Global TB Report 2016 was used for data points for the years that were not included in the 2017 report (1990 to 1999). The red lines in the Incidence panel indicate the WHO incidence data points against which the model was calibrated. Grey lines represent the SDG targets for 2030, the End TB Strategy targets for 2035 and the interim milestones for 2020 and 2025. Units of all horizontal axes are calendar years and units of vertical axes are: incidence, per 100 000 per year; mortality, per 100 000 per year; prevalence per 100 000; notifications, absolute number.

Figure 1 presents the epidemiological predictions obtained from the automatic calibration process described above. Although TB incidence was the only indicator targeted during calibration, the predicted values of prevalence and notifications also closely matched WHO's reported estimates for the recent years; while the model suggested that mortality may be under-reported, particularly in the most recent estimates. Despite the remarkably close calibration to multiple indicators without any attempt to target them, the evolution of the TB epidemic over time was less closely matched, with the rate of decline in burden over recent years not fully captured through the incorporation of time-variant programmatic parameter fit to country-specific data alone. Given that Bulgaria is a low-transmission setting, it may be that the model's slow decline in disease burden is a closer reflection of reality than the more variable reported programmatic estimates or that non-programmatic factors (e.g. improvements in general living conditions) are also playing a role. The model predicted that none of the targets set for incidence and mortality in 2025 and 2035 would be achieved under current programmatic conditions.

### Predicted intervention effectiveness

**Fig. 2.**
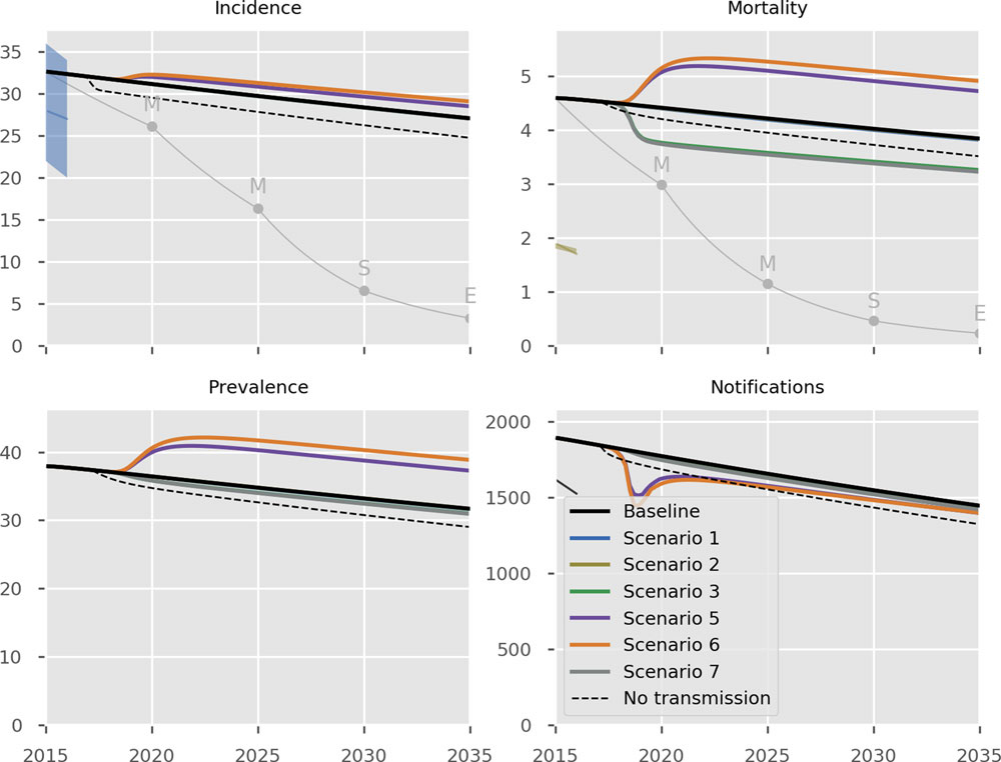
Predicted effect of interventions on overall epidemic. Black, baseline; thin grey, the SDG targets for 2030, the End TB Strategy targets for 2035 and the interim milestones for 2020 and 2025; light blue, short course regimen for MDR-TB (Scenario 1); yellow, scale-up DST coverage in culture-positive TB cases (Scenario 2); green, scale-up food vouchers for all patients under treatment (Scenario 3); purple, discontinue Open Doors program (Scenario 5); orange, discontinue NGO activities (Scenario 6); thick grey, combination of scenarios 1, 2, 3 and 4 (Scenario 7); dashed black, all transmission completely ceases from 2017 onwards. Note that transitioning from inpatient care to ambulatory care for smear-negative and extrapulmonary DS-TB patients (Scenario 4) is not shown in the Figure as it overlaps with the baseline, given the assumption that the two models of care have comparable health outcomes. Scenario 2 also closely overlaps baseline (in its impact on all forms of TB) and so is not visible. Units of all horizontal axes are calendar years and units of vertical axes are: incidence, per 100 000 per year; mortality, per 100 000 per year; prevalence per 100 000; notifications, absolute number.

**Fig. 3.**
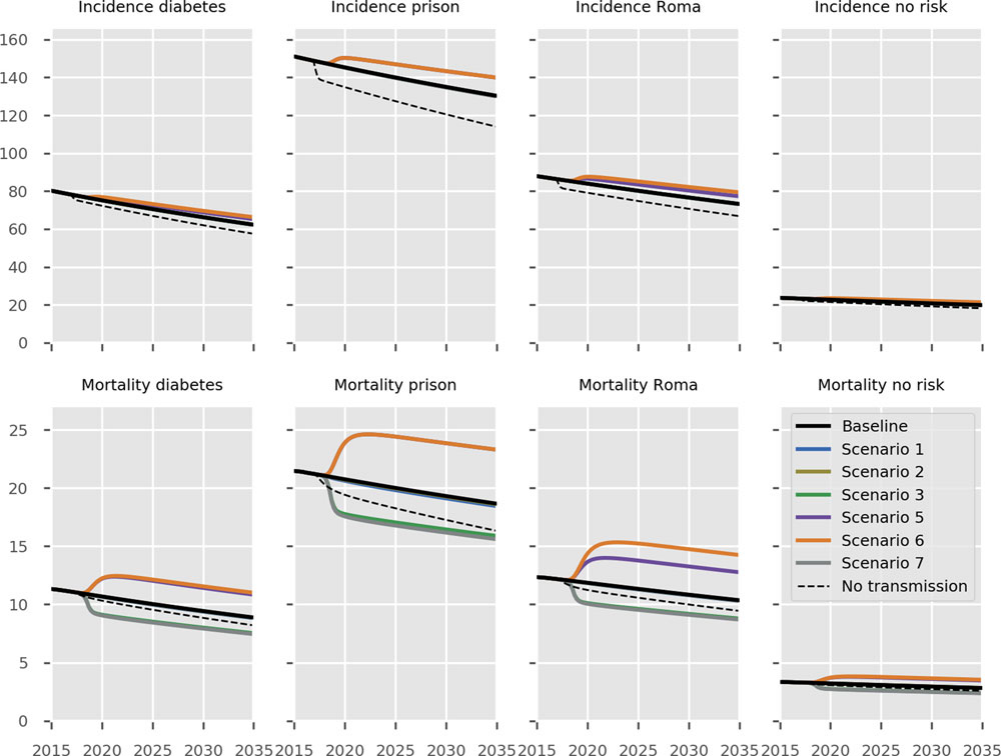
Predicted effect of interventions on disease burden in population risk groups. Black, baseline; light blue, short course regimen for MDR-TB (Scenario 1); yellow, scale-up DST coverage in culture-positive TB cases (Scenario 2); green, scale-up food vouchers for all patients under treatment (Scenario 3); purple, discontinue Open Doors program (Scenario 5); orange, discontinue NGO activities (Scenario 6); thick grey, combination of scenarios 1, 2, 3 and 4 (Scenario 7); dashed black, all transmission completely ceases from 2017 onwards. Note that transitioning from inpatient care to ambulatory care for smear-negative and extrapulmonary DS-TB patients (Scenario 4) is not shown in the Figure as it overlaps with the baseline, given the assumption that the two models of care have comparable health outcomes. No risk refers to the remainder of the population not belonging to one of the three simulated risk groups. Units of all horizontal axes are calendar years and units of all vertical axes are events per 100 000 per year.

**Fig. 4.**
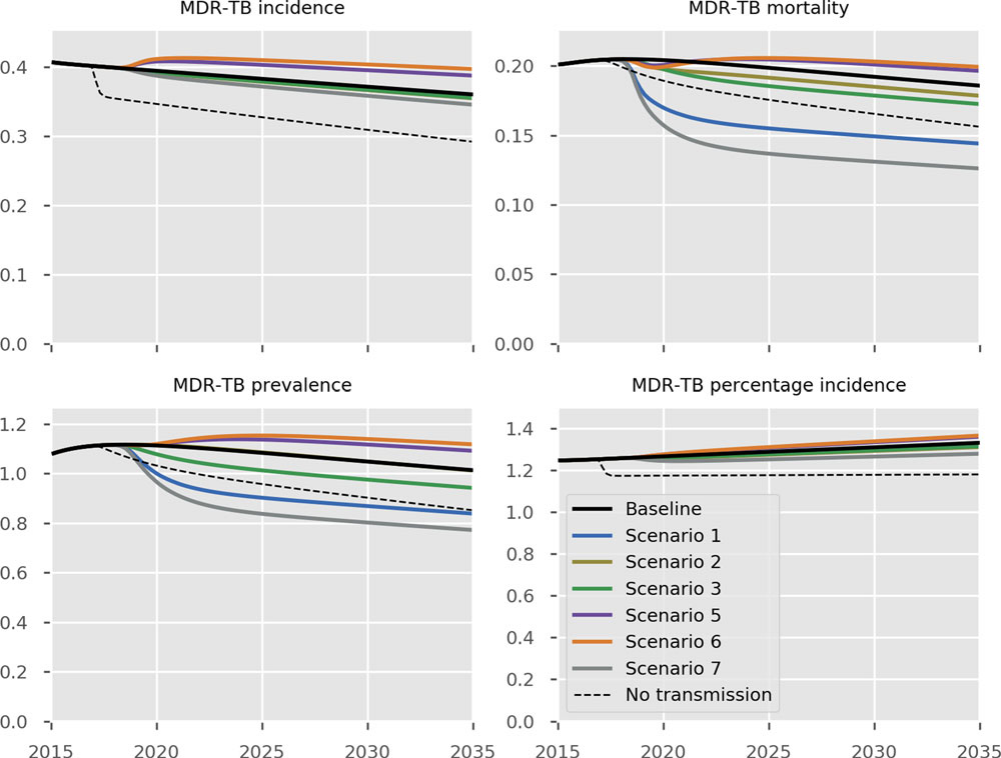
Predicted effect of interventions on the burden of MDR-TB. Black, baseline; light blue, short course regimen for MDR-TB (Scenario 1); yellow, scale-up DST coverage in culture-positive TB cases (Scenario 2); green, scale-up food vouchers for all patients under treatment (Scenario 3); purple, discontinue Open Doors program (Scenario 5); orange, discontinue NGO activities (Scenario 6); thick grey, combination of scenarios 1, 2, 3 and 4 (Scenario 7); dashed black, all transmission completely ceases from 2017 onwards. Units of all horizontal axes are calendar years and units of vertical axes are: MDR-TB incidence, per 100 000 per year; MDR-TB mortality, per 100 000 per year; MDR-TB prevalence per 100 000; notifications, absolute number; MDR-TB percentage incidence, percentage.

The predicted impact of the simulated interventions on the overall TB epidemic is presented in Figure 2, on specific risk groups in Figure 3 and on the MDR-TB epidemic specifically in Figure 4. Shifting to a short course regimen for MDR-TB is predicted to have the greatest impact on MDR-TB prevalence and mortality specifically, although the impact on the overall epidemic is negligible due to the small proportion of all cases contributed by this strain. Expanding coverage of DST has a negligible effect on overall burden and a modest reduction in MDR-TB mortality, due to its already comprehensive coverage. Scaling up the food voucher intervention has the greatest positive effect on overall mortality of any intervention, and also has a marked positive effect on MDR-TB mortality. This intervention is predicted to reduce overall TB mortality to 3.03 deaths per 100 000 per year in 2035 (*vs.* 3.71 under baseline conditions). Discontinuing the Open Doors program would markedly affect TB burden indicators, increasing incidence by 5.7% in 2035 relative to baseline conditions. Discontinuation of all NGO activities has a comparable effect to that of stopping the Open Doors program, with additional adverse effects on Roma communities. Under either the cessation of Open Doors or the cessation of all NGO activity scenario, notifications would fall due to decreased case detection, masking the true impact of these programmatic changes. Combining interventions 1, 2, 3 and 4 would modestly reduce overall TB incidence by 0.3% and substantially reduce mortality by 20% in 2035 compared to baseline. However, under such an ambitious scenario, the country would still fall short of meeting the End TB Strategy targets.

### Costs

**Fig. 5.**
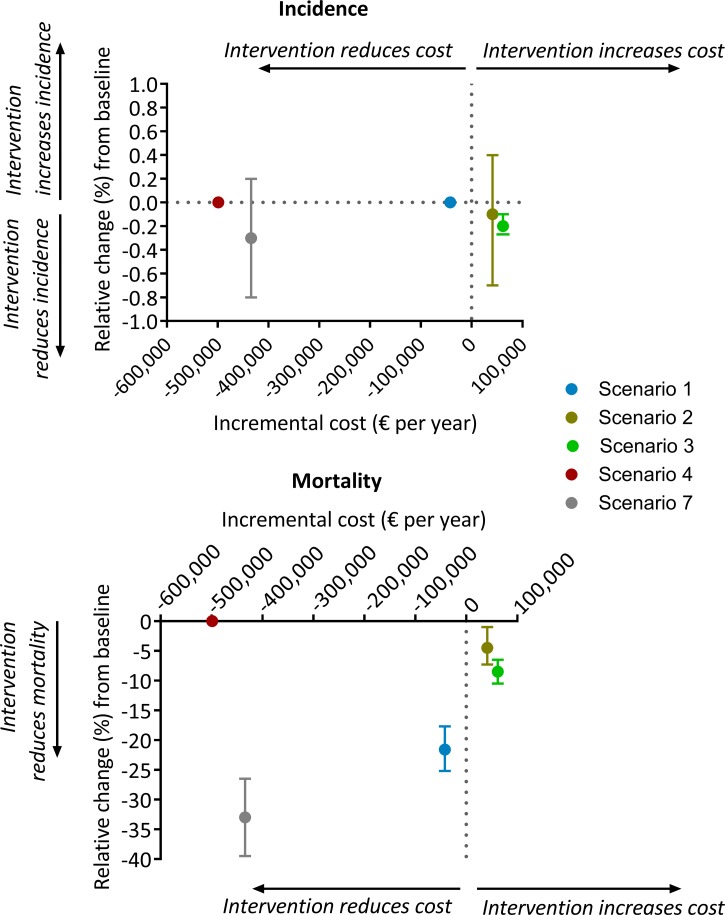
Comparison of baseline cost *vs.* scenario cost for each intervention. All costs are yearly cost in euros. Scenario 1, short course regimen for MDR-TB; scenario 2, scale-up DST coverage in culture-positive TB cases; scenario 3, scale-up food vouchers for all patients under treatment; scenario 4, transitioning from inpatient care to ambulatory care for smear-negative and extrapulmonary DS-TB patients; scenario 7, combination of scenarios 1, 2, 3 and 4. Negative and positive cost values indicate reduction and increase, respectively, of scenario costs relative to baseline. Negative and positive percentages indicate reduction and increase, relatively, of overall TB incidence (top panel) or overall TB mortality (bottom panel) relative to baseline.

The results of the economic analysis are shown in Table 2 and Figure 5. Scale-up of phenotypic DST availability from 86 to 100% and expansion of the food voucher program to all patients under treatment are both found to be highly affordable interventions, with total programmatic costs below € 100 000 per year. Short course MDR-TB treatment was markedly cost-saving. Shifting from the existing model of primarily inpatient care to a predominantly ambulatory approach for non-smear-positive patients would result in considerable cost savings.

**Table 2.** Predicted epidemiological and financial outcomes of interventions

Intervention	TB incidence in 2035		MDR-TB incidence in 2035		TB mortality in 2035		Mortality MDR in 2035		Additional intervention costs or savings
	Per 100 000 per year	Relative change (%)	Per 10 000 000 per year	Relative change (%)	Per 100 000 per year	Relative change (%)	Per 100 000 per year	Relative change (%)	€ per year
Baseline	26.17	Reference	35.85	Reference	3.71	Reference	0.184	Reference	Reference
1. Short course regimen for MDR-TB	26.16 (26.16 to 26.16)	−0.0 (−0.0 to −0.0)	35.36 (35.30 to 35.42)	−1.4 (−1.5 to −1.2)	3.69 (3.69 to 3.70)	−0.6 (−0.8 to −0.4)	0.144 (0.137 to 0.151)	−21.6 (−25.2 to −17.7)	−42 115
2. Scale-up DST coverage	26.14 (25.99 to 26.28)	−0.1 (−0.7 to 0.4)	35.18 (34.62 to 35.87)	−1.9 (−3.4 to 0.1)	3.69 (3.63 to 3.75)	−0.4 (−2.0 to 1.2)	0.175 (0.170 to 0.181)	−4.5 (−7.3 to −1.0)	40 942
3. Scale-up food vouchers to reach all patients under treatment	26.12 (26.11 to 26.13)	−0.2 (−0.2 to −0.1)	35.25 (35.10 to 35.38)	−1.7 (−2.1 to −1.3)	3.03 (2.86 to 3.19)	−18.4 (−22.9 to −14.2)	0.168 (0.164 to 0.172)	−8.5 (−10.5 to −6.5)	61 756
4. Ambulatory care for smear-negative and extrapulmonary DS-TB	No change	No change	No change	No change	No change	No change	No change	No change	−498 514^a^
5. Discontinue Open Doors	27.67 (27.08 to 28.27)	+5.7 (3.5 to 8.0)	38.77 (37.64 to 39.89)	+8.1 (5.0 to 11.2)	4.63 (4.27 to 5.00)	+24.5 (14.9 to 34.6)	0.195 (0.191 to 0.200)	+6.2 (3.8 to 8.6)	NA
6. Discontinue NGO activities	28.24 (27.42 to 29.14)	+7.9 (4.8 to 11.4)	39.71 (38.23 to 41.30)	+10.8 (6.6 to 15.2)	4.79 (4.38 to 5.27)	+29.0 (18.1 to 42.0)	0.198 (0.193 to 0.204)	+7.7 (4.8 to 11.1)	NA
7. Combined (1, 2, 3 and 4)	26.08 (25.95 to 26.23)	−0.3 (−0.8 to 0.2)	34.25 (33.61 to 34.89)	−4.4 (−6.2 to −2.7)	2.97 (2.82 to 3.18)	−20.1 (−24.1 to −15.5)	0.124 (0.112 to 0.134)	−33.0 (−39.1 to −26.5)	−434 154

DS, drug-susceptible; DST, drug susceptibility testing; MDR, multidrug-resistant; NA, not applicable; NGO, non-governmental organisation; TB, tuberculosis.

The ranges reported in brackets are the 95% simulation intervals obtained when incorporating uncertainty by using 50 samples from a Latin hypercube sampling approach to vary the parameters characterising the intervention effects only (while holding all epidemiological values to their calibrated values).

a Predicted difference between the annual cost of ambulatory care (439 308€ per year) and the annual cost of current care programs (937 822€ per year) for smear-negative and extrapulmonary patients.

## Discussion

We project that continuing the current programmatic TB response in Bulgaria will result in a steady but modest decline in overall disease burden. This would fall far short of meeting the country's ambitious targets for mortality and incidence for 2025 or global targets for both indicators for 2035. Food vouchers for all patients under treatment are likely to have the greatest impact on reducing TB burden, and in particular TB mortality. The discontinuation of NGO activities and the Open Doors program would result in a considerable increase in both overall TB and MDR-TB burden. Therefore, maintaining these two interventions is necessary to sustain the progress in TB control that has been made in the country. We also found that ambulatory care is cost saving while having no impact on epidemiology of TB, and that short course therapy for MDR-TB is both cost saving and reduces MDR-TB burden. Under an ambitious scenario in which interventions 1, 2, 3 and 4 are combined, overall TB and MDR-TB burden would decline substantially; however the End TB Strategy targets would still not be achieved.

Bulgaria is considering a transition from hospital-based TB management to a comprehensive ambulatory TB care program. Previous studies have consistently shown that there is no significant difference in treatment outcomes between the two models of care [22, 25]. Consistent with this, in our model we simulate no parameter changes as a consequence of changing to this approach. Nevertheless, from an individual patient's perspective, evidence suggests that ambulatory care is preferable to hospital-based care by most patients [24]. In our economic analysis, we found that ambulatory care was cheaper than hospital-based care (hence cost-saving) and so is a logical programmatic choice.

In our economic analysis of replacing the conventional MDR-TB regimen with a short-course regimen, we focused on the financial impact of this change rather than on the overall financial burden of MDR-TB. Although MDR-TB only accounts for 1.2% of all TB cases in Bulgaria, its economic burden is likely to be more substantial due to the much higher costs of diagnosis and treatment. Assuming similar results can be achieved to those observed in Bangladesh, Cameroon and Niger [16–18], we estimate that replacing the conventional MDR-TB treatment regimen with a short course approach would substantially reduce MDR-TB burden and be cost-saving. As such, implementing a short course regimen for MDR-TB and ambulatory care is anticipated to improve the efficiency of the TB program.

Our model simulates a low-transmission epidemic with most TB cases arising from late reactivation of distantly acquired infections. Despite this, the simulated proportion of the population with latent infection has declined only very slowly over recent decades (Fig. S23), which is the explanation for the limited impact of several interventions. Specifically, all programmatic changes affect interventions directed at active cases and have no effect on the latent pool. Figures 2–4 and Figure S24 show the predicted epidemiological results of immediate and complete cessation of transmission, indicating that incidence persists at considerable rates long after this is implemented. Similarly, combining interventions 1, 2, 3 and 4 would still be insufficient for the country to meet the End TB Strategy targets. These ambitious scenarios highlight the importance of the pool of latent infection in continuing to drive the epidemic in Bulgaria. Consistent with this, previous studies have shown that addressing the pool of LTBI through diagnosis and preventive treatment is critical to achieving TB elimination [26–28].

To date, there remains a lack of strong evidence from published literature on the epidemiological impact of scale-up of DST coverage (intervention 2), Open Doors program (intervention 5) and NGO activities (intervention 6). Given this, it is important to balance the need to assess all real-world programmatic interventions currently being considered by the Bulgarian NTP against the standard of evidence available to inform each intervention. Therefore, our philosophical approach was to provide estimates of all such interventions under consideration, but also to highlight carefully the varying strength of evidence to support each one. To our knowledge, this has rarely been undertaken in previous modelling studies, such that we believe this study presents a significant advance over past programmatic modelling studies in this respect. We obtained information about the impact of these interventions from empirical data, in consultation with the country NTP. Data gathering, synthesis and interpretation were performed through in-country missions, in close consultation with key country stakeholders. As such, we believe that the model inputs for these interventions are as robust as possible, but the estimates of the impact of each intervention should be considered carefully in the context of the strength of the supporting evidence.

Although we represented TB transmission using a highly complex model, limitations arising from this level of complexity include that interpretation of every dynamic process occurring within the model is impossible. This is offset by a more realistic simulation of the epidemic's characteristics, such as its concentration among risk groups. Despite this, the model also remains a simplification of reality. For example, each simulation is deterministic, such that stochastic fluctuations due to variation in rates of transmission, reactivation or other processes are not captured. However, deterministic, compartmental models of TB transmission dynamics are an established tool for estimating the epidemic trajectory for TB at the country level and predicting the impact of interventions [29]. For simplicity, persons with diabetes mellitus, the prison population and Roma communities were considered to be mutually exclusive risk groups in our model. While it is possible that an individual could simultaneously fall into more than one of these risk groups (e.g. being a prisoner and having diabetes mellitus), allowing for this possibility would substantially increase the complexity of the model and make it overly computationally expensive. In this study, the economic analysis was conducted from a health provider perspective. As such, we did not include patient costs such as travel costs, and opportunity costs associated with time off work and income loss. If a broader perspective, such as a societal perspective, was used and these additional costs included, we anticipate that the economic benefits of the food vouchers, ambulatory care and short-course regimen for MDR-TB programs would appear even more favourable.

The findings of this study are useful for strategic planning for TB control in the country to ensure efficient and sustainable provision of TB care beyond the end of the Global Fund grant. At the time of writing, the country was developing a financing mechanism drawing state budget to fund the continuation of NGO activities and the Open Door program, and the implementation of ambulatory care program and short-course regimens for MDR-TB treatment. As part of the transition to domestically-funded care, the Bulgarian government has increased its budget contribution for TB programs to more than 90%. Additional initiatives are being undertaken to further improve the efficiency and sustainability of TB programs. These include financing system reform, the integration of TB services into general pulmonary health services, the incorporation of TB care into the National Health Map planning tool and the increased utilisation of the health information system and quantitative analysis to inform TB policy, to name a few.

In conclusion, if current programmatic responses are continued, we predict further gradual declines in TB burden in Bulgaria, although these will be well short of the SDGs and End TB Strategy targets. Finding and treating TB cases in high-risk groups such as Roma communities through the activities of NGOs and Open Doors program are important components of reducing TB burden. Addressing the underlying burden of LTBI will also be important if the country is to achieve its disease-related goals.

## Supplementary material

For supplementary material accompanying this paper visit https://doi.org/10.1017/S0950268819001857. click here to view supplementary material
