# Supplementary material for: Strategic investment in tuberculosis control in the Republic of Bulgaria
Source: Epidemiol Infect. 2019 Nov 18;147:e304. doi: 10.1017/S0950268819001857 (PMC6873158; doi:10.1017/S0950268819001857)
Supplement: Supplementary file 1 [file S0950268819001857sup001.docx]

*Epidemiology and Infection*

**Strategic investment in tuberculosis control in the Republic of Bulgaria**

T. N. Doan, T. Varleva, M. Zamfirova, M. Tyufekchieva, A. Keshelava, K. Hristov, A. Yaneva, B. Gadzheva, S. Zhang, S. Irbe, R. Ragonnet, E. S. McBryde, J. M. Trauer

**Supplementary Material**

# General approach

This Supplement describes our approach to simulating the TB epidemic in Bulgaria. We introduced this approach in our earlier publication which focused on the computational and technical aspects of our approach.^1^ This previous publication presents the structure of the code base (including providing the code framework as an open access repository), the principles for how model compartments interact and the details of the logistic function that relates intervention spending to coverage.

Briefly to introduce the epidemiological model, we developed a flexible modelling platform that first divides the population into compartments concerning their status in relation to *Mycobacterium tuberculosis* (*Mtb*) infection and TB disease. Specifically, these are: fully susceptible, partially immune susceptible, early latent, active, detected, missed, early treatment, late treatment, recovered and receiving preventive therapy population groups (or compartments). The entire population is stratified according to age groups and treatment history groups (new and retreatment), with these stratifications applied to all population groups and compartments, such that these two stratifications are fully multiplicative (except that there can be no fully susceptible retreatment group). Risk group stratification is applied to all age groups, except that no prisoners under 15 and no diabetics under 25 are allowed, but is otherwise a multiplicative stratification. Stratification by access to care of variable quality (which is a previously used option of the AuTuMN platform) is not applicable to this application to Bulgaria. Stratification by organ manifestation (smear-positive, smear-negative and extrapulmonary) is applied only to the compartments that represent active disease, and hence is not applied to the susceptible, latently infected and recovered groups.

Model parameters may be time-variant and informed by reported data over multiple time points or constant throughout a model run. Of the constant parameters, some are varied from run to run during the calibration process, while the remainder remain fixed throughout all simulations. This is intended to capture a high degree of historical consistency with TB epidemiology in Bulgaria, incorporating both TB-related and demographic aspects.

Last, our approach to calibration allows for variation in highly uncertain epidemiological quantities relevant to TB transmission, permitting both model calibration and quantification of the uncertainty around baseline extrapolations of the trajectory of TB burden markers.

By contrast, to quantify uncertainty around the effects of interventions, we do not consider the uncertainty around disease-specific model parameters, aiming instead to isolate and quantify the uncertainty relating to the evidence for the interventions alone. This is achieved by varying a small number of parameters specifically related to the intervention of interest using a Latin Hypercube sampling approach to fully explore their plausible ranges. This process of quantifying uncertainty in intervention impact is undertaken after model calibration has been completed and the final calibration values of these parameters remain fixed throughout this intervention uncertainty process.

# Parameter types

There are five broad types of parameters implemented:

1. Constant parameters
   - These parameters are set to a constant value, both throughout historical time during the course of a model run, over the series of model runs constituting an uncertainty simulation and throughout intervention uncertainty.
2. Calibration parameters
   - These parameters are a limited set of time-invariant parameters (five in this application), which are varied between consecutive runs of the model during the process of calibration and estimation of epidemiological uncertainty.
3. Time-variant parameters
   - Functions mapping parameter values to time are created by fitting to multiple data points provided for different times. This approach allows programmatic parameters to vary in a way that reflects the programmatic history of TB control in Bulgaria and so enhances realism.
   - Polynomial spline fitting techniques are used to fit curves to available data and presented in Section 3.
4. Calculated parameters
   - Some parameters are calculated from simulated quantities at each time step of model integration. In this application, this only applies to the forces of infection for each population group that is susceptible to infection and for each strain^[[1]](#footnote-1)^ of TB. Frequency-dependent transmission and homogeneous mixing is assumed, such that the force of infection is proportional to a weighted proportion of all groups with active TB of the relevant strain in Bulgaria who have not yet entered the late (non-infectious) phase of treatment. The force of infection is modified for persons who are already infected and in late latency, for BCG-vaccinated persons and for persons who have previously completed treatment for active disease (with the parameters reflecting partial immunity being fixed for the first two of these groups and varied with calibration for the third).
5. Intervention uncertainty parameters
   - A small number of parameters pertaining to a single intervention whose uncertainty is being quantified are varied using a Latin Hypercube sampling approach. This is done only from the point that the intervention commences onwards and is applied to the previously calibrated model.

# Scale-up functions

This section presents the parameter values included within the model that vary with time according to fitting of functions to publicly available data from Bulgaria for the quantities considered. (As described in Section 2 above, the only time-variant parameter that do not fall into this category are the strain-specific forces of infection, which varies over time with the proportion of persons manifesting active infectious TB of each strain.)

For each of the following figures, the blue circles represent the data points fitted to (data sources vary as noted in figure captions) and the black lines represent the parameter value used in model integration by calendar year. The left and right panels present the same data and only differ in the range of the x-axis (time) displayed. The effective value of some of these parameter values (e.g. case detection rate) may be changed during the analysis of specific scenarios (usually after 2016), which is not illustrated in the figures below.

Bounded polynomial spline functions are used for data fitting, where the range of the function is restricted as appropriate. That is, the range of fitted functions is limited to positive values for all functions and to values from zero to one for proportions.


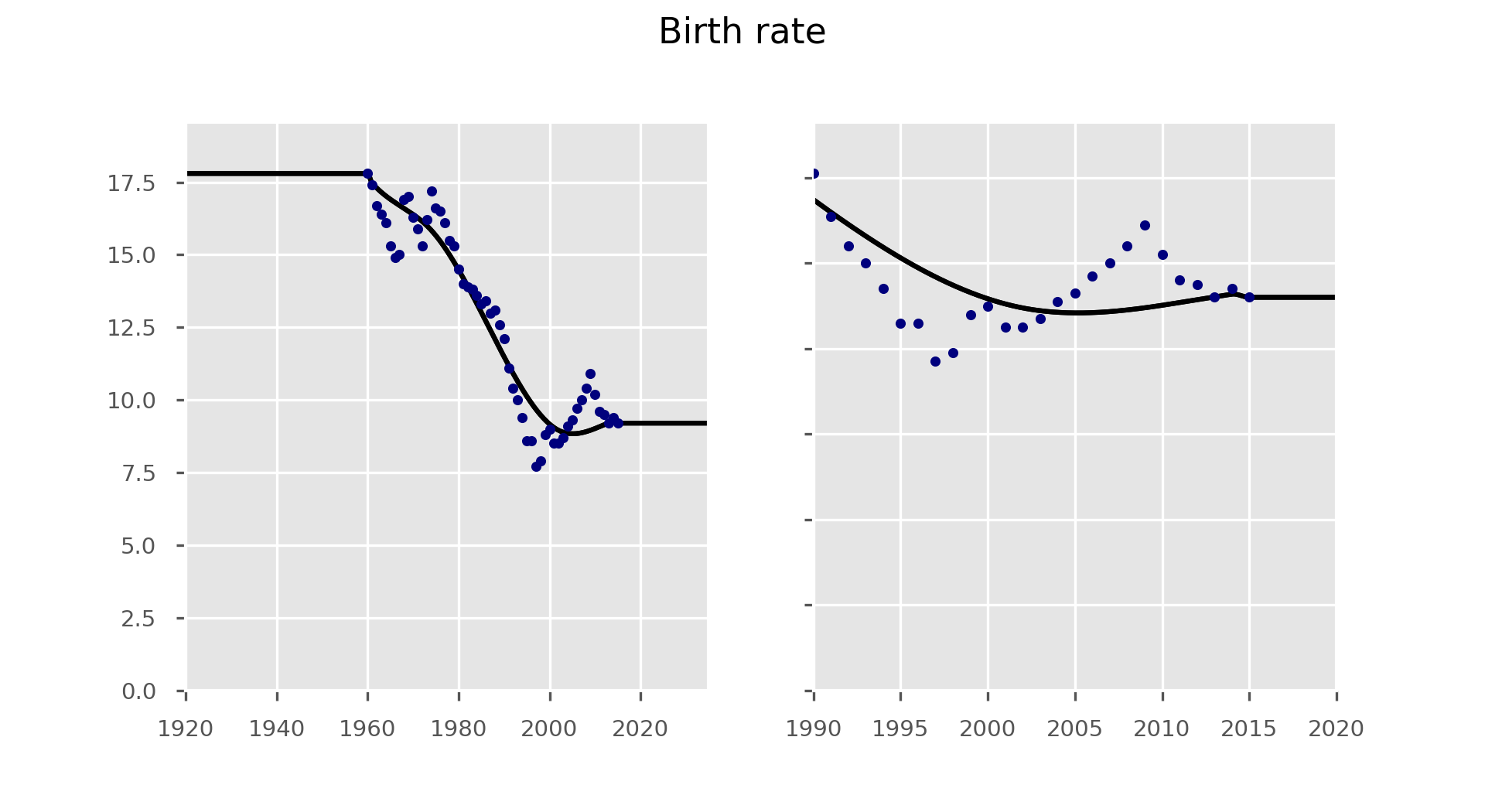


#### **Figure S1. Population per capita birth rate parameter in births per 1,000 population.** Data source: the World Bank 2016.


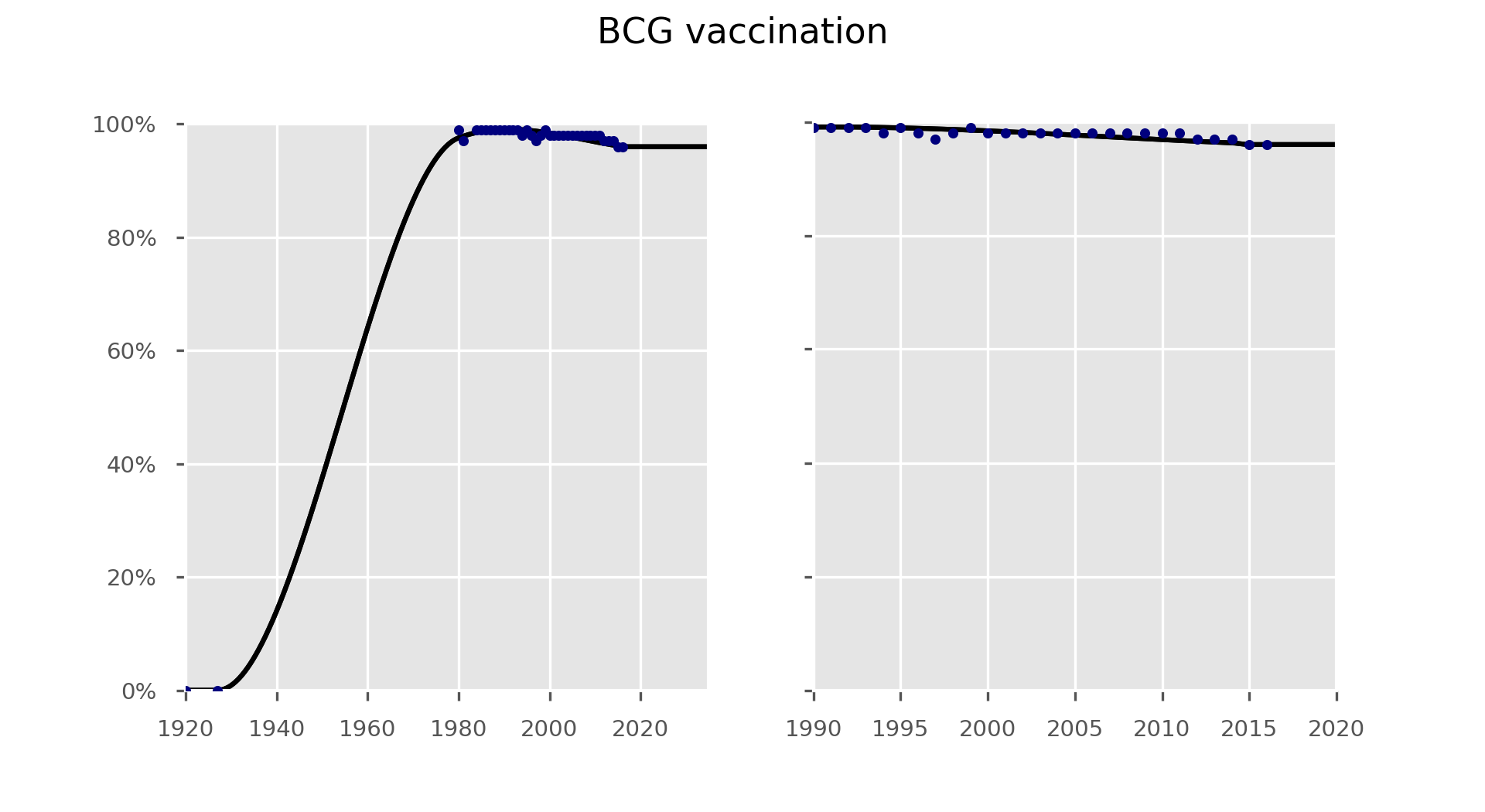


#### **Figure S2. BCG vaccination coverage parameter.** One additional data point added manually (value of zero in 1927) to reflect unavailability of BCG vaccination prior to this date. Data source: WHO/UNICEF.


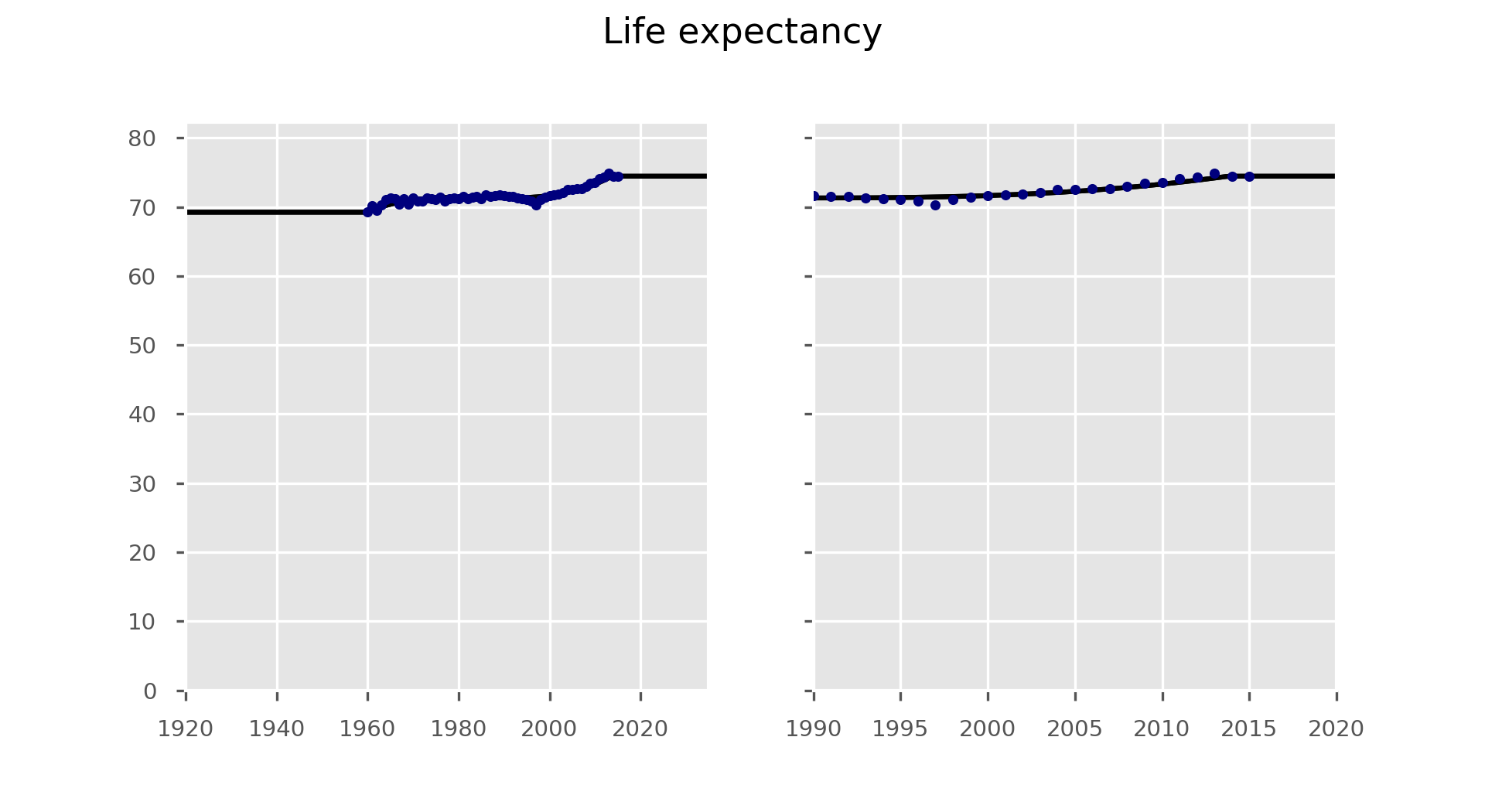


#### **Figure S3. Life expectancy parameter in years.** Data source: the World Bank 2016.


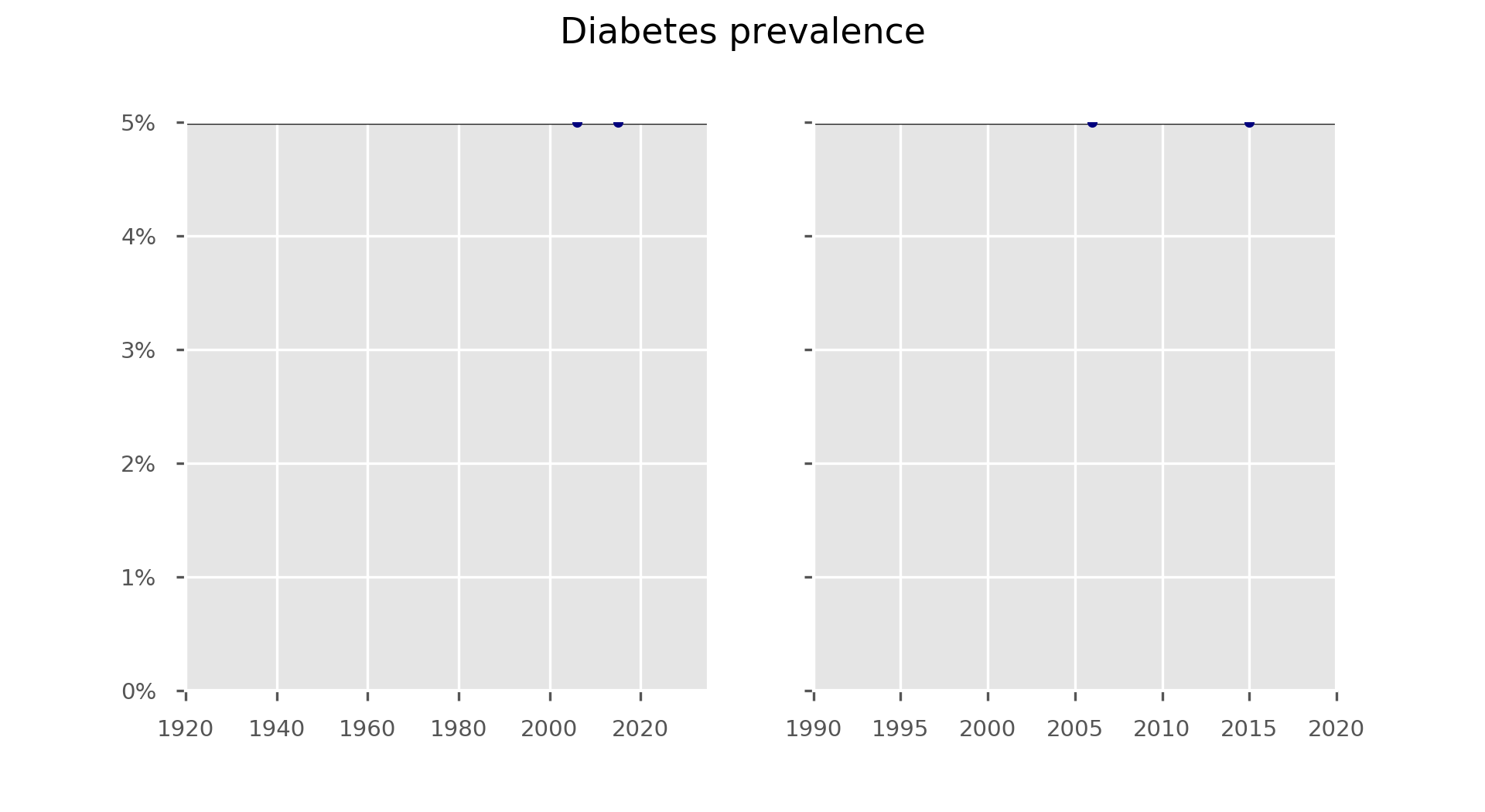


#### **Figure S4. Proportion of population with diabetes mellitus.** Note that the proportion of the population in the other risk groups (Roma and prison populations) is fixed. Sources are published estimates of diabetes burden in Bulgaria^2^ and the International Diabetes Federation Bulgaria country profile.


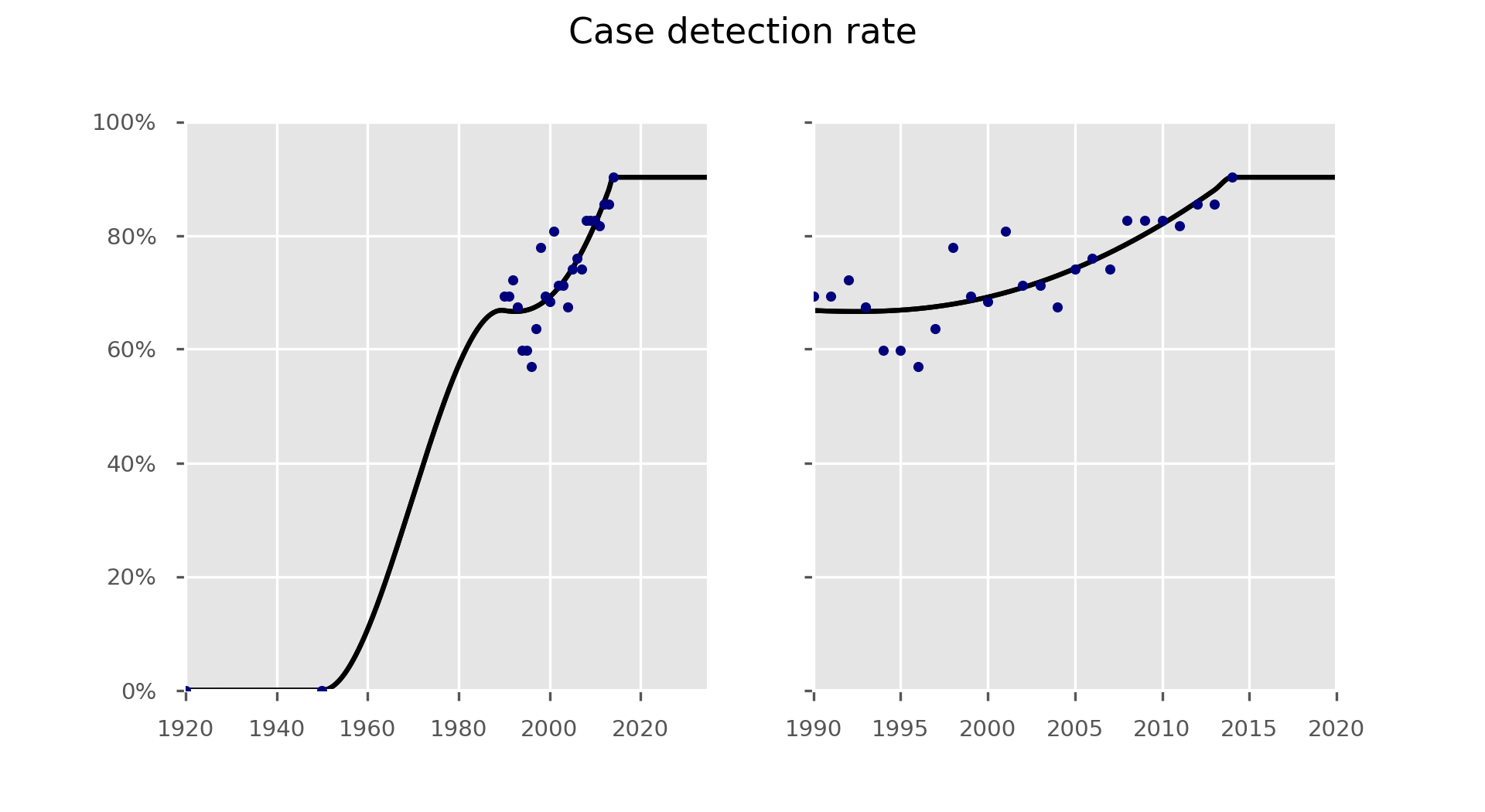


#### **Figure S5. Case detection rate parameter.** One additional value (zero in 1950) added to those available to reflect unavailability of treatment prior to this date. All values were multiplied by 0.95 for model implementation on advice of the country team, given the very high reported case detection rates were considered to be over-estimates. Data source: WHO, Global Tuberculosis Report 2016.


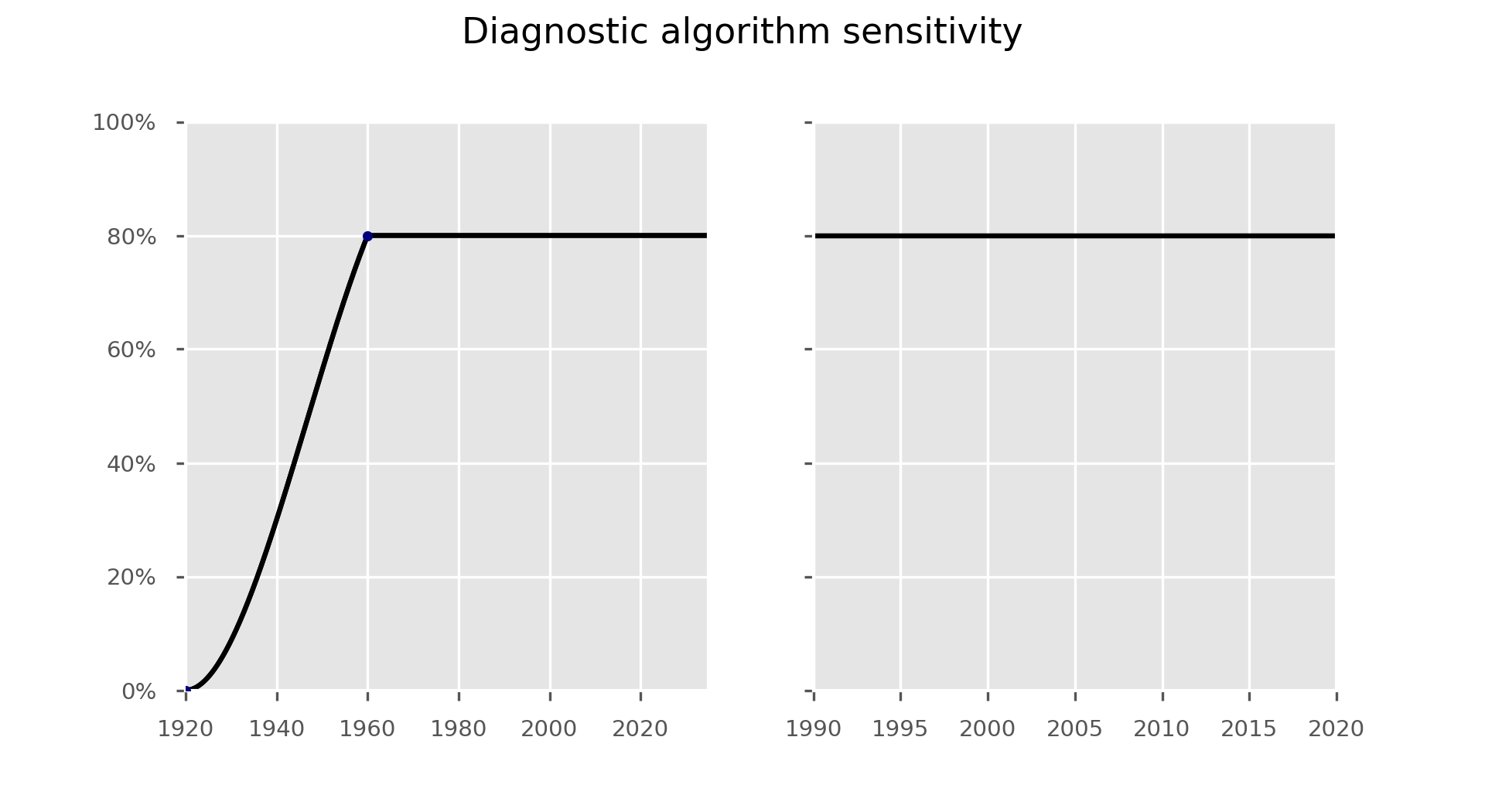


#### **Figure S6. Sensitivity of the diagnostic algorithm parameter.** Note that value of this parameter is irrelevant until case detection rate exceeds zero in 1950.


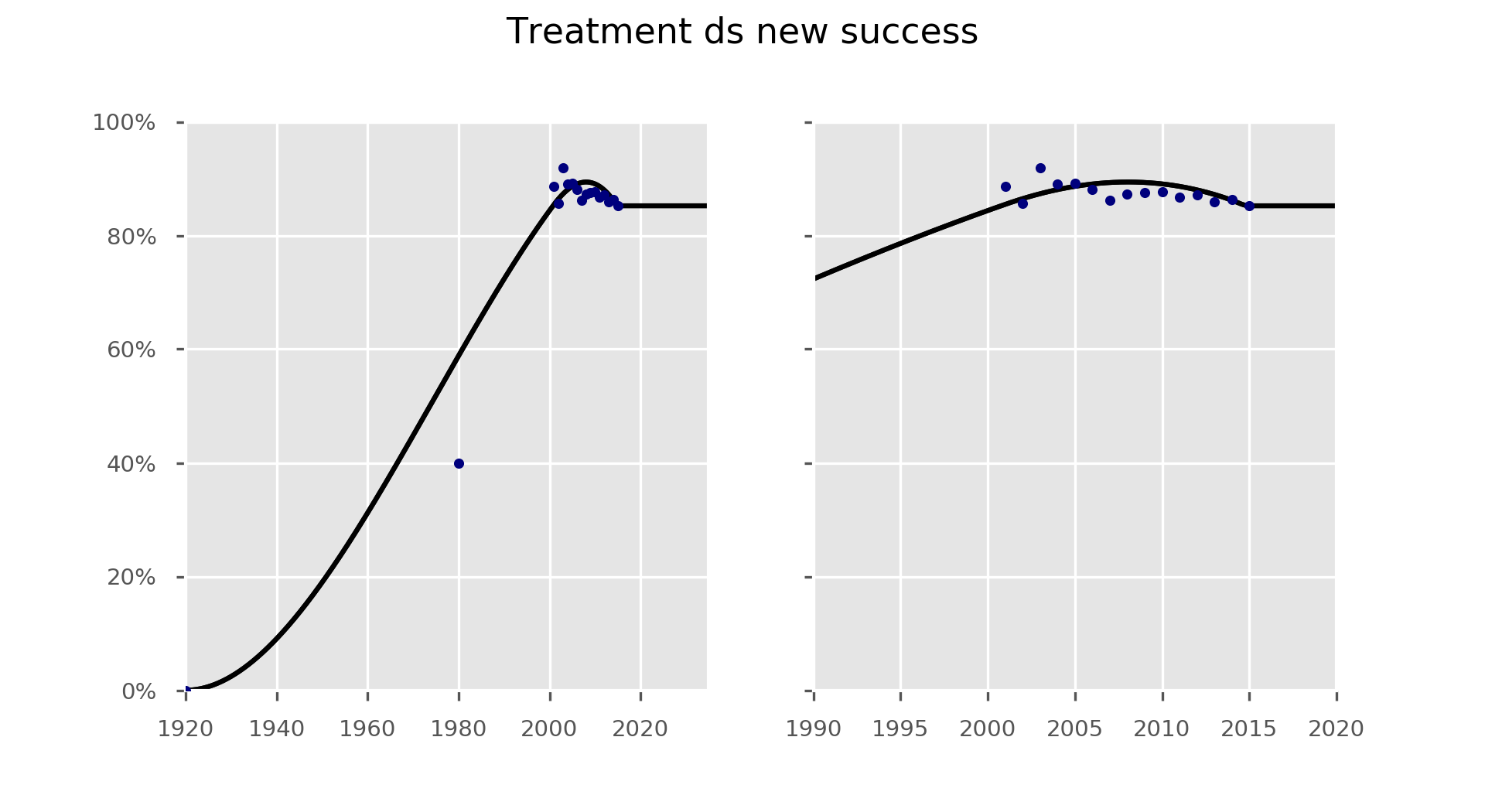


#### **Figure S7. Treatment success outcome for new DS-TB patients parameter.** Data source: WHO, Global Tuberculosis Report 2017. Note that value of this parameter is irrelevant until case detection rate exceeds zero in 1950.


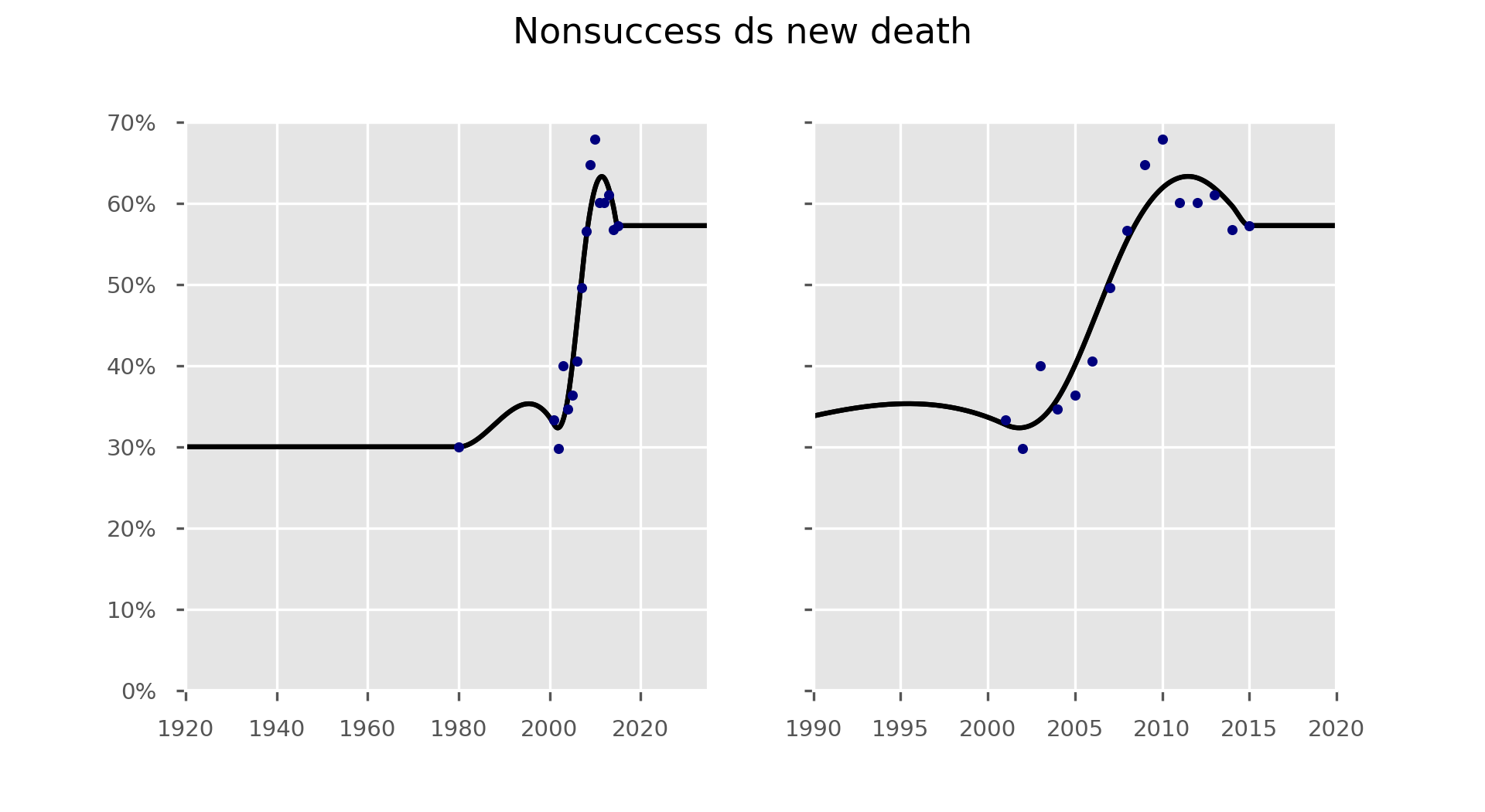


#### **Figure S8. Proportion of unsuccessful outcomes resulting in death for new DS-TB patients parameter.** This parameter is used to calculate the proportion of treatment outcomes resulting in death by multiplying its value by the complement of the treatment success rate. Data source: WHO, Global Tuberculosis Report 2017. Note that value of this parameter is irrelevant until case detection rate exceeds zero in 1950.


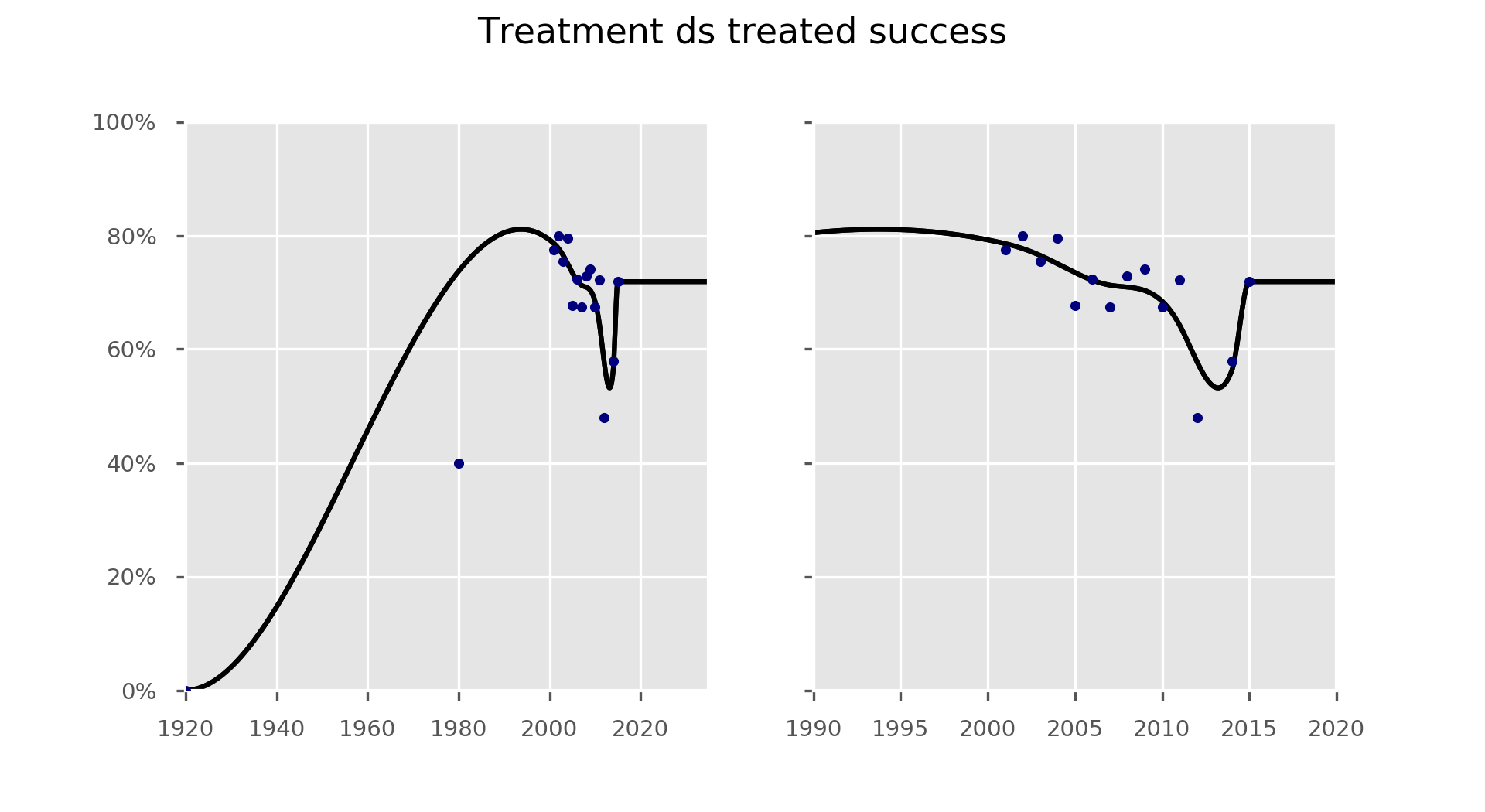


#### **Figure S9. Treatment success proportion for previously treated DS-TB patients parameter.** Data source: WHO, Global Tuberculosis Report 2017. Two additional values added to those available at 1920 and 1980 for historical realism. Note that the value of this parameter is irrelevant until case detection rate exceeds zero in 1950.


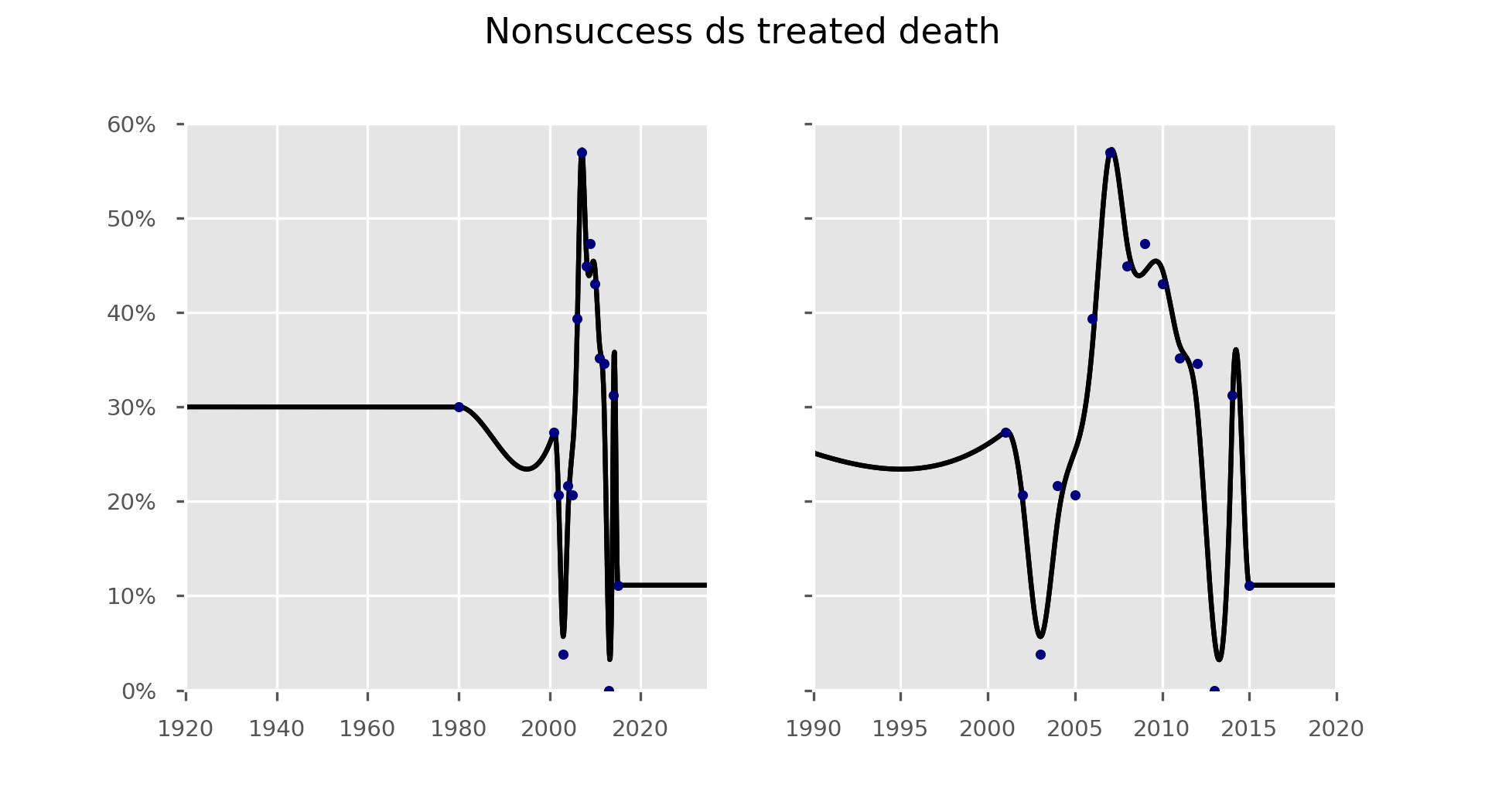


#### **Figure S10. Proportion of unsuccessful outcomes resulting in death on treatment for previously treated DS-TB patients parameter.** This parameter is used to calculate the proportion of treatment outcomes resulting in death by multiplying its value by the complement of the treatment success rate. Data source: WHO, Global Tuberculosis Report 2017. Note that the value of this parameter is irrelevant until case detection rate exceeds zero in 1950.


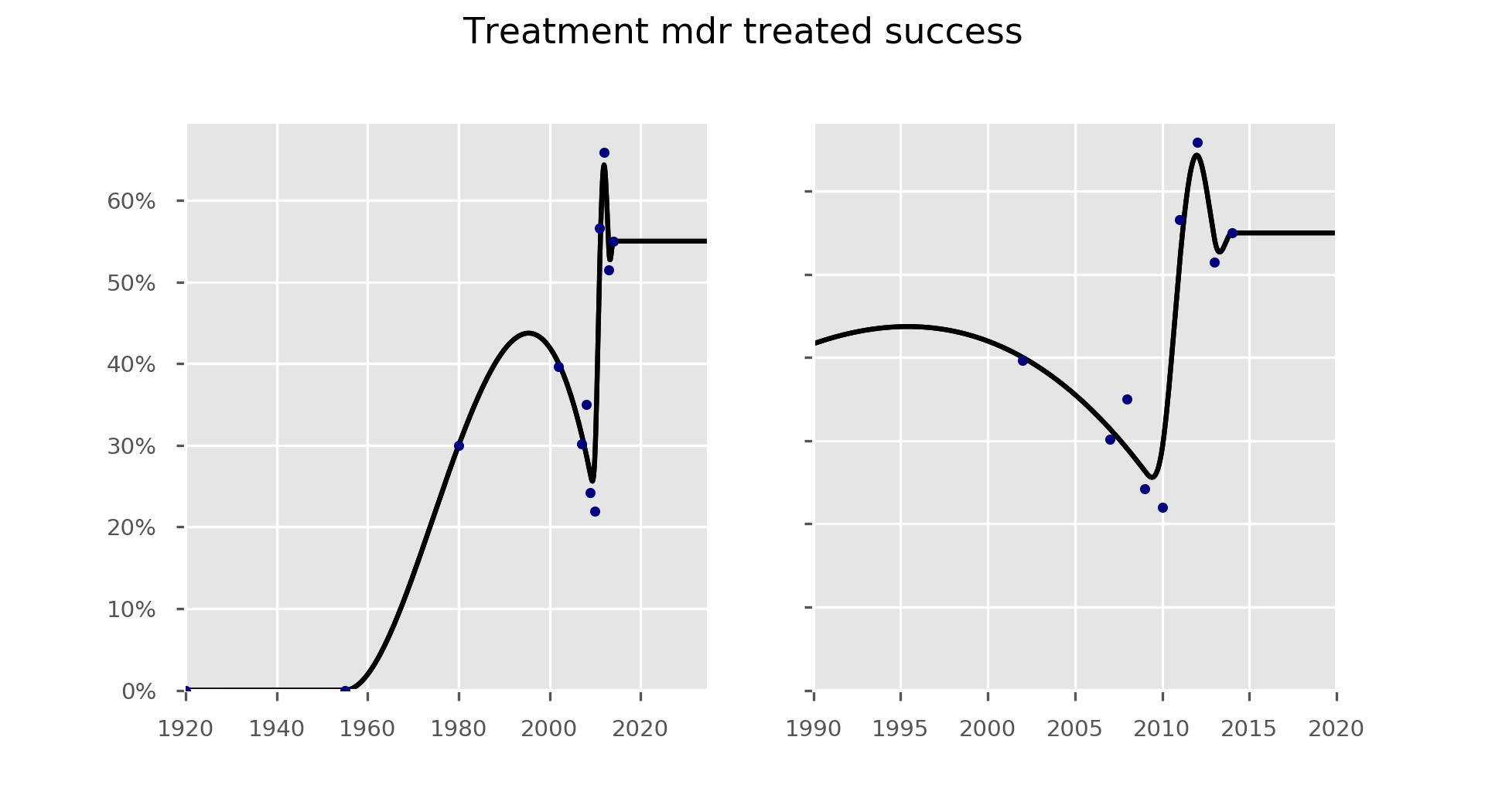


#### **Figure S11. Treatment success proportion for MDR-TB patients.** Parameter applies to both new and re-treatment cases.


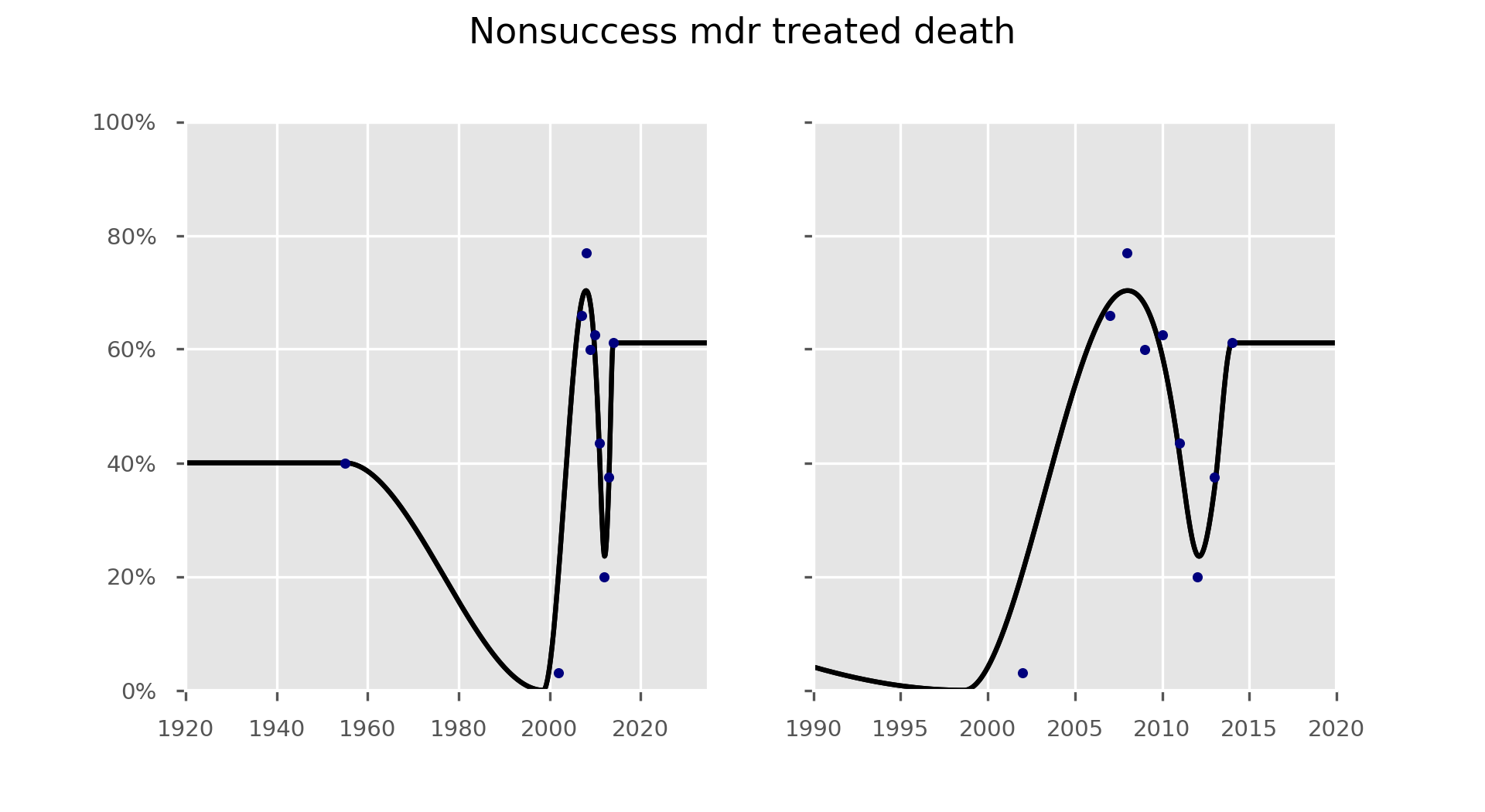


#### **Figure S12. Proportion of unsuccessful outcomes resulting in death on treatment for MDR-TB patients parameter.** Parameter applies to both new and re-treatment cases.


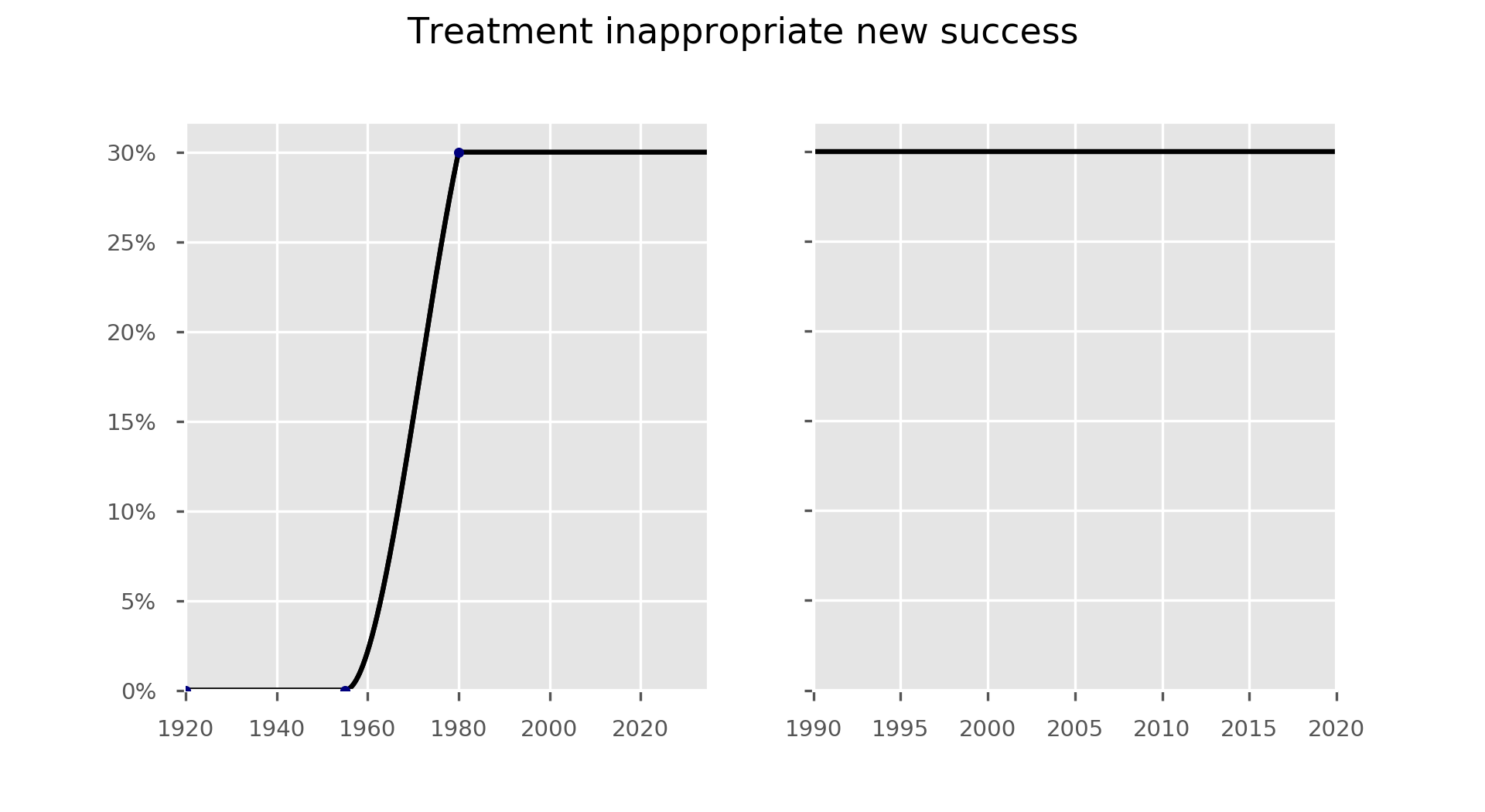


#### **Figure S13. Treatment success proportion for patients on an inappropriate regimen.** In this two-strain application to Bulgaria, inappropriate regimen refers to patients with MDR-TB treated with a standard regimen for DS-TB only. Parameter applies to both new and re-treatment patients.


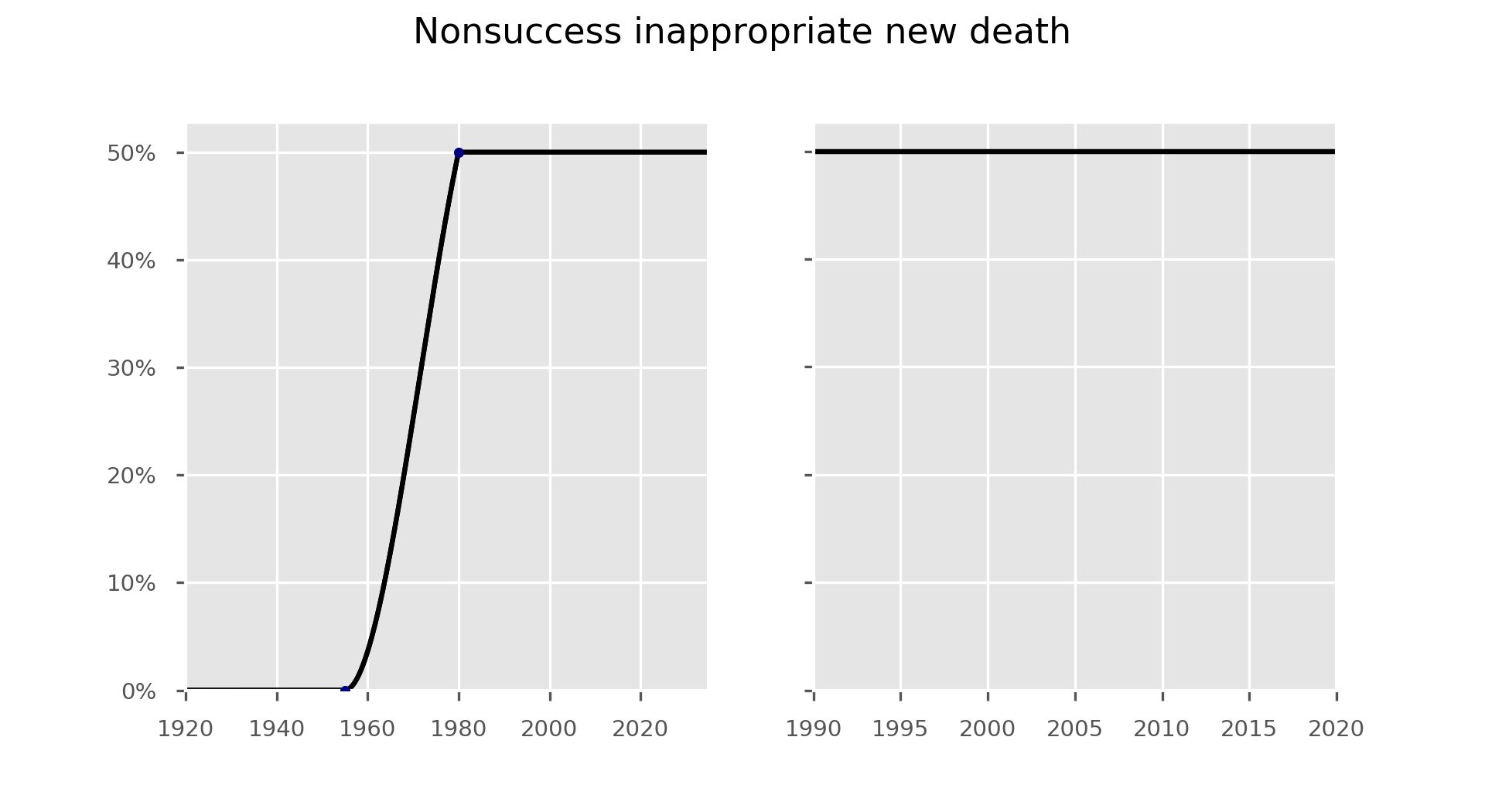


#### **Figure S14. Proportion of unsuccessful outcomes resulting in death on treatment for patients on an inappropriate regimen.** This parameter is used to calculate the proportion of treatment outcomes resulting in death by multiplying its value by the complement of the treatment success rate. Parameter applies to both new and re-treatment patients.


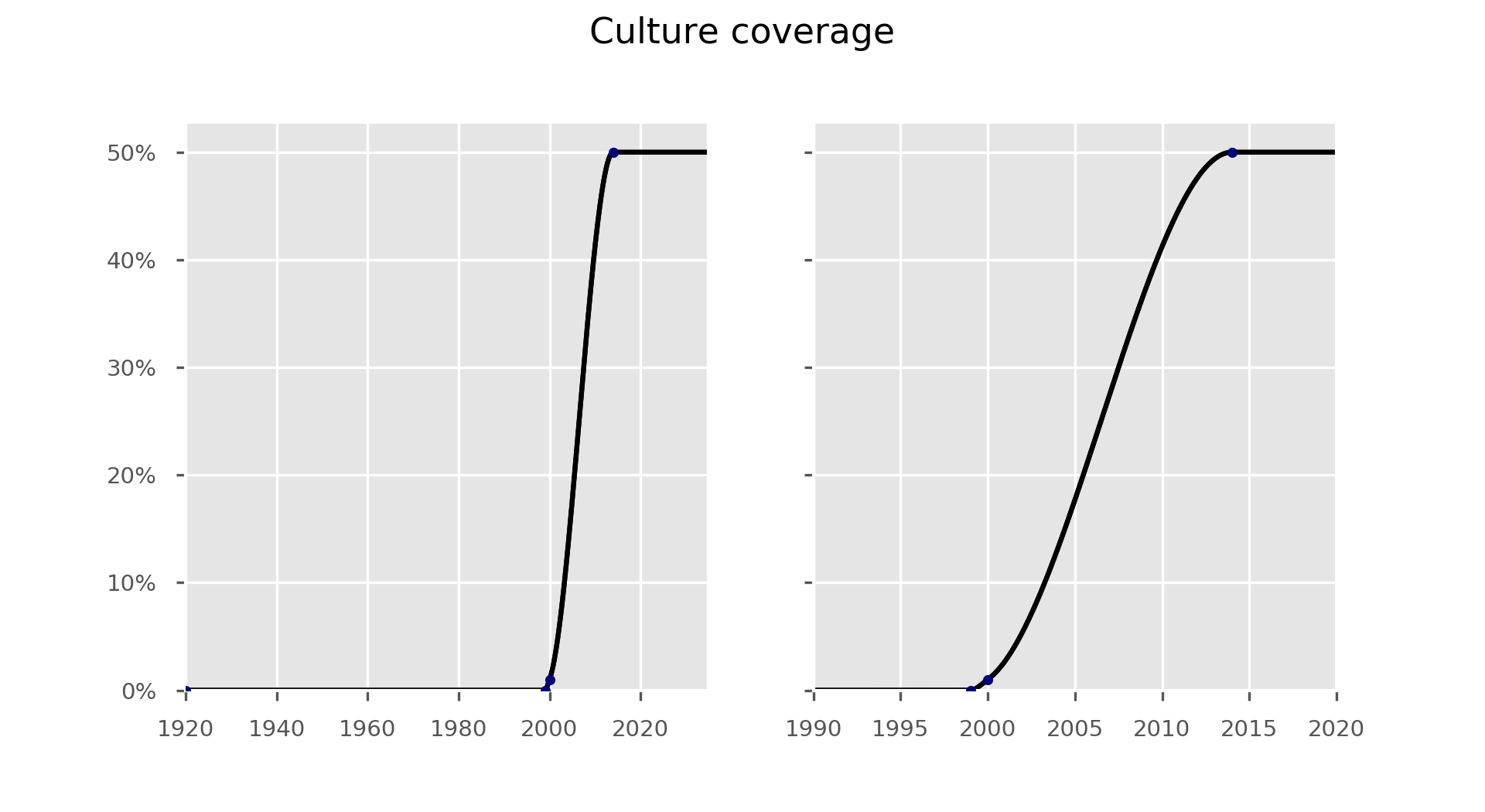


#### **Figure S15. Coverage of Mtb culture testing.**


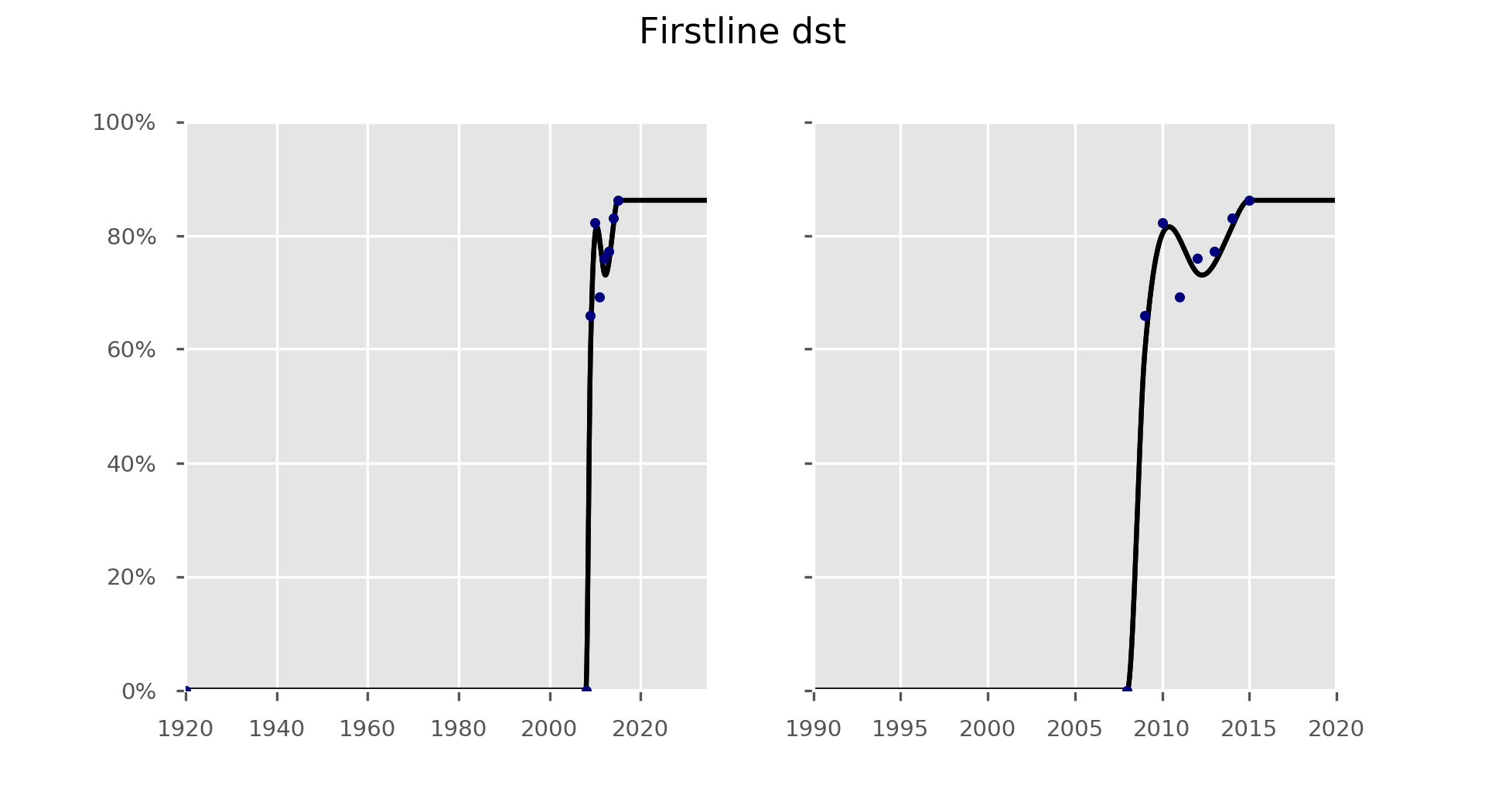


#### **Figure S16. Coverage of first-line drug susceptibility testing.** Applicable only to those for whom culture testing is available.


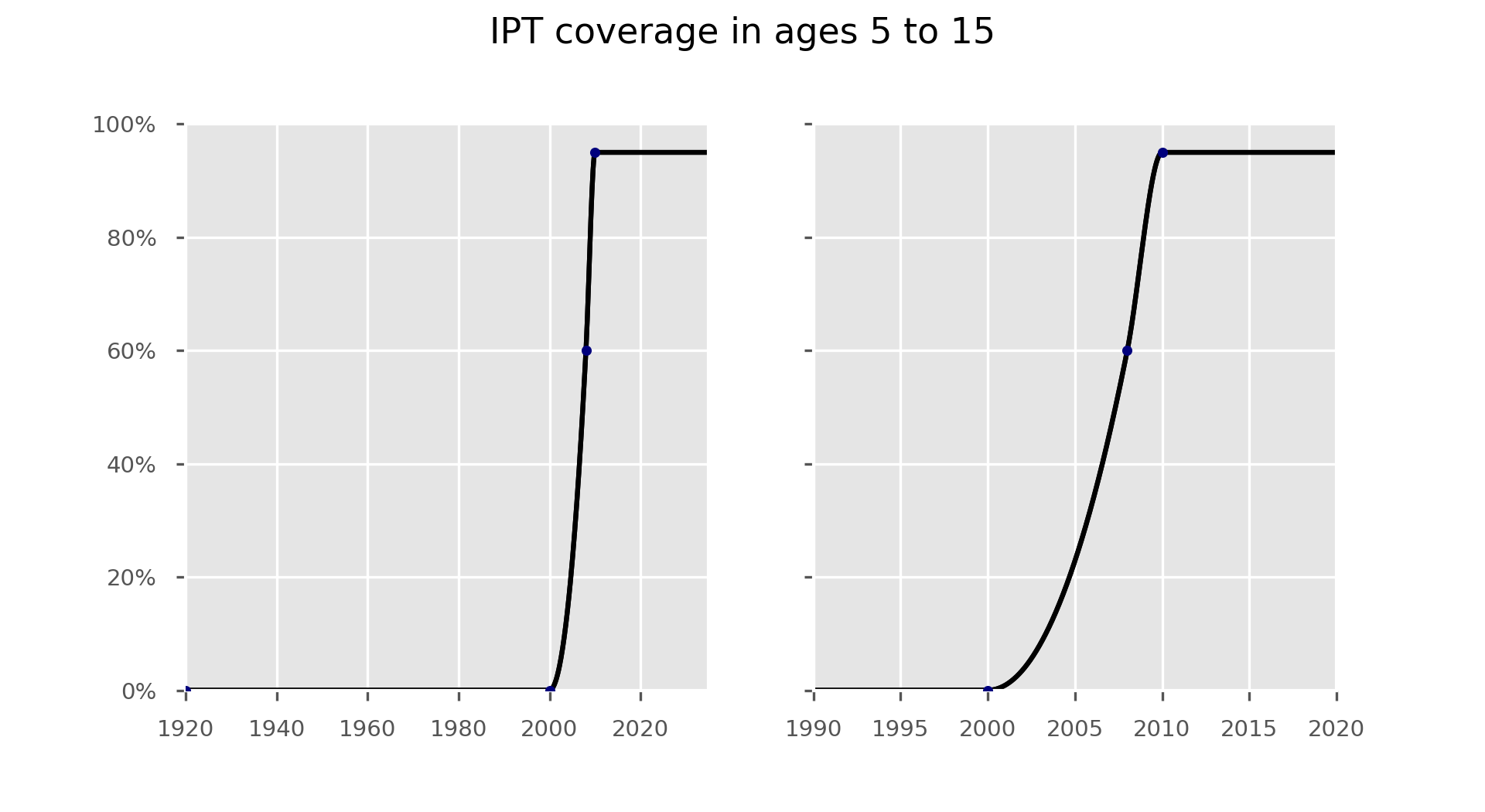


#### **Figure S17. Coverage of isoniazid preventive therapy in ages 15 and under.** That is, applied to both the under 5 age group and the 5 to 15 age group.


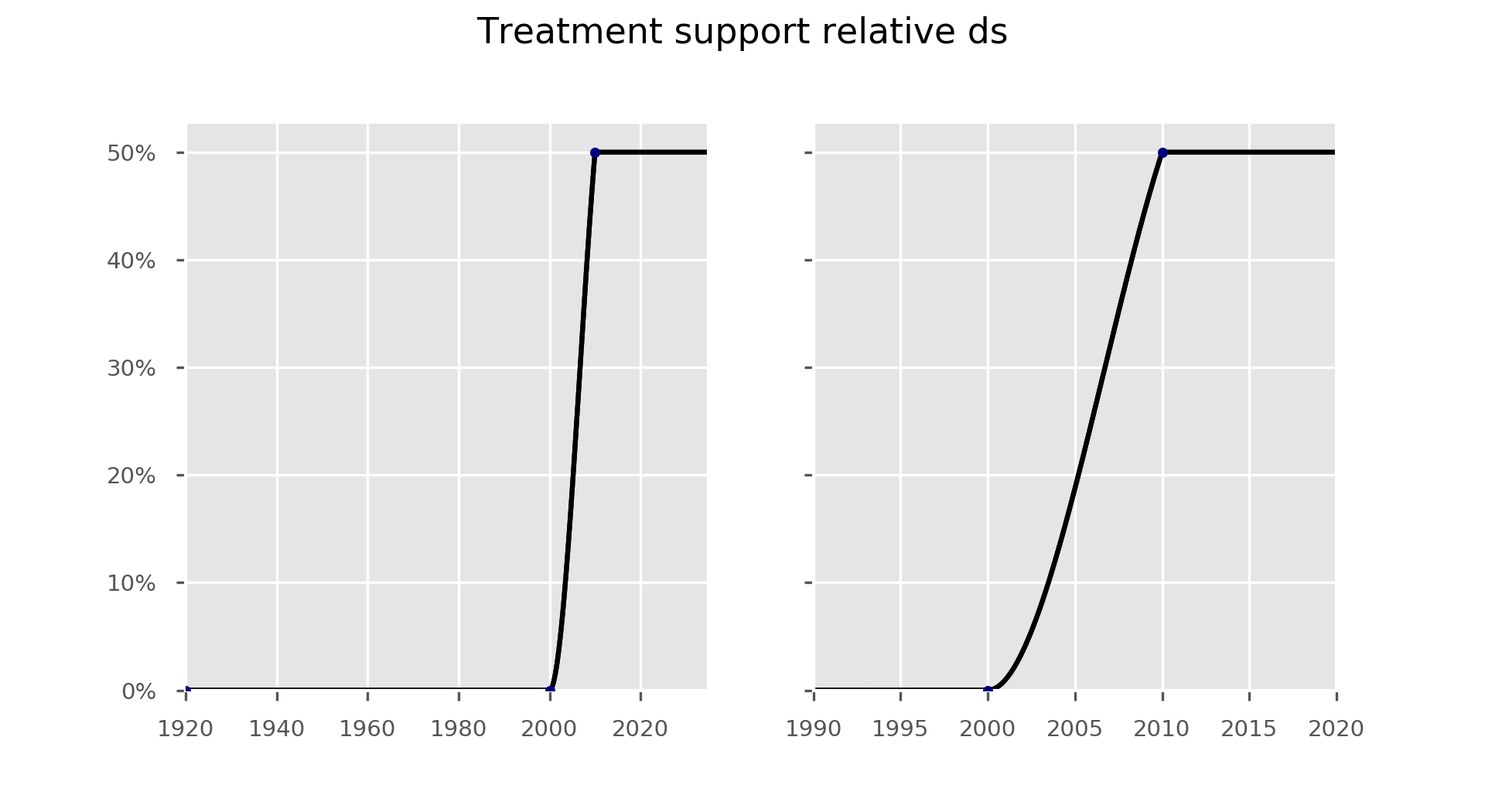


#### **Figure S18. Baseline coverage of treatment support intervention for DS-TB.**


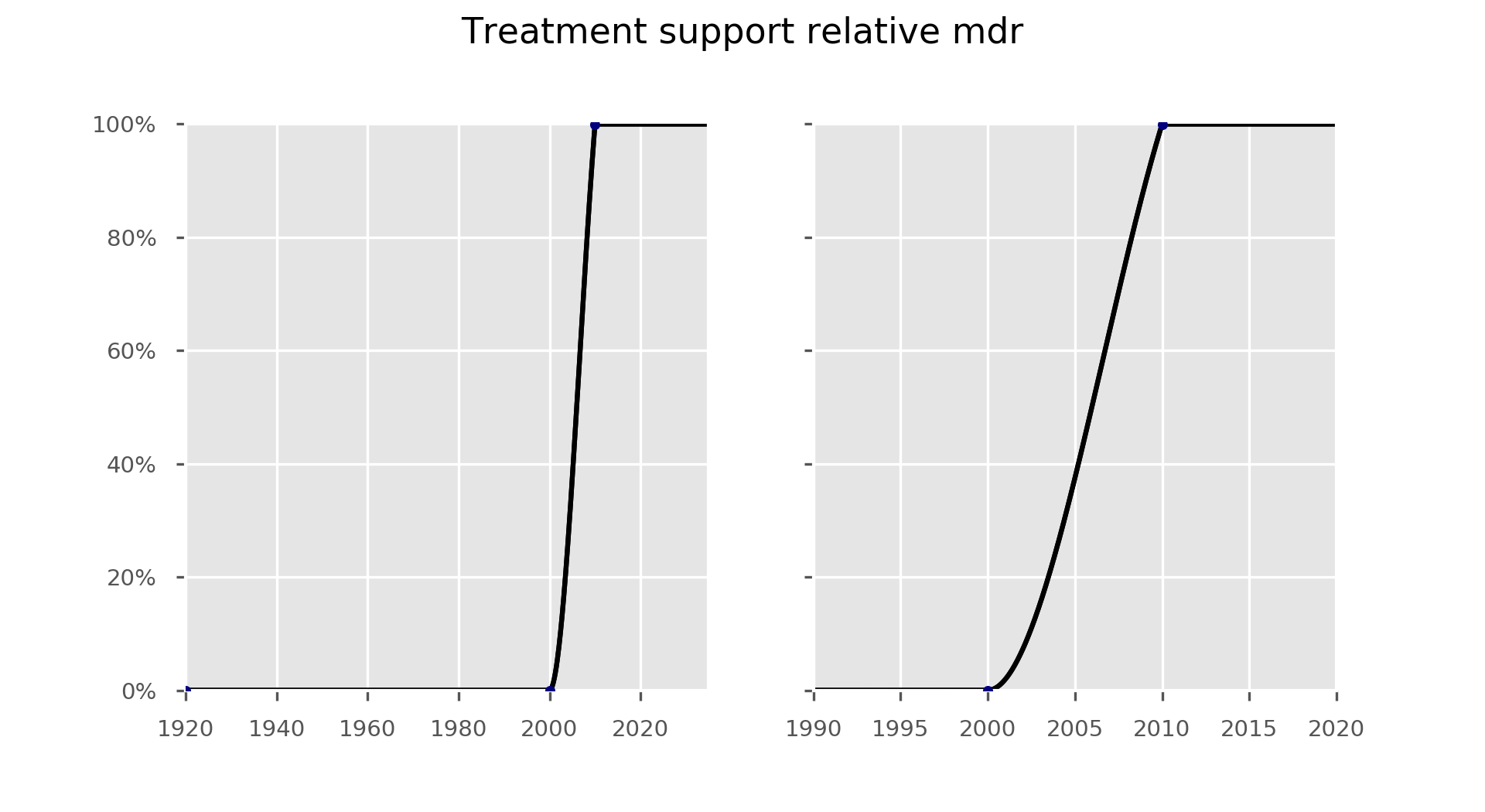


#### **Figure S19. Baseline coverage of treatment support intervention for MDR-TB.**

# Constant parameters

The constant parameters that do not vary with calendar time of model integration are presented in Table S1 below. The rationale and review of relevant evidence used to determine these parameter values are presented in the following sections (Section 7 and beyond).

#### **Table S1. Input values of constant parameters**

| Parameter | Value | Unit |
| --- | --- | --- |
| Latency progression parameters |  |  |
| Duration in early latency (all age groups) | 60 | Days |
| Proportion of persons progressing to active TB from early latency if aged under 5 years | 56 | % |
| Proportion of persons progressing to active TB from early latency if aged 5 to 15 years | 26 | % |
| Proportion of persons progressing to active TB from early latency if aged 15 and above (i.e. both the 15-to-25 and the 25-and-up age groups, in the absence of comorbidities) | 3 | % |
| Rate of progression from late latency to active disease (all age groups) | 0.245 | % per year |
| Natural history of disease |  |  |
| Duration of active disease if untreated | 3 (2 to 4)* | Years |
| Case fatality of untreated smear-positive active TB | 70 (35 to 85)* | % |
| Relative case fatality for smear-negative and extrapulmonary TB, by comparison to smear-positive TB (e.g. 20% if smear-positive CFR is 70%) | 2 ÷ 7 | Multiplier |
| Other biological |  |  |
| Effective contact rate | 6 (5 to 20)* | Contacts per year |
| Proportion of incident cases smear-positive | 33.1 | % |
| Proportion of incident cases smear-negative | 39.4 | % |
| Proportion of incident cases extrapulmonary | 28.5 | % |
| Relative protection from further infection (i.e. multiplier applied to the force of infection) conferred by current latent infection | 0.21 | Multiplier |
| Relative protection from infection for vaccinated persons whose immunity has not waned | 0.5 | Multiplier |
| Age after which protection against infection conferred by BCG vaccination wanes (i.e. BCG vaccination protects youngest two age groups only) | 15 | Years |
| Relative infectiousness of smear-negative cases (by comparison to smear-positive) | 24 | % |
| Relative infectiousness (i.e. contribution to the force of infection) for children aged 10 and below (applied directly to the under 5 age group) | 0.1 | Multiplier |
| Relative infectiousness of children aged 5 to 15 (calculated as mid-point of infectiousness of those under and over 10 years of age) | 0.55 | Multiplier |
| Relative susceptibility to reinfection for previously treated patients compared to fully susceptible | 1 (0.25 to 2)* | Multiplier |
| Risk group-related |  |  |
| Age from which diabetes is applied (i.e. applied only to the oldest of the four simulated age groups) | 25 | Years |
| Odds ratio applied to proportion of persons progressing early to active TB if diabetic | 3.11 | Multiplier |
| Relative risk of progression to active TB from late latency if diabetic | 3.11 | Multiplier |
| Relative infectiousness in prison population | 13 | Multiplier |
| Relative infectiousness in Roma communities | 6 | Multiplier |
| Programmatic |  |  |
| Relative sensitivity of the baseline diagnostic algorithm for smear-negative and extrapulmonary cases | 0.61 | Multiplier |
| Treatment duration, drug-susceptible TB | 6 | Months |
| Duration infectious after commencing treatment, drug-susceptible TB | 10 | Days |
| Treatment duration, MDR-TB | 24 | Months |
| Duration infectious after commencing treatment, MDR-TB | 1 | Month |
| Time period before patients re-start seeking care after a false-negative diagnosis of TB (the “missed” compartment) | 3 | Months |
| Time from presentation to treatment commencement, smear-positive and extrapulmonary cases | 7 | Days |
| Time from presentation to treatment commencement, smear-negative cases | 30 | Days |
| Other miscellaneous |  |  |
| Model run start time | 1865 (1830 to 1920)* |  |
| Modern population targeted in 2016 | 7,200,000 | Persons |
| Infectious seed of active DS-TB patients (i.e. first calibration run starts with 1,000,000 susceptible and three infectious, with total starting population varied but proportion infectious at commencement remaining constant) | 3 ÷ (3 + 1,000,000) | Proportion |

#### ^*^Epidemiological uncertainty parameters varied during calibration.

# Uncertainty parameters

We restrict the parameters that can be varied to TB-specific parameters that would otherwise remain constant throughout model runs. This behaviour is chosen in order that the uncertainty calculations capture the considerable epidemiological uncertainty in such parameters, whereas programmatic parameters are fitted to the best available data for each time point available. Similarly, intervention-related and economic parameters are held constant throughout the epidemiological uncertainty simulations. Each uncertainty parameter is varied between each simulation using a Metropolis-Hastings algorithm with a Gaussian proposal distribution. When running uncertainty, the base case is run to completion and acceptance is determined based on this simulation only.


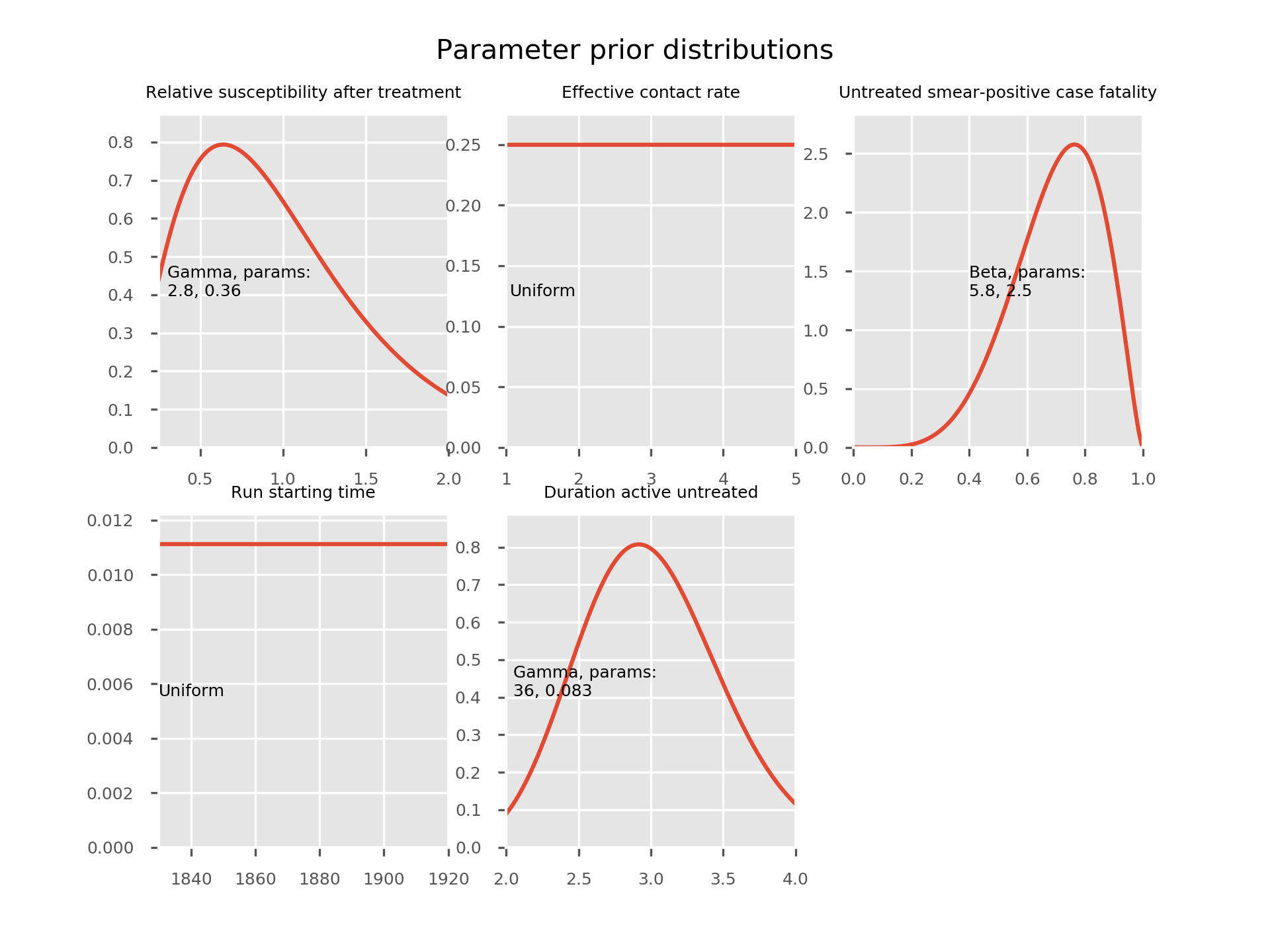


#### **Figure S20. Prior distributions for uncertainty parameters.** Horizontal axis, parameter value; vertical axis, probability density.


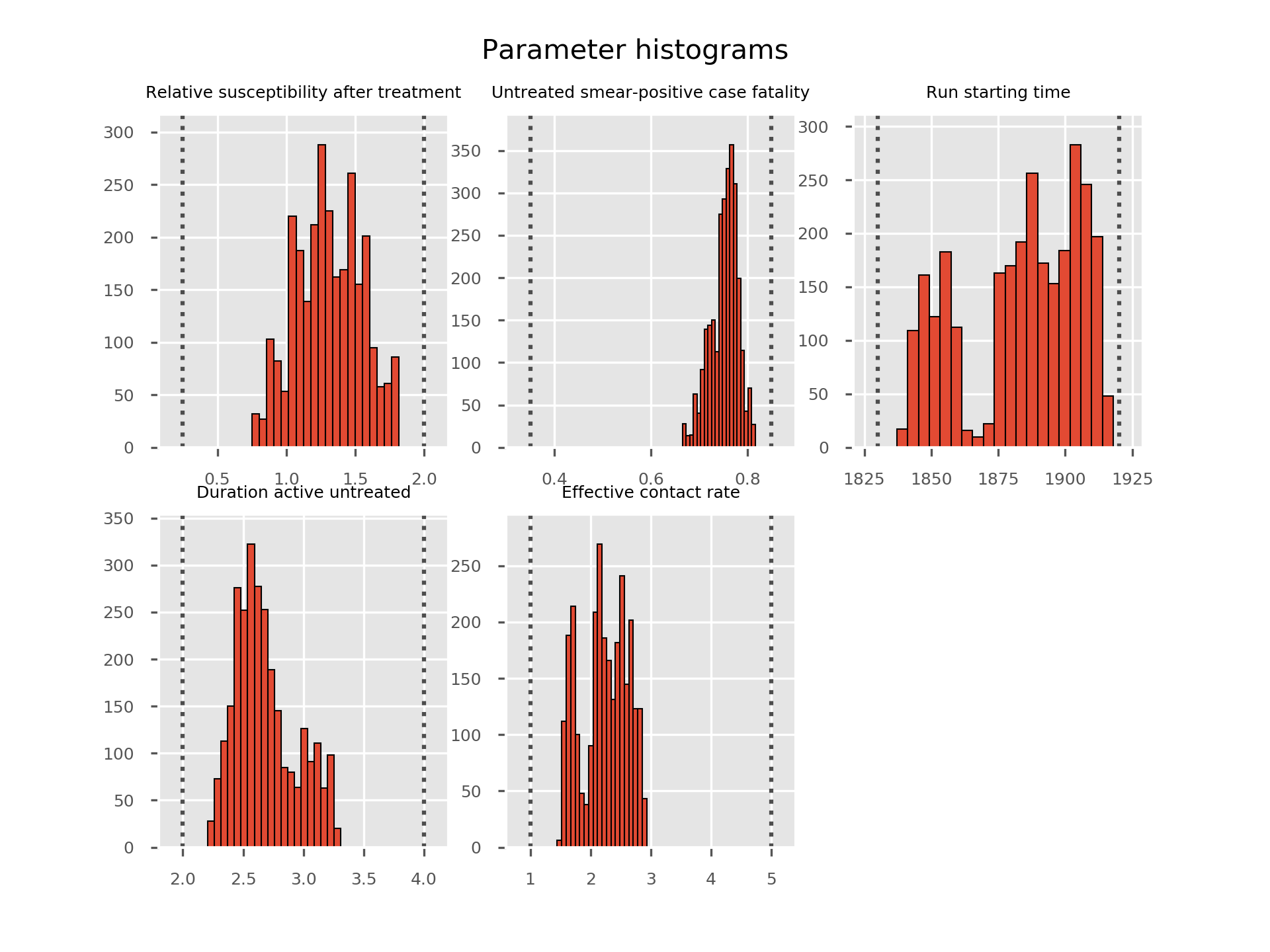


#### **Figure S21. Posterior distribution histograms for uncertainty parameters.** Horizontal axis, parameter value; vertical axis, frequency.

# Initialisation

At model commencement, persons are assigned to the drug-susceptible and active TB compartments only. The proportion of persons assigned to the active compartments remains constant throughout all calibration model runs (Table S1), while the total starting population is algorithmically varied to target a specified modern population (7,200,000). This is achieved by first calculating the ratio of the simulated modern population and targeted modern population at each integration run. At the following run, the starting population is multiplied by the reciprocal of this ratio. For example, if a run over-shoots the modern population by 1%, the starting population is multiplied by 100/101 (decreasing the starting population by approximately 1%). This approach is adopted because under frequency-dependent transmission, the compartment sizes as a proportion of the total population remain the same at any integration time if the starting proportions also remain unchanged.

# Demographic parameters

For simulating births into the model, we assign a time-variant parameter using publicly available data for the crude birth rate from the World Bank.^3^ The birth rate is interpreted as crude births per 1,000 population and so is multiplied by the total population of the model divided by 1,000 to determine the overall birth rate (Figure S1). This is then distributed between vaccinated and unvaccinated births according to Bacillus Calmette-Guérin (BCG) coverage at the time point simulated (Figure S2).

Simulating non-TB-related deaths or the background death rate is achieved with an analogous method. Life expectancy is obtained from publicly available data from the World Bank^4^ and a time-variant parameter is fitted to these data points (Figure S3). Population deaths are then calculated as the reciprocal of this value and applied equally to every model compartment, (including all compartments for patients with active TB, in addition to TB-specific mortality), all risk groups and all age groups.

# Susceptibility/immunity to infection

The extent of susceptibility or immunity to infection among different population groups given their vaccination status, *Mtb* infection-related compartment, age and treatment history is one of the most uncertain parameters in TB epidemiology. We consider homogeneous susceptibility/immunity to *Mtb* infection across risk groups and age groups, assuming that the differing rates of TB disease in these groups are related to other factors (explained further below). By contrast, BCG vaccination and latent infection with *Mtb* are considered to influence the likelihood of infection after exposure to an infectious individual (Table S1). The effect of prior latent infection on reinfection is estimated from a review of cohort studies comparing those with evidence of prior *Mtb* infection to those without such evidence among persons exposed to infectious TB.^5^ This study found that the rate of new episodes of TB disease in those with prior latent infection was 0.21-fold that in those without. Because reinfection should be a minor consideration given the design of this study and because unrecognised reactivation of past infection would lead to even lower values, we use this estimate as our parameter value for the relative rate of reinfection among currently latently infected persons (by comparison to the rate of infection in fully susceptible persons). Note that this only applies to the late latent compartment, as reinfection of those in the early latent compartment will not affect their risk of progression to active disease, as these persons are already at the highest risk of progression to TB.

Subsequent episodes of TB disease after recovery from a prior episode are known to occur at a higher rate than that in the background population.^6^ However, whether this is due to increased host susceptibility to reinfection incurred by the previous episode or to underlying heterogeneity in rates of reinfection at the population level is unclear. In the latter case, it is feasible that recovered or treated individuals have equivalent or even decreased susceptibility to future infection compared to those who have never been infected.^7^ We use a multiplier parameter to allow for individuals who have previously been treated for active TB to be assigned a different level of susceptibility to subsequent infection from those who have not. This multiplier is applied in conjunction with the parameters described above for latent infection and vaccination, such that the level of susceptibility for compartments for which both these conditions are true is the product of these two parameters. For example, previously treated and latently infected persons would have a relative risk of 0.21 compared to fully susceptible persons if this parameter were set to one. This multiplier parameter is varied during the epidemiological uncertainty process (second panels of Figures S20 and S21).

# Model of *M. tuberculosis* infection and progression

### Progression from infection to disease

Our approach to quantifying rates of progression from latency to active disease is described in detail in two published journal articles. The first uses TB surveillance data from a very low transmission setting to estimate rates of active disease following a defined index exposure to an infectious case and evidence of exposure.^8^ The second article uses the reactivation profiles from this study and from a similar epidemiological investigation^9^ to determine the ideal compartmental structure and progression parameters to accurately capture progression to active TB following infection.^10^ This latter publication found that two latency compartments placed in series are highly effective at reproducing epidemiological observations when appropriately parameterised and provides appropriate parameter values. Specifically, following infection, all latently infected individuals first progress to a high-risk “early latent” compartment, from which they may progress rapidly (within months) to active TB. Alternatively, these individuals may “stabilise”, transitioning to a low-risk “late latent” compartment, from which progression to active TB occurs much more slowly. This configuration is also consistent with that used or recommended in other important TB modelling papers.^11,12^ This approach requires estimation of three epidemiological parameters for each age group: the time period spent in the early latent compartment, the proportion of individuals progressing to active disease from the early latent compartment and the rate of progression from the late latent compartment to active disease (presented in Table S1). These are then converted into three transition rates for early progression, stabilisation and late progression. That is, the late progression rate requires no further processing, while the early progression and stabilisation rates are calculated as:

$$early progression rate= \frac{early progression proportion}{time in early latency}$$

$$stabilisation rate= \frac{1-early progression proportion}{time in early latency}$$

### Organ manifestations

We distinguish three types of “organ manifestation” of active TB: smear-positive pulmonary, smear-negative pulmonary cases and extrapulmonary cases. We assume that the proportions of incident TB cases entering each of these three categories are constant over time. We calculate these values as the total number of notifications reported by each organ manifestation and use this constant value for the proportion of incident cases by organ manifestation. These data are available for multiple time points and AuTuMN has functionality that allows the proportion of incident cases reaching each of these states to be time-variant. However, because these data reflect the proportion of notifications rather than incident cases by organ manifestation, we consider that an increase in notifications of a particular category may actually reflect improved detection of this form of TB (and vice versa). Therefore, increasing the proportion of patients with a particular organ manifestation status by increasing the rate of incidence for this category may be the opposite of the epidemiologic reality, because the increased rate of detection for this category of patient would have decreased the proportion with this manifestation.

### Natural history

Once TB disease has manifested, natural history parameters are first set to reflect outcomes in the absence of detection by the health care system. The total rates of progression to either death or spontaneous recovery are set equal to the reciprocal of the time period of untreated active disease. Next, a proportion of this total rate is then allocated to each of these two possible outcomes (death and spontaneous recovery), according to the case fatality rate for smear-positive TB, which is a calibration parameter. The smear-negative and extrapulmonary case fatality rates are then both set to be proportionately lower than the smear-positive case fatality rate by multiplying by the fixed relative case fatality parameter, such that these quantities vary with the case fatality rate for smear-positive TB. Parameter values and uncertainty ranges are estimated from a detailed published review of pre-chemotherapy era outcomes.^13^

# Case detection

### Obtaining the case detection ratio

The case detection rate is a time-variant parameter and is a fundamental parameter determining the performance of TB programs at the country level. Estimates of the case detection rate are provided by the World Health Organization in the Global TB Report.^14^ It is important to note that this “rate” is actually a ratio or proportion and is referred to as the case detection ratio hereafter. Specifically, the case detection ratio is the proportion of all active cases that are detected during their disease episode, closely related to the ratio of notifications to incidence in the region considered. Variations in its magnitude have major impacts on disease burden that may only be realised decades later. Because effective medical treatments for active TB only became available from the 1950s onwards, we apply an effective case detection ratio of zero prior to that time. As with other time-variant parameters, case detection ratio data are loaded from the Global TB Report data. In the case of Bulgaria, we multiply the reported values by 0.95 on advice from the local members of the country teams that the high reported values may be over-estimates.

### Adjustment to the case detection ratio by organ status

We assign different values to the case detection ratio according to organ status because it is likely that smear-positive, smear-negative and extrapulmonary cases are diagnosed by the health care system at different rates. Smear-positive cases may be targeted for detection and may be easier to diagnose with locally available tools, in particular smear microscopy. Moreover, several interventions may act to close this diagnostic gap between smear-positive and smear-negative or extrapulmonary cases. For example, availability of phenotypic culture testing improves the possibility of detection with smear-negative TB cases relative to smear microscopy and clinical judgement, partially closing this detection gap.

In our model, the rate of detection for smear-positive cases is increased, while the rates of detection for smear-negative and extrapulmonary cases are decreased. This is calculated such that smear-negative and extrapulmonary cases have a case detection ratio that is lower than smear-positive cases by a constant specified ratio, while the weighted average case detection ratio for all forms of TB remains equal to the unprocessed case detection ratio originally estimated.

### Missed diagnosis and the accuracy of the diagnostic algorithm

In order to explicitly simulate patients who presented for care but whose diagnosis was missed, a flow rate that transitions patients from the active untreated compartment to a missed diagnosis compartment is simulated. Patients whose diagnosis has been missed suffer outcomes applicable to those in the active treatment compartment in terms of death and spontaneous recovery, but do not re-start seeking treatment for a limited period of time. To achieve this, it is considered that case detection is proportional to the diagnostic algorithm’s sensitivity, as missed cases are proportional to its complement. That is:

$$rate of case detection\times algorithm accuracy= rate of missed diagnosis\times\left( 1-algorithm accuracy \right)$$

The adjustment process by organ status described for the rate of case detection is applied in the same way to the sensitivity of the baseline diagnostic algorithm parameter, in order to simulate a greater proportion of smear-positive persons being detected rather than missed under baseline conditions through clinical assessment and smear microscopy.

# Treatment outcomes

### Determining aggregate outcomes

Treatment outcomes are derived from the Global TB Report, which reports outcomes for patients differently by strain, HIV status, sputum smear status and treatment history, as well as changing its reporting approach for some categories after 2011. All reported treatment outcomes are grouped into three broad categories for model interpretation: 1) treatment success, 2) death during treatment and 3) survival with unfavourable outcomes (i.e. any outcome other than success or death, the terminology for these pathways having changed over time as follows).

Up to and including 2011, patients achieving treatment completion or cure (the latter applicable to smear-positive patients only) are classified as treatment success, patients dying on treatment are classified as such and patients reported as default or failure are classified into the third category. Up to 2011, relapse cases are included with retreatment cases in Global TB Report data. After 2011 for non-MDR-TB patients, loss to follow-up and failure are classified as unfavourable outcomes (as opposed to default and failure up to 2011). After 2011, relapse cases are included with new cases in official data, by contrast to the reverse situation up to 2011. Because there is no reliable way to account for this change, it should be noted that outcome parameters are fit to data that changes in interpretation after 2011, although this change is very minor numerically.

### Calculation of proportions

Once the total outcomes have been classified into the three categories described above, the proportion of patients falling into each category is calculated. Two time-variant proportion functions are then defined. The first is the treatment success proportion, for which the numerator is successful treatment outcomes (cure and completion) and the denominator is all treatment outcomes (Figures S7, S9, S11 and S13). The second is the proportion of non-success outcomes resulting in death, for which the numerator is death outcomes and the denominator is all treatment outcomes other than success (Figures S8, S10, S12 and S14).

Time-variant functions representing proportions (and therefore limited to a range of zero to one) are then calculated for these two quantities. The proportion of treatment outcomes resulting in death is then calculated as the proportion of non-success outcomes multiplied by the proportion of unsuccessful outcomes resulting in death. The proportion of treatment outcomes that are unfavourable other than death is the reciprocal of these two quantities. Using this approach, all treatment outcomes must consistently remain non-negative and the three possible outcomes always sum to one.

# Treatment history

All compartments are duplicated according to whether patients have been previously treated or not. The only exception is for the fully susceptible unvaccinated compartments, which are not duplicated because there is no equivalent compartment representing fully susceptible individuals in the previously treated stratum. This is because previously treated individuals are assigned a modified level of immunity/susceptibility to future episodes of infection. The treatment outcome parameters also differ for DS-TB patients under treatment who have been previously treated, as shown in Figures S9 and S10. Although treatment outcomes are likely to differ for re-treatment MDR-TB patients by comparison to new MDR-TB patients, the same treatment outcomes are used for the whole MDR-TB cohort in Bulgaria because the cohort size is too small to estimate time-specific outcomes for each group separately.

# Age differences

### Risk of progression to active disease

The natural history of childhood TB was described in detail by Marais *et al*. in their 2004 publication that considered historical publications on this topic in detail.^15^ The period from 1920 to 1950 was particularly interesting for the study of the natural history of TB, as chest radiography had become available but effective treatment to modify the natural history had not. Although quantitative estimates of the risk of progression to active disease and subtypes are presented, we prefer the publications by Sloot *et al.* and Trauer *et al.*, as these two modern estimates employ formal survival analysis techniques and are highly consistent with one another.^8,9^ Although widespread use of preventive therapy is a consideration in interpretation, this is offset by reinfection being a minor consideration.

### Infectiousness

Although children are often considered non-infectious, some groups of children are likely to have the potential to transmit infection, including adolescents who more often have adult-type reactivation TB. Other potentially infectious paediatric groups include those with pulmonary cavitation, positive smears for acid fast bacilli (AFBs), laryngeal involvement, widespread pulmonary disease and suspected congenital TB (which tends to be more extensive). However, younger children, including those with primary pulmonary TB are unlikely to be infectious because of their low bacillary load^16^ and inability to generate a sufficiently forceful cough.^17^ This is supported by the observation that an infectious adult was consistently identifiable in outbreaks of TB in orphanages and children’s hospitals, while such outbreaks did not occur when only a child was identified with active TB. Eight case reports of transmission from children were identified in a 2001 review, which included four children aged under one (including two with congenital TB), with the others aged 3, 5, 7 and 9. However, the extent of transmission was either limited or incompletely described in most of the six reports considering children aged five and below. Unpublished data recording low rates of tuberculin skin test (TST) conversion in staff of paediatric hospitals also support this contention.^18^ For these reasons, we reduce the infectiousness of children by one order of magnitude (i.e. 0.1 times that of adults) and apply this estimate to the under-5 age group. For the age group aged 5 to 15, we assume that approximately half of these patients will have adult-type and half child-type manifestations of disease, resulting in a weighted average of 0.55 times the infectiousness of adults for this group.

### Case fatality

The case fatality rate (a proportion) for children with miliary TB was estimated at 14% in one case series,^19^ which is considerably greater than the estimate of 3.5% for all cases from one meta-analysis.^20^ However, this is clearly not a direct comparison, as this higher rate was observed only in a subgroup of paediatric cases and may well be offset by lower case fatality rates in other subgroups, such as tuberculous lymphadenitis. Moreover, these estimates primarily consider patients under treatment and so cannot be used to estimate the case fatality of untreated TB (which is of greater relevance to our model parameters). For these reasons, we apply the same natural history outcomes to children as to adults.

### Summary of model implementation

We use the age-specific latency progression proportions reported by Ragonnet *et al.* and are currently undertaking further research to refine these parameters further. Although difficult to quantify, the infectiousness of persons aged under 10 is lower than those of older ages, and we apply a ten-fold reduction as described above. Case fatality rates are not currently modified for age, although we continue to seek evidence to quantify this.

# Diabetes

We estimate the relative risk or odds ratio for developing TB in patients with diabetes by comparing the risk of disease in affected patients to the risk for patients without diabetes. Ideally, a relative risk or hazard would be applied to the rate of late progression from late latency to active disease, while an odds ratio would be applied to the proportion of patients in early latency who progress to active disease rather than stabilising to late latency. However, we also consider it desirable for these two quantities to be mutually consistent.

For individuals in early latency, we do not adjust the duration of early latency for persons with comorbidities, but only the proportion of patients undergoing early progression to active TB. We apply an estimate of the odds ratio for progression to active disease associated with the comorbidity to the original estimate for the proportion progressing, which is age-specific in age stratified models. We term the proportion of persons in early latency progressing to active TB in the absence of comorbidities “*p_n_*”, such that the odds of progressing are equal to *p_n_ / (1 – p_n_)*. Similarly, the proportion progressing in the presence of the comorbidity is termed “*p_c_*”, with associated odds *p_c_ / (1 – p_c_)*. Therefore, if the odds ratio associated with the comorbidity is termed “OR”:

$$\frac{p_{c}}{1- p_{c}}=OR\times\frac{p_{n}}{1- p_{n}}$$

Solving for p_c_ gives:

$$p_{c}=\frac{p_{n}\times OR}{p_{n}\times\left( OR-1 \right)+1}$$

This formula is used to calculate the rate of progression in the presence of diabetes. It has the advantages of producing a similar modification in *p_c_* to that achieved with a relative risk calculation if OR is relatively small, but having a ceiling value at one for large ORs. As this is a proportion that is used to determine the rates of flows from early latency, the rate of progression from early latency to active disease and to late latency are:

$$early latency to active disease progression rate=\frac{p_{c}}{duration early latency}$$

$$early to late latency progression rate=\frac{{1 - p}_{c}}{duration early latency}$$

Several epidemiological studies have found a greater rate of incident TB in patients with diabetes across a range of contexts.^21,22^ Observations from such individual studies include a generally similar or greater rate of bacteriologically-confirmed disease than for all forms^23^ and similar increases in risk associated with recent and reactivation disease.^24^ One study reported a slightly lower strength of association, but recruited controls with medical conditions that are potentially weakly associated with TB.^25^ A limitation of several of these studies is that TB itself is known to be associated with transient impairment to glucose metabolism,^26,27^ although the above studies varied as to whether they considered previously diagnosed diabetes or performed glucose metabolism testing at the time of diagnosis. Several of the abovementioned studies were included in a meta-analysis of the association of diabetes on TB published by Jeon *et al.* in 2008.^28^ This study found a relative risk for diabetes of 3.11 (95% CI 2.27 to 4.26) from a random effects meta-analysis of three cohort studies. To apply the modification in the rates of progression to active disease due to diabetes, we adopt the approach outlined above, estimating both the odds ratio for early progression and the relative risk for late progression at 3.11.

# Prison and Roma populations

The prison population and Roma communities are two groups that are known to have higher rates of TB disease than the general population in Bulgaria and are considered key groups for control. It is estimated that there are around 700,000 Roma people, accounting for 10% of the total population. The Roma community is characterised by their high poverty rate (84% versus 7.6% in the general population) and high unemployment rate (80% versus 10% in the general population). TB burden among Roma people varies among regions; however nationwide it is estimated that they account for around 30% of all reported TB cases in the country. About 50% of MDR-TB patients are Roma people. Low treatment adherence, poor treatment outcomes, the size of the population (up to 10% of the total population), and possibly low detection rate of TB cases, make Roma people a high priority group for TB control. Stigma towards TB is one of the biggest barriers to communication and conversation in the community, and for seeking health services by Roma people.^29^

The prison population is currently around 7,921 people or 1% of the population. This group is thought to have an approximately thirteenfold (300 per 100,000 per year) greater burden of TB than the general population, and accounts for 1.5% of all TB cases reported in Bulgaria.

We simulate higher rates of disease in these two groups by increasing their contribution to the force of infection (multiplier parameters presented in Table S1) and introducing heterogeneous mixing. This is intended to reflect living conditions that increase the effective contact rates among these groups and we consider this approach more likely to reflect reality than alternative options, such as increasing progression rates or susceptibility to disease. As this increased contribution to the force of infection would not result in increased disease rates in these sub-populations alone, a mixing matrix is required to allow for assortative mixing between these groups (Figure S22). The parameters to the mixing matrix were developed on advice from local experts, while the multiplier parameters were calibrated to the relative incidence of TB in these groups and for consistency with a previous AuTuMN application (the Philippines).


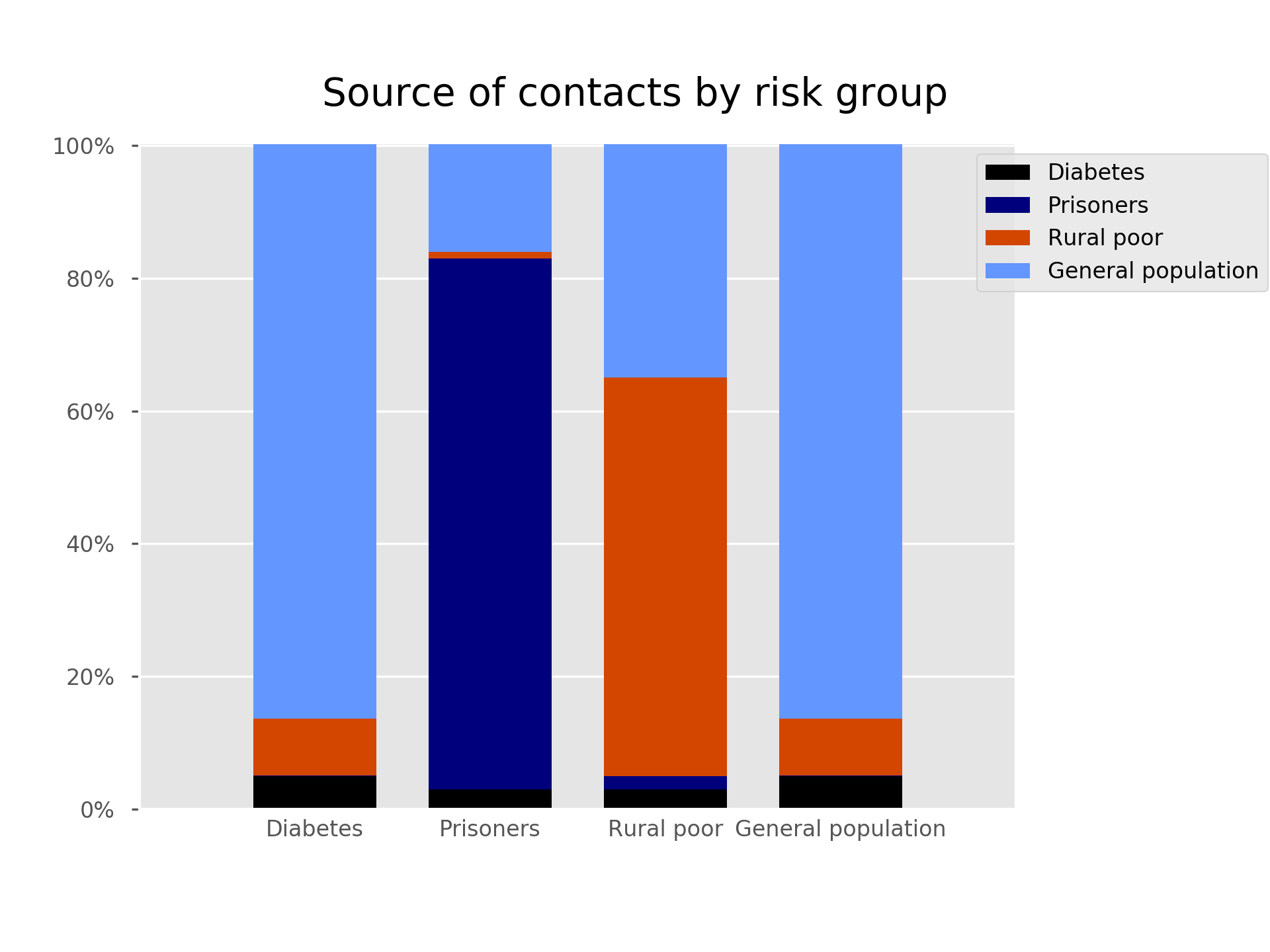


#### **Figure S22. Mixing matrix.** Columns represent the population at risk of infection, while the height of the coloured bars show the proportion of contacts received from each sub-group. For example, prisoners receive 80% of their effective contacts from other prisoners. “Rural poor” indicates Roma communities.

# Cost-coverage logistic function

The logistic cost-coverage function takes the following form:

$$cov\left( cost \right)=A+ \frac{sat-A}{\left( 1+e^{-B.cost)} \right)^{\alpha}}, (1)$$

where *sat* represents the saturation coverage associated with the intervention (i.e. the maximum coverage possible); *α* is a parameter affecting the shape of the logistic curve (equal to 1 by default); and *A* and *B* are constants that we determine using the conditions described below.

As start-up costs are generally considered separately from this logistic function, the curve is assumed to pass through the origin, which by continuity of the logistic function implies:

$cov\left( 0 \right)=0$. (2)

By combining equations (1) and (2), we obtain:

$A= \frac{sat}{1-2^{\alpha}}$ . (3)

From the assumption of maximal efficiency as soon as spending becomes positive (i.e. from the origin), we obtain:

$\frac{d cov}{d cost}\left( 0 \right)=\frac{1}{u.p}$ , (4)

where $u$ denotes the unit cost and $p$ the size of the population to which the intervention could potentially apply.

Deriving equation (1) and using equation (4), we finally obtain:

$B= \frac{2^{\alpha+1}}{u.p.\alpha.(sat-A)}. (5)$ .

This approach allows cost-coverage curves to be rapidly calculated during model runs from economic input parameters and model properties (i.e. sizes of the target populations).

# Illustration of underlying model dynamics

In this section, we present illustrations of some of the demographic and epidemiological processes underpinning the baseline simulation. Figure S23 indicates the number of individuals in each age group and over time during model integration, illustrating the observed contraction in the total population size over recent years. Figure S24 illustrates the proportion of the population by each of the four risk categories (including no additional risk or the “general population”) over time.


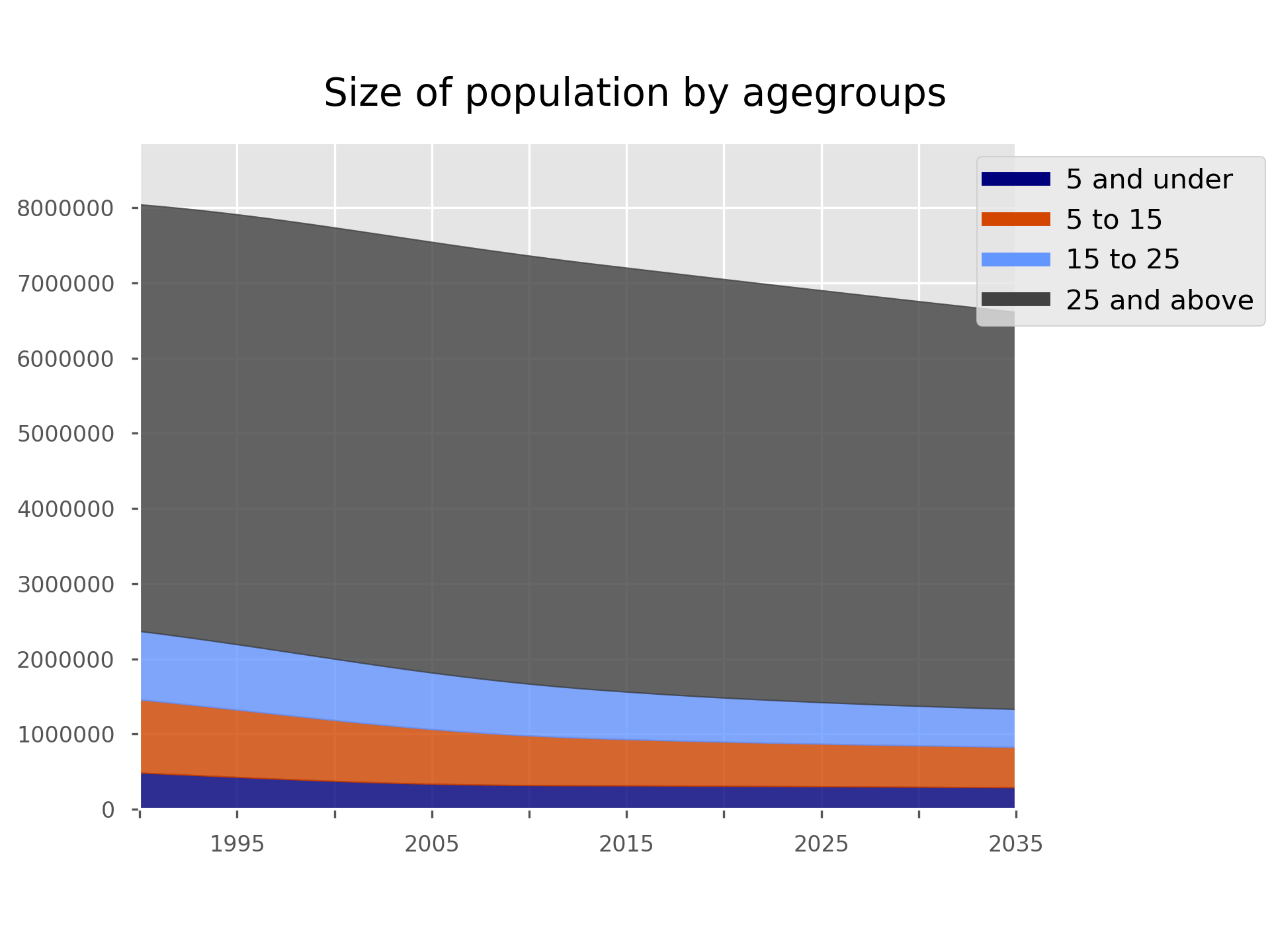


#### **Figure S23. Population sizes by age group and time under baseline scenario.** Legend indicates age of age group in years.


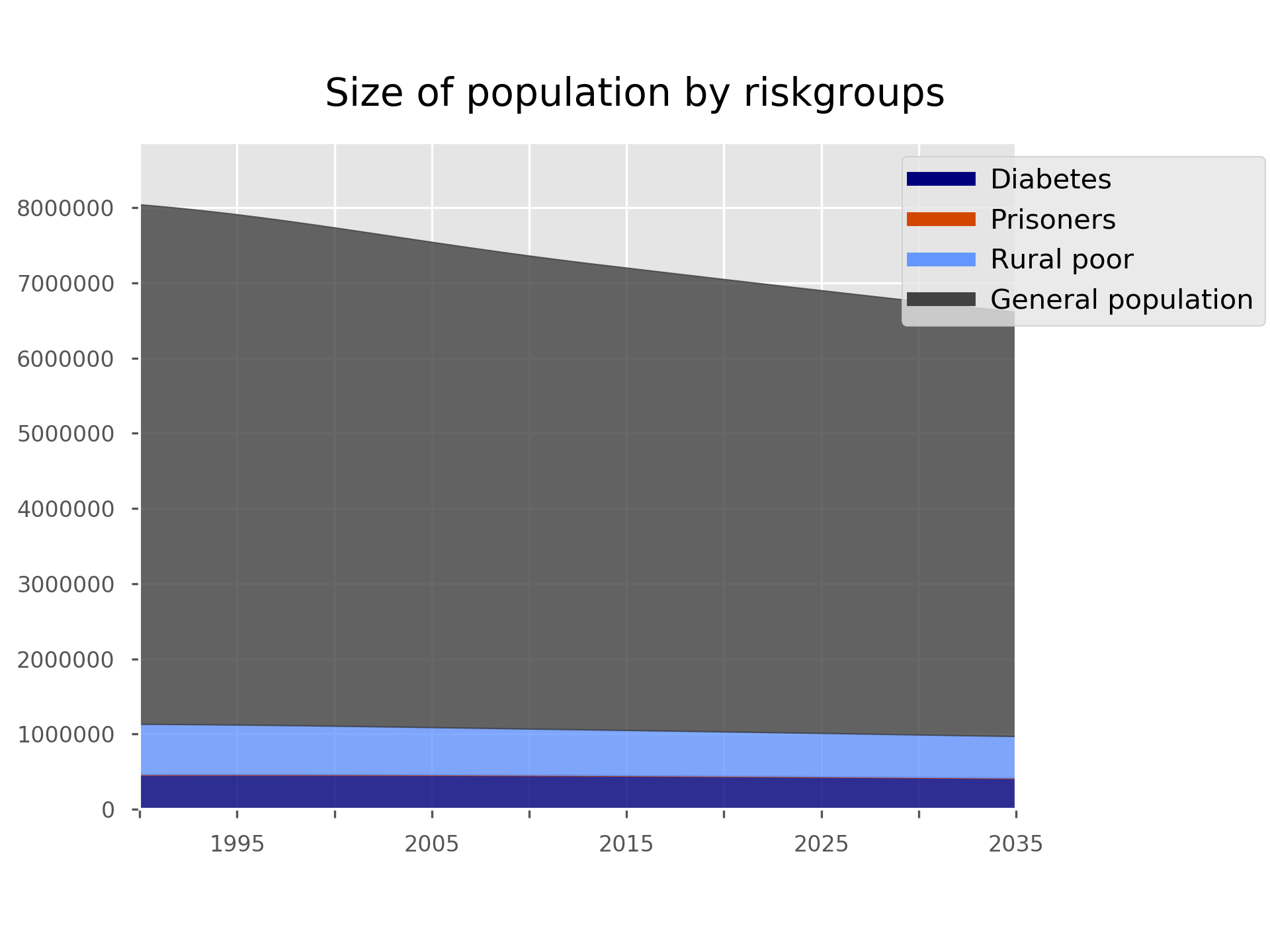


#### **Figure S24. Population sizes by risk group and time under baseline scenario.** “Rural poor” indicates Roma communities.


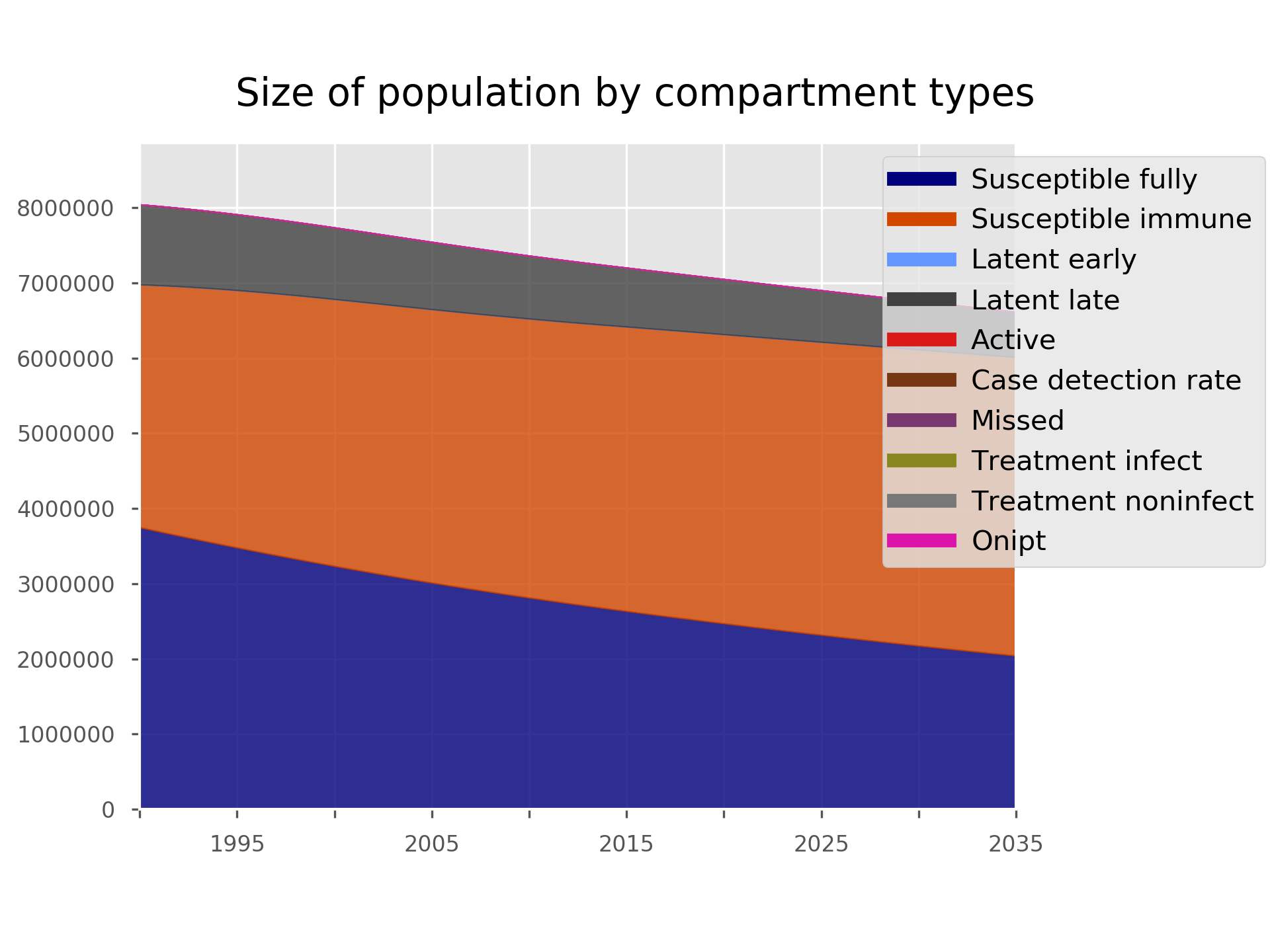


#### **Figure S25. Population sizes by TB compartment and time under baseline scenario.** Only three compartment types are sufficiently large to be visible in this illustration.


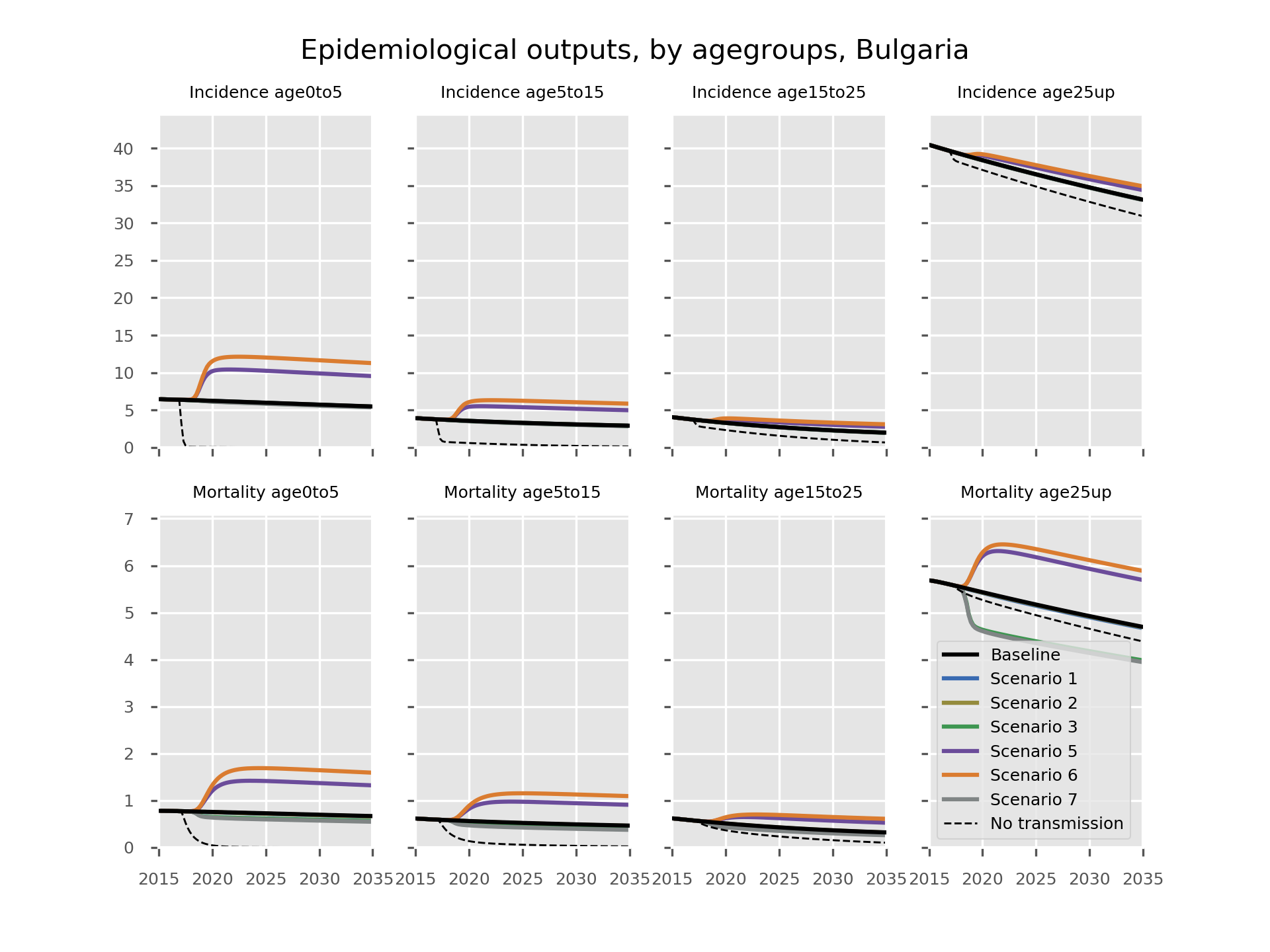


#### **Figure S26. Disease burden (incidence and mortality) by age group.** Scenarios and axis values are as indicated in Figure 3 of the main manuscript.


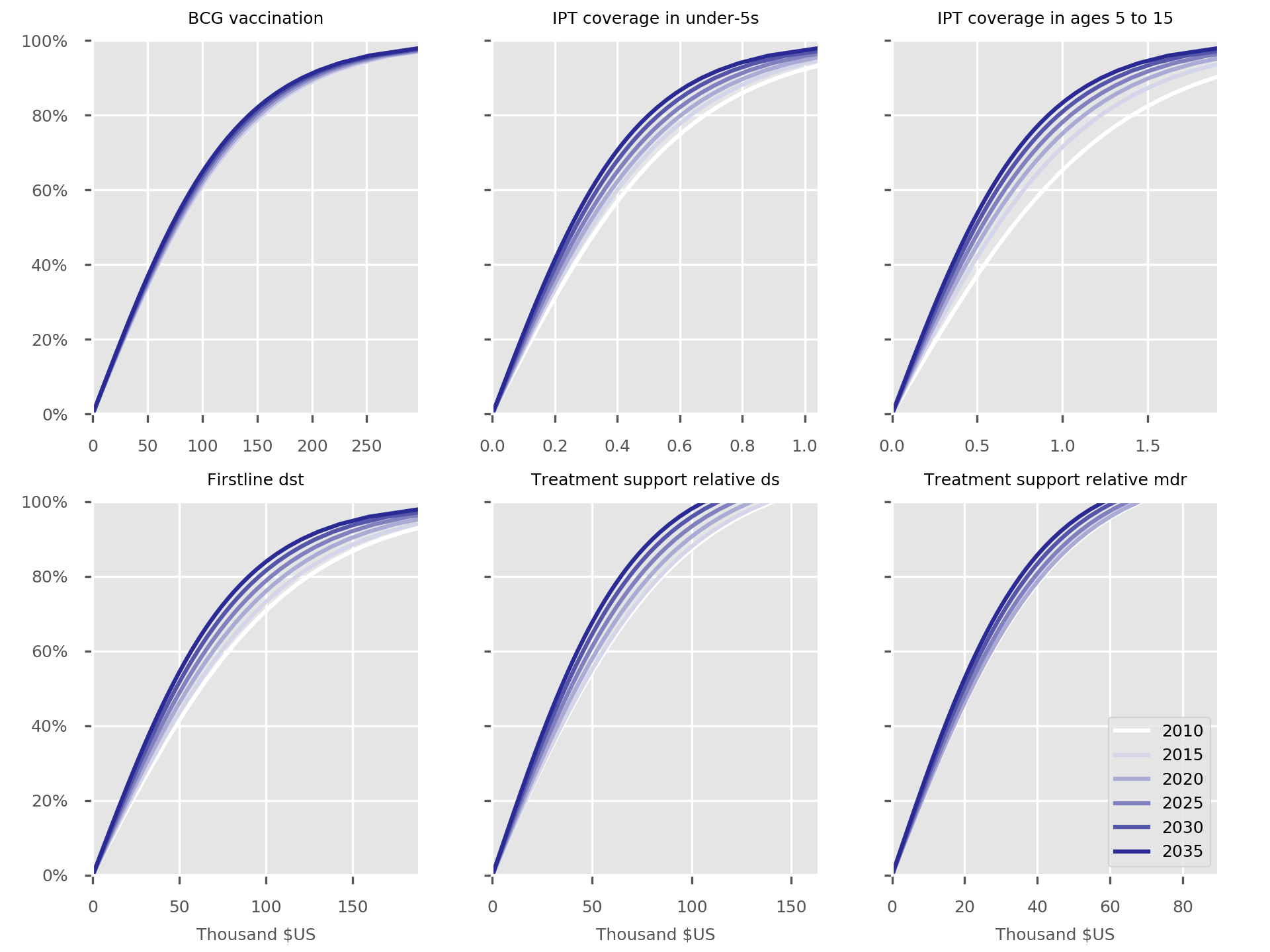


#### **Figure S27. Cost-coverage curves for all interventions implemented under the baseline scenario.**

# Short course regimen for MDR-TB

### Background

Rifampicin-resistant and multidrug-resistant TB (MDR-TB) are critical threats to global tuberculosis control. Less than a quarter of all cases are started on effective second-line treatment and treatment success rates are generally only around 50%, such that around 10% of all incident cases worldwide successfully reach treatment completion. We have undertaken previous modelling (using an earlier iteration of the AuTuMN tool) to demonstrate that short course MDR-TB regimens have the potential to be highly effective in controlling the MDR-TB epidemic, although this is highly dependent on the programmatic background in which they are implemented.^30^

### Evidence

Short course regimens have been proposed and implemented in a number of settings in Africa and Asia and have been associated with relapse-free treatment success rates of around 84 to 90% and lower costs than traditional regimens, although only sub-groups of patients are targeted for treatment.^31–34^ The treatment typically consists of nine to eleven months of a fluoroquinolone, ethambutol, pyrazinamide and clofazimine, which is supplemented by high dose isoniazid, kanamycin and prothionamide in the intensive phase of treatment. WHO has recommended the regimen for patients without extrapulmonary TB, who are not pregnant and for whom all component medications are likely to be effective (based on treatment history and the known or presumed drug susceptibility profile of the infecting organism). However, this evidence is based on observations of cohort of patients treated with such regimens and is currently under evaluation in a randomised controlled trial.

### Implementation

In AuTuMN, implementing the short course regimen is achieved by decreasing the duration of treatment for MDR-TB and by increasing treatment success rates. The treatment success proportion is increased from its original baseline level to a value closer to the expected treatment success proportion, dependent on the coverage of this intervention. The final treatment success proportion for MDR-TB implemented in the model is calculated as:

$$final treatment success=original treatment success+\left( intervention treatment success-original treatment success \right)\times intervention coverage$$

Similarly, the duration for the short course regimen is decreased according to the original MDR-TB treatment duration and the coverage of the intervention:

$$final treatment duration=original treatment duration-\left( original treatment duration-intervention treatment duration \right)\times intervention coverage$$

This calculation is done for both new and re-treatment cases who are on the appropriate regimen for MDR-TB. The calculation of the proportion of unsuccessful outcomes resulting in death and other unfavourable outcomes remains unchanged, such that both the rate of death and other unfavourable outcomes decreases proportionately as treatment success improves. Because the short course regimen is considered to improve treatment outcomes, a floor is set to prevent the final treatment success value being less than the original treatment success value.

Because the number of patients ineligible for the short course regimen in Bulgaria is likely to be higher than in many other settings, the intervention coverage value (for Scenario 1) is set at 50%.^35,36^ For estimating the economic impact of the intervention, the population size is considered to be the number of patients under treatment for MDR-TB (with any regimen).

### Results

Due to the small proportion of cases that are contributed by MDR-TB in Bulgaria, changing to the short course regimen has a negligible effect on overall dynamics in the country (Figure S28). However, the effect on MDR-TB-specific indicators is marked and rapid. The most notable effect is on MDR-TB prevalence, which is partly mediated through the effect of reducing the treatment duration alone (because patients are considered prevalent cases throughout their treatment period). The transient increase in mortality is due to patients reaching their final treatment outcomes more rapidly, which includes death on treatment. This is noted to be a transient change and could well be observed in reality.


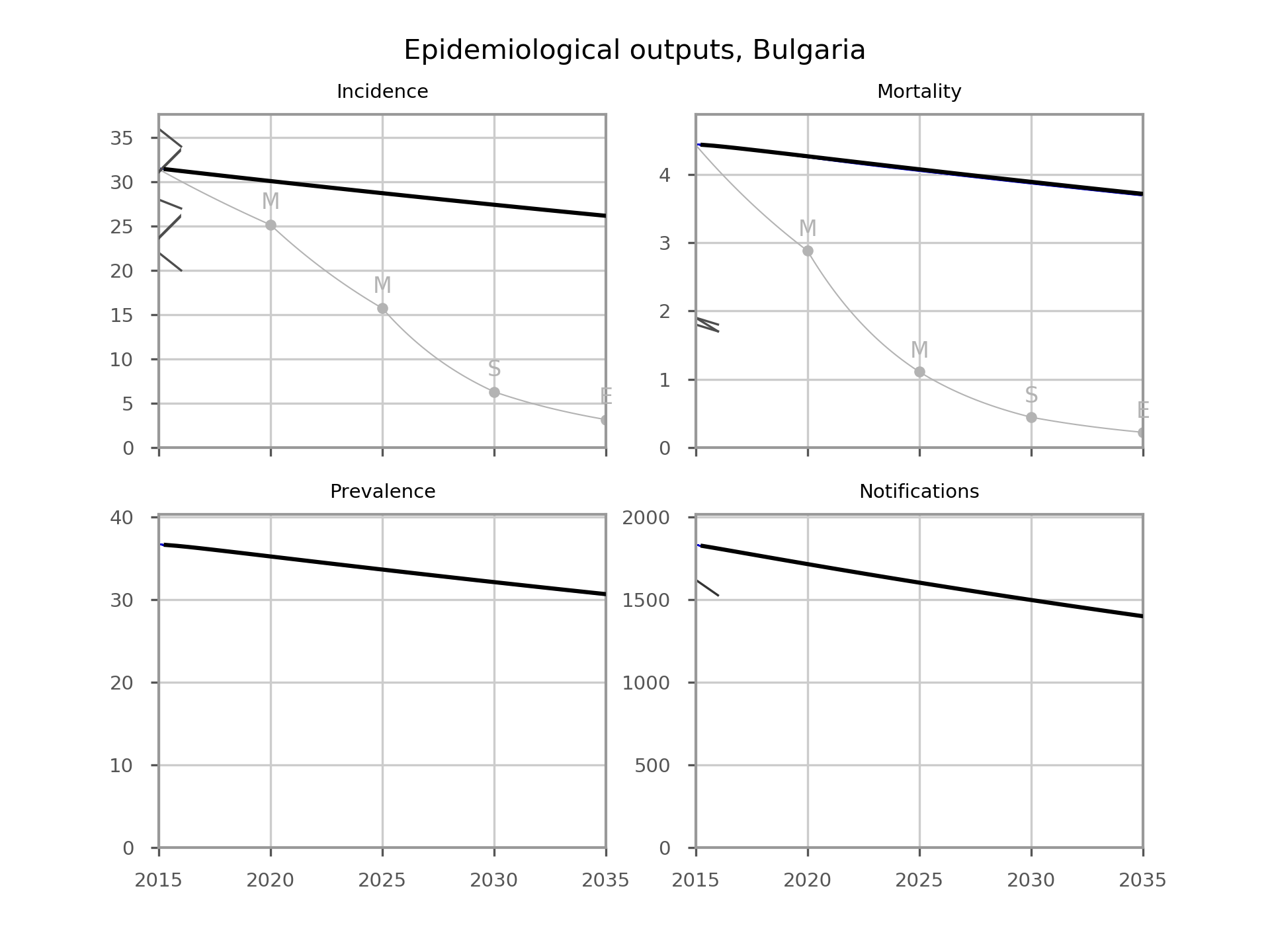


#### **Figure S28. Effect of MDR-TB short course regimen on epidemiological outcomes, with uncertainty.**


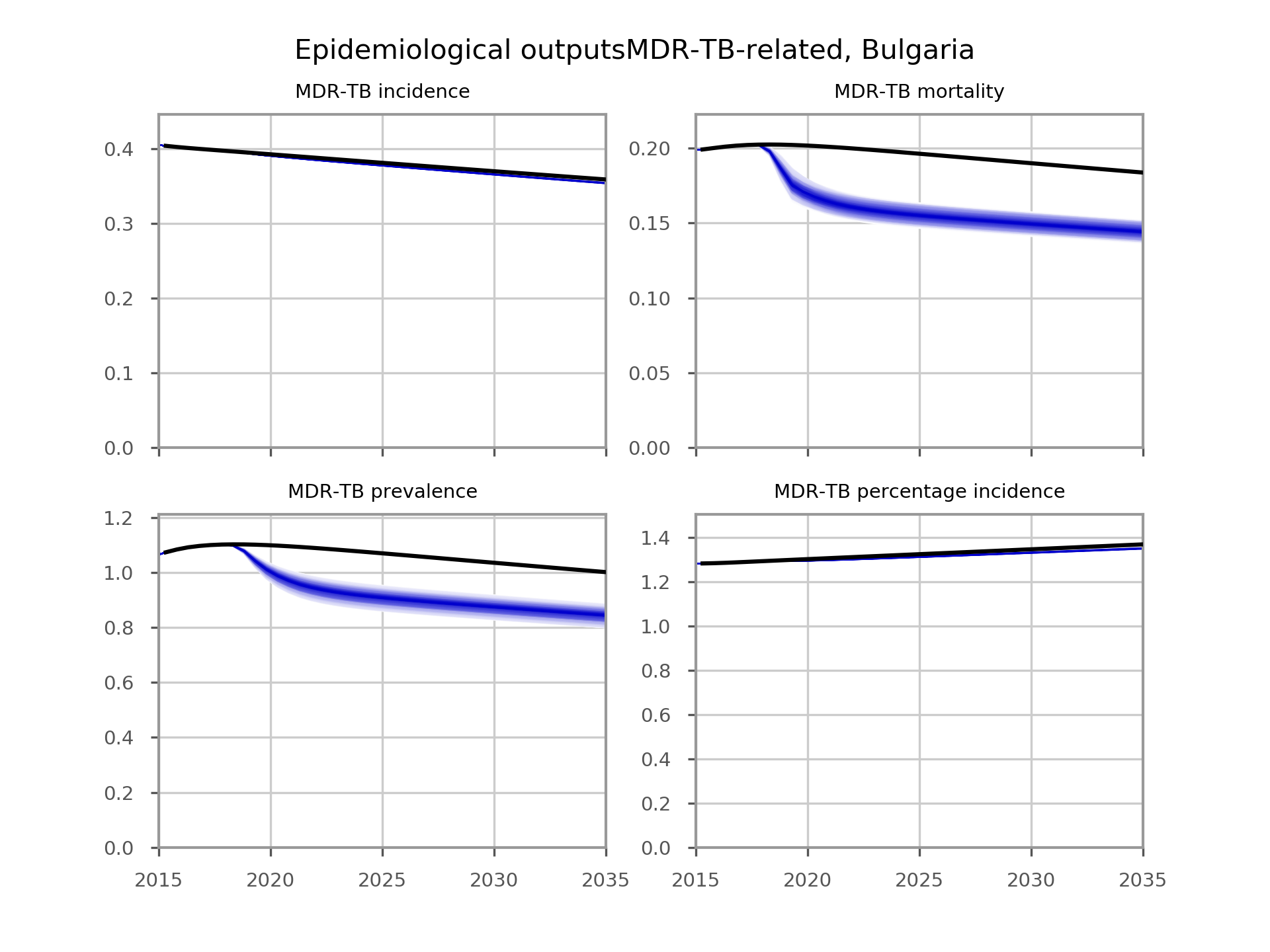


#### **Figure S29. Effect of MDR-TB short course regimen on MDR-TB epidemiological outcomes, with uncertainty.**

### Costs

The current regimen for MDR-TB is for 20 months, during which patients stay in hospital for the first 12 months, followed by eight months of ambulatory care. Under the short course regimen approach currently being considered in Bulgaria, patients will stay in hospital for the first six months and in ambulatory care for the remaining six months, with frequent home visits by nurses and NGO outreach workers. The unit costs per patient for both the 20-month regimen and 12-month regimen are calculated as follows.

#### **Table S2. Calculations of unit costs of conventional and short-course MDR-TB regimens.**

| Item | € cost per item (source) | Quantity | Total | Remarks |
| --- | --- | --- | --- | --- |
| ***20-month regimen*** | | | | |
| Screening and baseline laboratory investigations | | | | |
| DSSM | 3.06 (NTP) | 2 | 6.12 | Done twice (screening and baseline) |
| TB culture | 6.09 (NTP) | 1 | 6.09 | Done once per patient for diagnosis |
| DST | 67.12 (NTP) | 1 | 67.12 | Done once per patient for diagnosis |
| Chest x-ray | 7.76 (NTP) | 1 | 7.76 | Done once per patient for baseline |
| During treatment | | | | |
| TB culture | 6.09 (NTP) | 12 | 73.08 | Done every month for the first 8 months and quarterly thereafter |
| Bed + personnel + tests other than culture | 23 per day (NTP) | 245 days | 5 635 |  |
| Drug cost | 1046 | 1 | 1 046 | Philippines data, which are based on Global Drug Facility Product Catalogue |
| Food voucher | 1.53 per week (NTP) | 560 days | 857 | Only given to weeks of complete treatment |
| Ambulatory care | 5.01 per visit (NTP) | 32 visits | 163.2 | Weekly visit for 32 weeks of ambulatory care |
| **Total per patient** | | | | ***EUR 7 861*** |
|  | | | | |
| ***12-month regimen*** | | | | |
| Screening and baseline lab | | | | |
| DSSM | 3.06 (NTP) | 2 | 6.12 | Done twice (screening and baseline) |
| TB culture | 6.09 (NTP) | 1 | 6.09 | Done once per patient for diagnosis |
| DST | 67.12 (NTP) | 1 | 67.12 | Done once per patient for diagnosis |
| Chest x-ray | 7.76 (NTP) | 1 | 7.76 | Done once per patient for baseline |
| During treatment | | | | |
| TB culture | 6.09 (NTP) | 8 | 48.72 | Done every month for the first 6 months and quarterly thereafter |
| Bed + personnel + tests other than culture | 23 per day (NTP) | 180 days | 4 140 |  |
| Drug cost | 1137 | 1 | 1 137 | Philippines data, which are based on Global Drug Facility Product Catalogue |
| Food voucher | 1.53 per day (NTP) | 365 days | 558.45 | Only given to weeks of complete treatment |
| Ambulatory care | 5.01 per visit (NTP) | 24 visits | 122.4 | Weekly visit for 32 weeks of ambulatory care |
| **Total per patient** | | | | ***EUR 6 094*** |

#### Abbreviations: DSSM, direct sputum smear microscopy; DST, drug susceptibility testing; NTP, National TB Program.

Changing from the conventional regimen to the short-course regimen may involve initial (start-up) costs such as costs of training. Data on these costs remain lacking for Bulgaria and so are assumed to be zero.


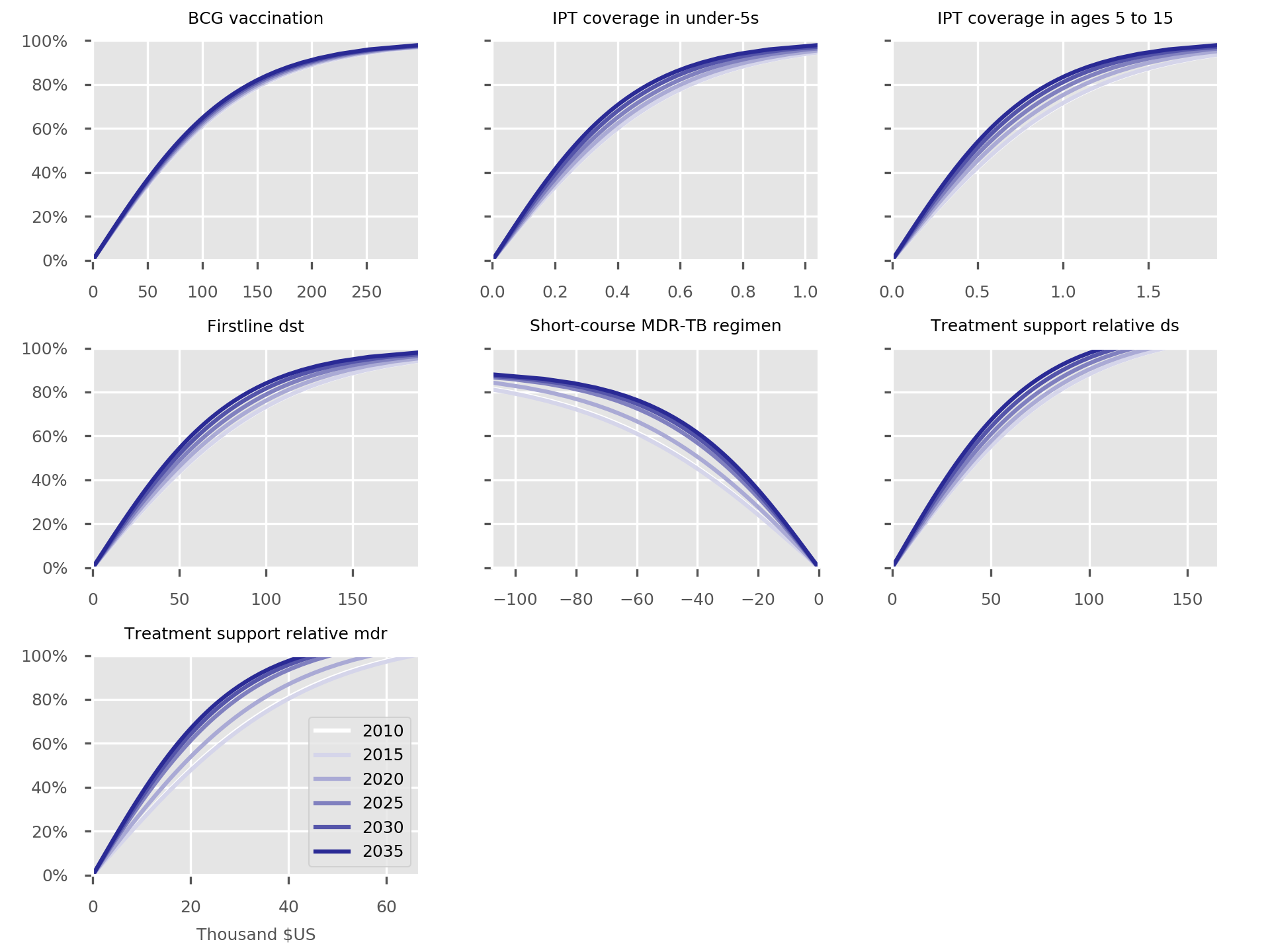


#### **Figure S30. Short course MDR-TB regimen cost-coverage curves.** Note the intentionally negative values, given this is a cost-saving intervention.

# Scale-up DST coverage

### Background

Only four laboratories (three Regional Reference Laboratories and the National Reference Laboratory) perform drug susceptibility testing (DST) for first-line drugs (FLDs). All MDR-TB strains detected in Bulgaria are referred to the National Reference Laboratory for confirmation. In 2015, FLD DST was performed on 87% of the new pulmonary TB cases with positive culture (and 85% of the retreatment cases with positive culture), despite the recommendation that DST should be performed for all culture-positive TB cases. Currently, rapid molecular tests to detect antibiotic resistance are mainly used for confirmation of MDR-TB after obtaining DST results. Despite the existence of an algorithm (indications) for the use of the molecular tests on ‘new patients suspected for MDR-TB’ and ‘previously treated patients’, these tests are rarely performed (before detection of MDR-TB by DST).

### Evidence

Phenotypic DST is considered the gold-standard for the correct identification of MDR-TB. Patients who are correctly identified as having MDR-TB should be started on a regimen that is directed at this strain of TB, rather than receiving an inappropriate regimen.

### Implementation

In the absence of DST, a baseline proportion of patients is assumed to be correctly diagnosed with MDR-TB, which is set at 25%. This is intended to represent a minority of cases for whom MDR-TB would be strongly suspected in the absence of any laboratory evidence, such as contact with a patient with MDR-TB or a patient who has received extensive past treatment. If phenotypic DST is available, the proportion correctly diagnosed is increased from this baseline value according to the follow formula:

$$proportion correctly identified MDR=proportion clinically diagnosed+\left( 1-proportion clinically diagnosed \right)\times proportion of patients for whom cultures can be obtained\times coverage of DST intervention$$

This calculation is performed separately for each organ status, as the “proportion of patients for whom cultures can be obtained” is considered to differ by organ status, taking a value of one for smear-positive patients, a value of zero for extrapulmonary patients and being an intervention uncertainty parameter for smear-negative patients (with median value 50%).

The proportion can be further increased by molecular diagnostics, although this consideration is not applicable to this application.

### Results

The effect of this intervention is minimal due to the effect primarily being on MDR-TB, which is a relatively small proportion of total burden and the fact that DST was already at a high coverage in Bulgaria before the intervention was implemented.


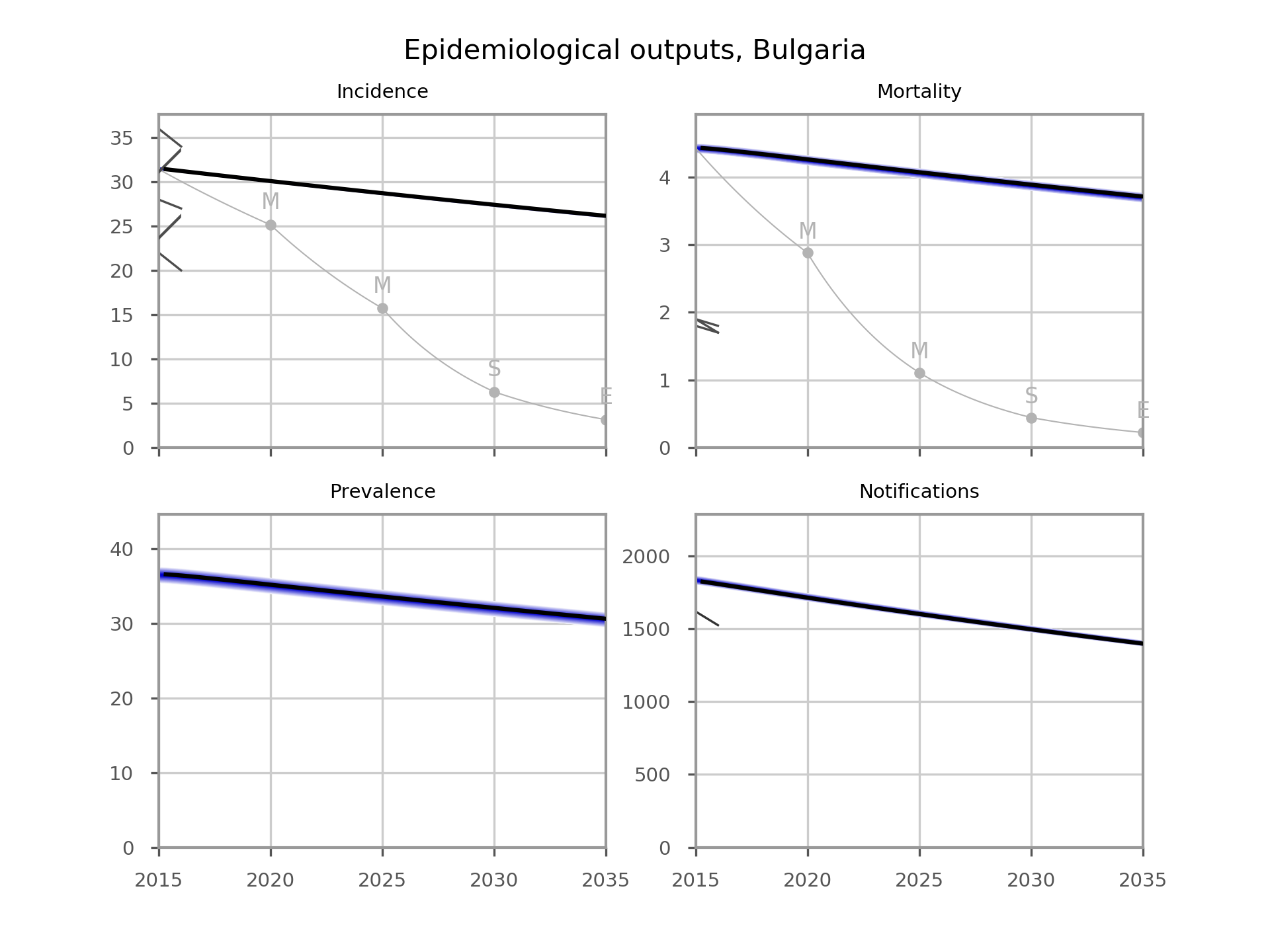


#### **Figure S31. Effect of increased DST coverage on epidemiological outcomes.**


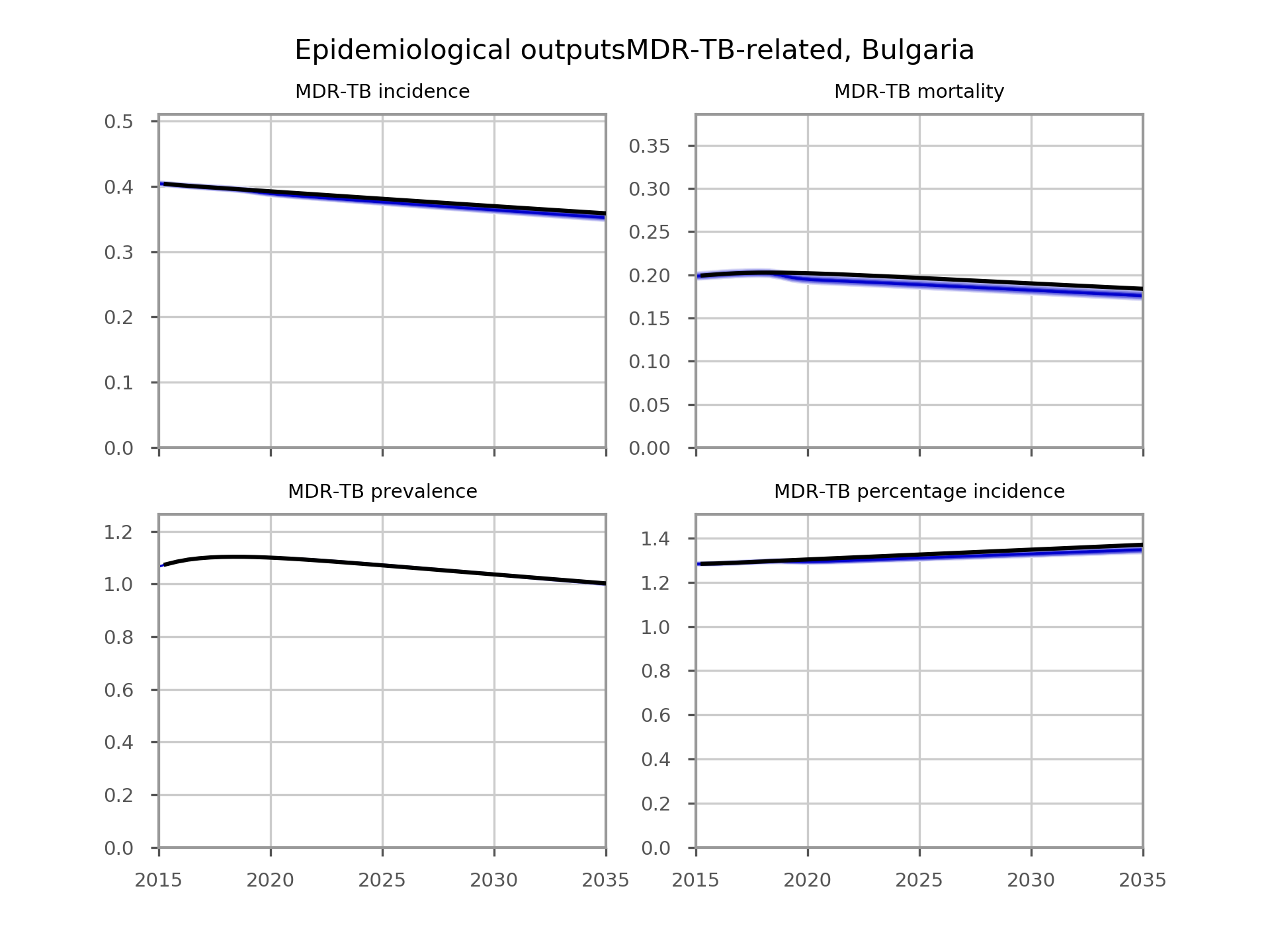


#### **Figure S32. Effect of increased DST coverage on MDR-TB epidemiological outcomes.**

### Costs

Currently, it is estimated that less than 80% of culture-positive TB cases have drug susceptibility testing (DST) performed. To increase DST and improve the detection of MDR-TB, it is planned to transport all the specimens that are not currently done DST to the National TB Reference Laboratory in Sofia. Staff at the National Reference Laboratory will receive extra payment for the additional workload.

According to the NTP’s data, the cost of transportation of one culture to the NRL is around EUR 1.02 and cost of one DST is EUR 67.12. Assuming only one DST is done for each culture-positive TB patient, the unit cost per patient is calculated as:

*Culture transportation + DST = 1.02 + 67.12 =* ***EUR 68.14***

This activity also involves hiring three more staff for the NRL, with a monthly salary of EUR 223 each. According to NTP data, in 2015 there were 782 culture-positive TB cases; 80% (626) of which have DST performed, leaving 156 cultures not having DST done. The aim of the program is to have these remaining culture-positive cases tested for drug susceptibility. It would be unrealistic to assume that the three additional staff employed at the NRL would only undertake DST, given an estimated total of 156 additional cultures per year. Therefore, we determined that this cost should be accounted for as a start-up cost for one year for the NRL to get up to speed with the additional workload. That is, the start-up cost of the program is assumed to be: *3 × 223 × 12 =* ***EUR*** ***8 028.***

# Scale-up food vouchers to reach all patients under treatment

### Background

Although evidence is variable and past research has been inconsistent, in discussions with the country team, it was felt likely that interventions (such as food vouchers) to support patients on treatment are effective in improving patient adherence and treatment outcomes in the Bulgarian context. This intervention has been fully in place for MDR-TB patients and as covered 50% of all DS-TB patients since 2010. It is considered an important component of TB control in the country, although its epidemiological impact has not been evaluated. Here we consider the extension of this program all patients under treatment, to include those with drug-susceptible TB.

### Evidence

A study from Timor Leste found no effect on overall treatment outcomes, but improved weight gain and improvements for some patient sub-groups.^37^ In a randomised controlled trial undertaken in South Africa, economic support to patients with TB did not significantly improve treatment outcomes, although many of the patients randomised to support did not receive the intervention and outcomes improved among the subgroup who did receive support.^38^ In a cohort study in a country nearby to Bulgaria (Moldova), incentives were found to be effective at improving treatment outcomes, with a reduction in unfavourable outcomes by around 50%.^39^

### Implementation

For model implementation, we incorporate a proportional reduction in unfavourable treatment outcomes under which the proportion of treatment outcomes resulting in unfavourable outcomes are reduced to 0.5 (multiplied by the coverage of the intervention) times the pre-intervention values, consistent with findings from Moldova.

Because this intervention is considered to have had historical coverage prior to the commencement of the intervention period, it was necessary to calculate a theoretical treatment success rate to represent the success rate that would have been present if the intervention had been absent. Once this has been done, scaling up the future coverage from its baseline value (of 50% for DS-TB patients) can be simulated.

### Results

As this intervention directly reduces deaths on treatment, this intervention has its main effect on TB-related mortality. Due to the high background case detection rate in Bulgaria, a large proportion of TB-related deaths accrue in patients currently under treatment, such that changes in the proportion of treatment outcomes resulting in death have a significant effect. By contrast, the effects on overall disease burden are indirect and occur through a reduction in the number of infectious persons, which results from few patients transitioning back to the infectious compartment following non-death unsuccessful treatment outcomes. Therefore, the reductions in incidence, prevalence and notifications are small. Significant reductions in the burden of MDR-TB are also noted, in spite of this intervention being directed at DS-TB. This is mediated through a reduction in non-success non-death treatment outcomes for DS-TB reducing the rate of amplification of previously DS strains to MDR-TB.


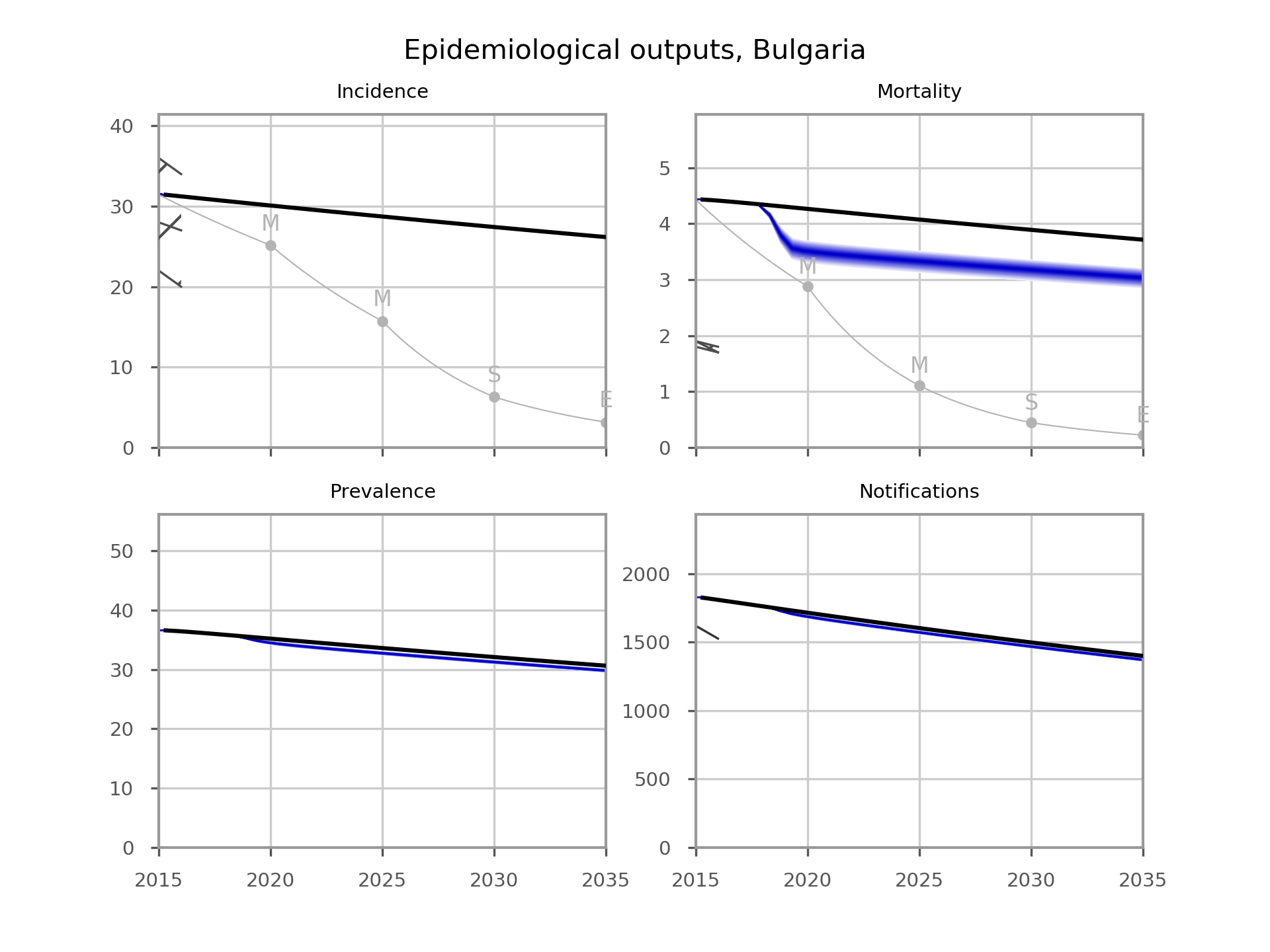


#### **Figure S33. Effect of food vouchers on epidemiological outcomes, with uncertainty.**


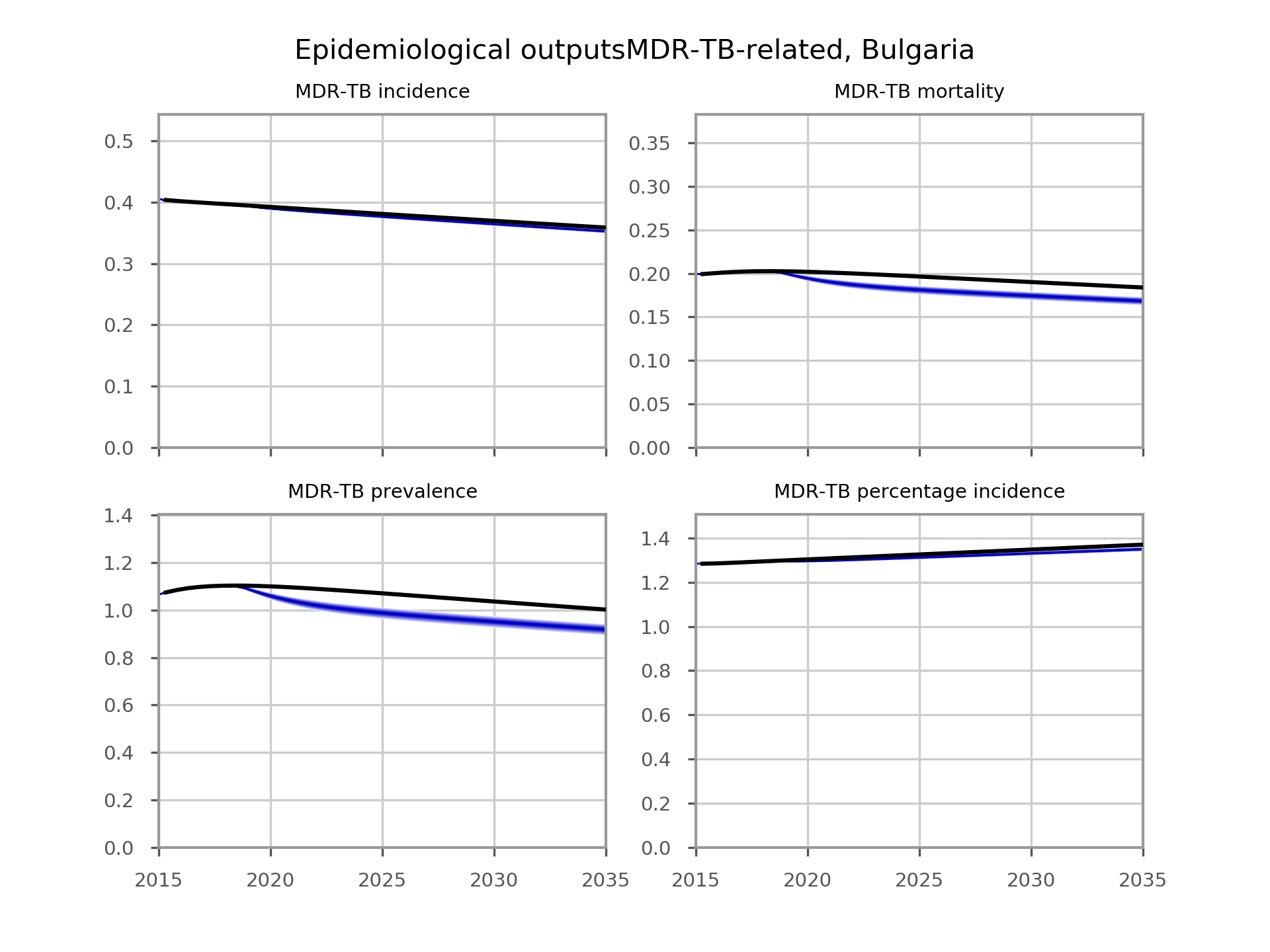


#### **Figure S34. Effect of food vouchers on MDR-TB epidemiological outcomes, with uncertainty.**

### Costs

DS-TB patients receive on average one food voucher valued at EUR 5.1 for every week of complete treatment during the outpatient continuation phase, which is 18 weeks on average. Therefore, the unit cost per person is calculated as **18 × 5.1 = €92**.

As this is an existing intervention which commenced in 2010 and expansion of the program only involves providing food vouchers to more patients, the start-up costs are assumed to be zero. The saturation value for the cost coverage curves is set at 110%, because a ceiling at 100% would lead to extremely high and unrealistic costs as coverage approaches the horizontal asymptote at 100% coverage. As seen from Figure S35, the effect of setting saturation to 110% is to achieve a small but reasonable increase in unit costs as coverage reaches the target value of 100%.

In the case of MDR-TB, patients receive EUR 1.53 per day for the duration of treatment (average 560 days), such that the cost per regimen is calculated as 560 × 1.53 = €857. However, note that the coverage of this program is 100% at baseline and not modified by the intervention.


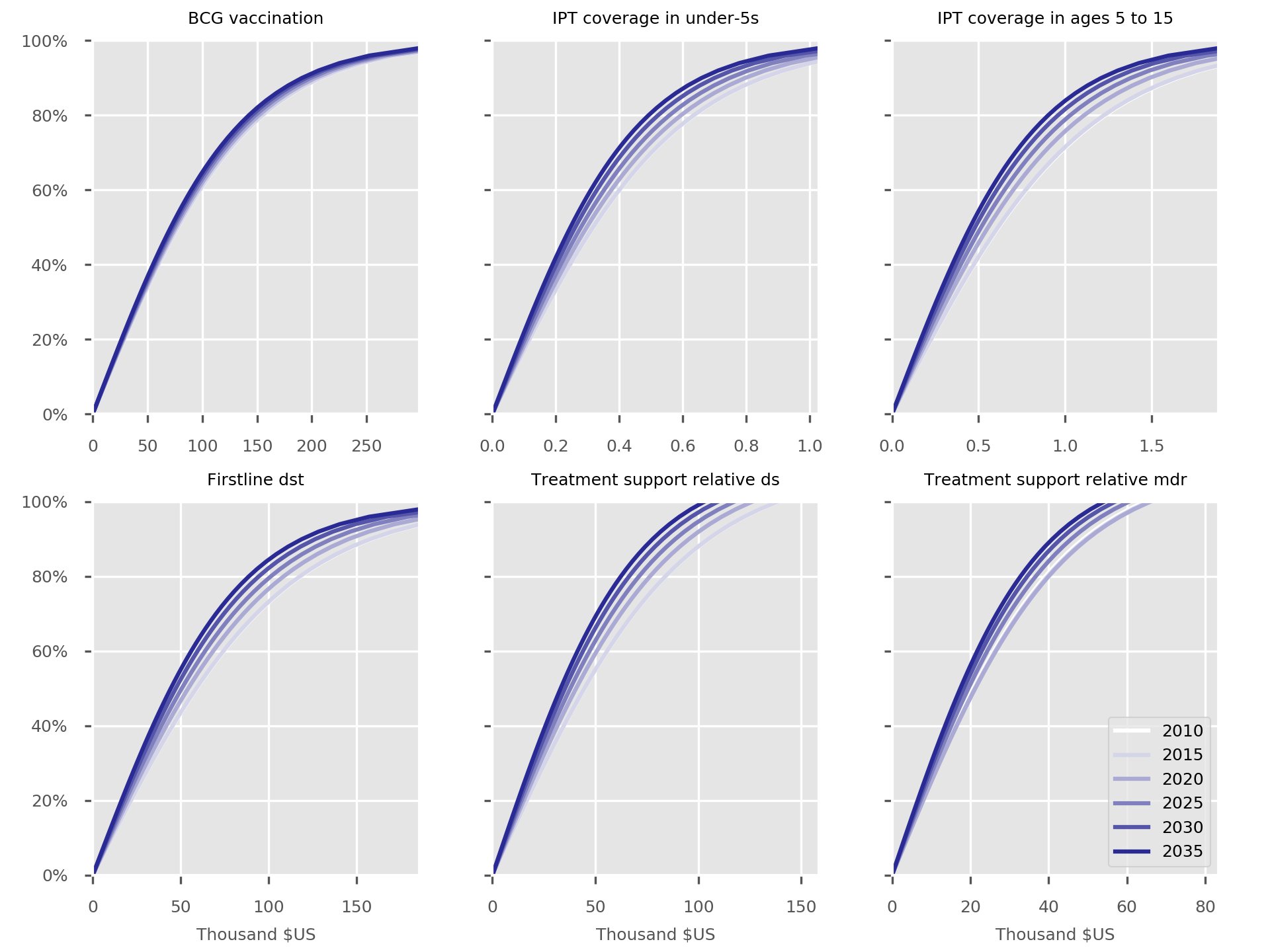


#### **Figure S35. Cost-coverage curves for food voucher intervention.**

# Ambulatory care for smear-negative and extrapulmonary DS-TB

### Background

Ambulatory care refers to the treatment and care of patients outside of hospitals. Worldwide, drug-resistant forms of TB are often managed for extended periods of time as inpatients, although drug-susceptible TB is typically managed primarily in an outpatient setting. Bulgaria has a history of providing TB care on a primarily inpatient basis. This intervention applies to smear-negative and extrapulmonary DS-TB patients only, while management of smear-positive TB remains primarily inpatient-based. Currently, patients with smear-negative and extrapulmonary DS-TB are treated in an inpatient setting for the first three months of treatment, followed by three months (for smear-negative) or six months (for extrapulmonary) continuation ambulatory care at home. The NTP is planning to transition to a more sustainable model of care, in which these patients will receive ambulatory care for the entire course of treatment (i.e. six months ambulatory care for smear-negative DS-TB and nine months ambulatory care for extrapulmonary DS-TB). In this model of care, the patients take medicines at home with daily (first three months of treatment) or weekly (the remaining duration of treatment) visits from nurses or non-governmental organisation (NGO) outreach workers to the patient’s home. This intervention may be considered as a first step in a broader transition towards ambulatory care for all forms of DS-TB including smear-positive TB.

### Evidence

A large systematic review and meta-analysis which included data from 35 studies that compared treatment outcomes of MDR-TB patients in hospital-based and ambulatory-based settings found that both settings had comparable treatment success rates and adverse outcome rates.^40^ These findings are consistent with another systematic review, which found that ambulatory care of MDR-TB patients resulted in comparable treatment outcomes to hospital-based care, but was cheaper and so more cost-effective.^41^ A qualitative analysis of patients’ experience with ambulatory care has revealed that this modality of care was preferable to hospital-based care and was perceived as safe, conducive to recovery, facilitated psychological support and allowed more free time and earning potential for patients and caretakers.^42^ The efficacy of ambulatory management and treatment courses incorporating at least five drugs lasting for over 18 months for both MDR-TB and XDR-TB patients was recently confirmed in another systematic review and meta-analysis.^43^

### Implementation

No change to programmatic parameters is considered with this implementation, although it is conceivable that improvements in treatment success rates could be observed based on the evidence described above. The cost-coverage curves for the additional positive costs of moving to an ambulatory approach to care for smear-negative and extrapulmonary patients is displayed in Figure S36.

### Costs

Information on the activities required (such as training) for the transition from hospitalisation care to ambulatory care was unavailable. Therefore, the start-up cost is assumed to be zero. The other costs are estimated as follows.

#### **Table S3. Calculations of unit costs of conventional and ambulatory modality of care.**

| Item | | € cost per item (source) | | Quantity | | Total | | Remarks |
| --- | --- | --- | --- | --- | --- | --- | --- | --- |
| ***Conventional modality of care*** | | | | | | | | |
| Hospital bed + personnel + tests other than *Mtb* tests | | 15.34 per day (NTP) | | 90 days | | 1380 | |  |
| Ambulatory care | | 5.01 per visit (NTP) | | 12 (smear-negative | | 60.12 | | Weekly visit by NGO for 12 weeks of ambulatory care |
|  |  |  |  | 24 (extrapulmonary) | | *120.24* | | Weekly visit by NGO for 24 weeks of ambulatory care |
| Drug cost | | 86 (smear-negative) | | 1 | | 86 | | ^44^ |
|  |  | 129 (smear-positive) | | 1 | | *129* | | Assume 1.5 times higher than smear-negative because duration of treatment is 1.5 times longer. |
| TST | | 3.57 (NTP) | | 1 | | 3.57 | |  |
| X-ray | | 7.76 (NTP) | | 2 | | 15.52 | |  |
| DSSM | | 3.06 (NTP) | | 4 | | 12.24 | |  |
| Culture | | 6.09 (NTP) | | 3 | | 18.27 | |  |
| DST | | 67.14 (NTP) | | 1 | | 67.14 | |  |
| **Total per patient** | | | | | | | | ***EUR 1 643 (smear-negative)***  ***EUR 1 746 (extrapulmonary)*** |
|  | | | | | | | | |
| ***Ambulatory care*** | | | | | | | | |
| Hospital bed + personnel + tests other than Mtb tests |  | |  | | 0 | |  | |
| NGO visit to patient’s home | 5.01 per visit (NTP) | | 102 (smear-negative | | 511 | | Daily for the first three months then weekly visit by NGO for the remaining three months | |
|  |  |  | 114 (extrapulmonary) | | *571* | | Daily for the first three months then weekly visit by NGO for the remaining six months | |
| Drug cost | 86 (smear-negative) | | 1 | | 86 | | *Laurence et al. PharmacoEconomics 2015. 33: 939-955* | |
|  | 129 (smear-positive) | | 1 | | *129* | | Assume 1.5 times higher than smear-negative because duration of treatment is 1.5 times longer | |
| TST | 3.57 (NTP) | | 1 | | 3.57 | |  | |
| Chest x-ray | 7.76 (NTP) | | 2 | | 15.52 | |  | |
| DSSM | 3.06 (NTP) | | 4 | | 12.24 | |  | |
| Culture | 6.09 (NTP) | | 3 | | 18.27 | |  | |
| DST | 67.14 (NTP) | | 1 | | 67.14 | |  | |
| Patient visit to clinic | 2.5 | | 15 (smear-negative) | | 37.5 | | weekly for the first 3 months then monthly | |
|  |  |  | 18 (extrapulmonar) | | 45 | |  |  |
| **Total per patient** | | | | | | | ***EUR 751 (smear-negative)***  ***EUR 862 (extrapulmonary)*** | |

#### Abbreviations: DSSM, direct sputum smear microscopy; DST, drug susceptibility testing; NGO, Non-governmental organisation; NTP, National TB Program; TST, tuberculin skin test.


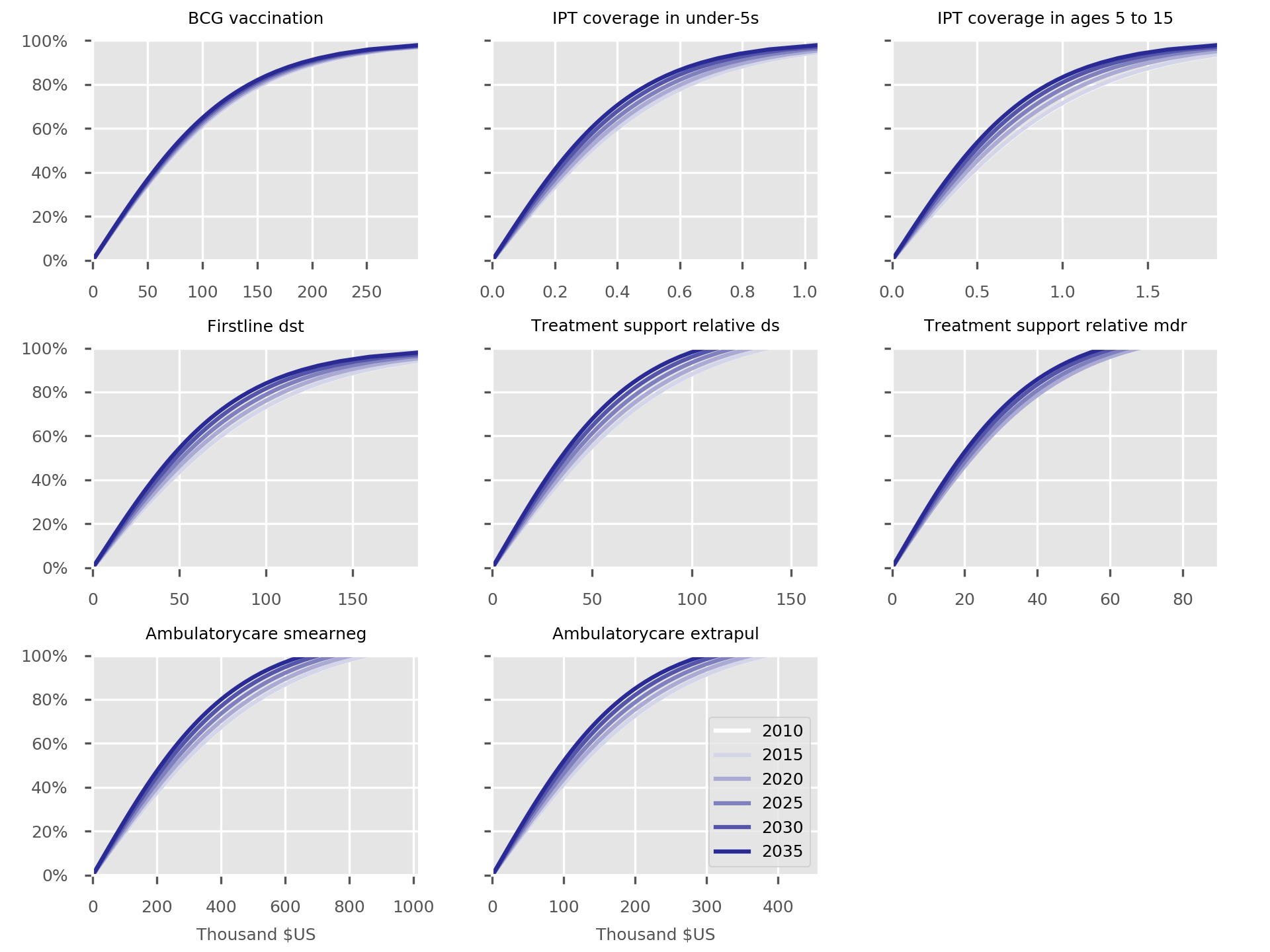


#### **Figure S36. Cost-coverage curves for ambulatory care intervention.**

# Discontinue Open Doors

### Background

The “Open Doors” program has been an important contributor to Bulgaria’s historical rates of case detection. From 2009 hospitals across the country have regularly opened their doors to the public who believe they might have respiratory symptoms. Those who are considered by the treating doctor to have symptoms suggestive of TB undergo further diagnostic tests and clinical examination.

### Evidence

This program contributes significantly to the identification and detection of TB cases in the country. For example in 2015, a total of 1,487 people were consulted and examined by a doctor after presenting to the hospital as part of the program. Of these, 27 active TB cases and 99 LTBI cases were diagnosed. As a result of the completion of the current Global Fund grant at the end of 2018, the Open Doors program will likely be discontinued if no alternative source of funding can be found, potentially leading to a worsening of case detection.

### Implementation

Withdrawal of this program results in a proportional reduction of the case detection ratio which is equal to the product of the proportion of case detection contributed by the Open Doors program and the coverage of the program:

$$new case detection ratio=baseline case detection ratio-NGOs' contribution to case detection \times\left( 1-intervention coverage \right)$$

Where the coverage of the intervention is allowed to fall below one as the intervention is withdrawn and “NGOs’ contribution to case detection” is the proportion of all notifications contributed by NGOs in Bulgaria.

As for the food voucher intervention, because this intervention is considered to have had historical coverage prior to the commencement of the intervention period, it was necessary to calculate a theoretical case detection rate representing the value that would have been present if the intervention had been absent. Once this has been done, scaling up the future coverage from its baseline value can be simulated.

### Results

The effect of withdrawal of the Open Doors program is to lead to a significant increase in TB-related mortality and prevalence. Due to the decrease in case detection, the number of notifications reported is anticipated to fall. The impact on incidence is less, due to low levels of *Mtb* transmission simulated. The proportionate effect on MDR-TB is less than the effect on all forms of TB. Costs were not estimated for this intervention.


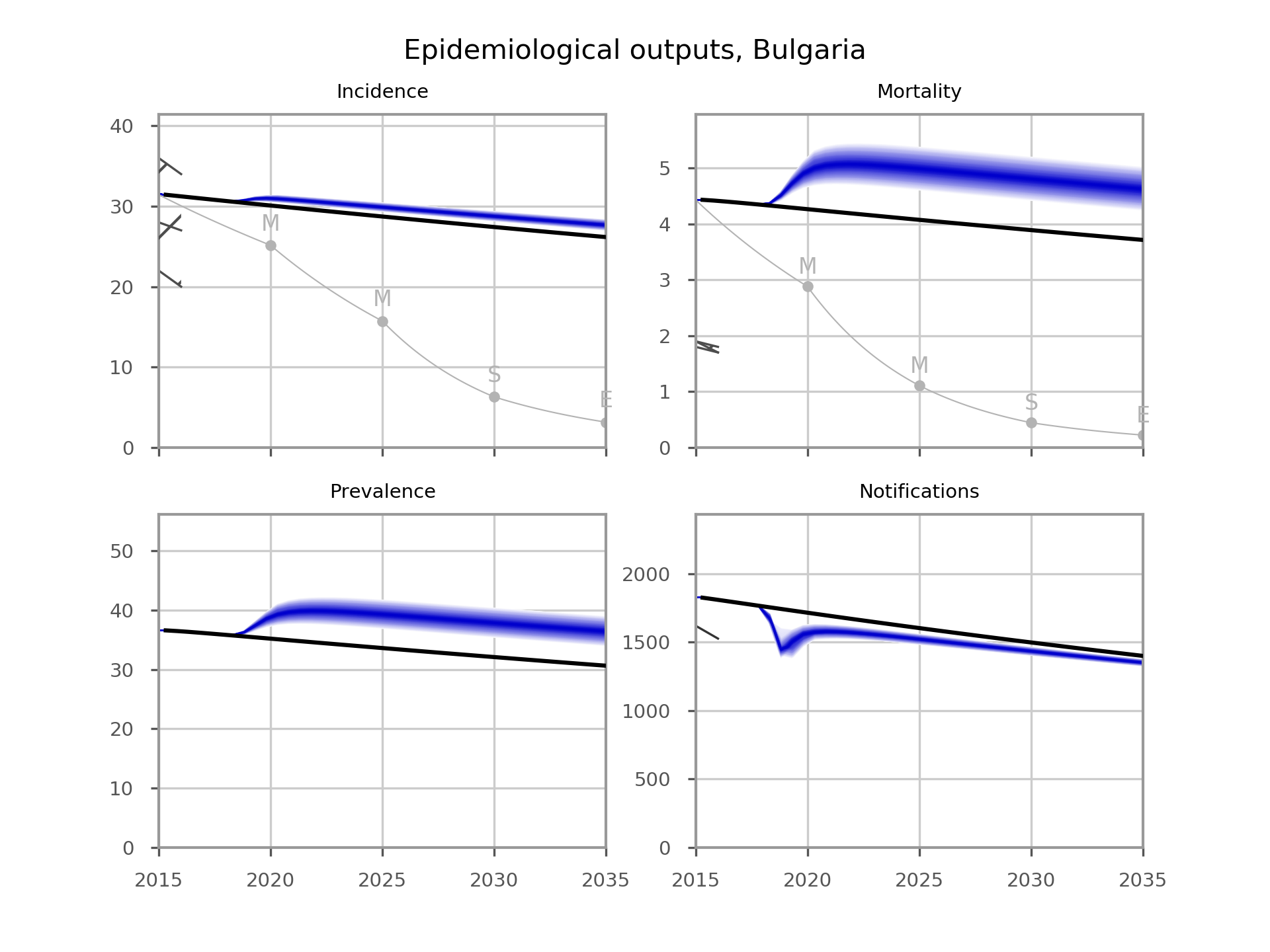


#### **Figure S37. Effect of “Open Doors” on epidemiological outcomes.**


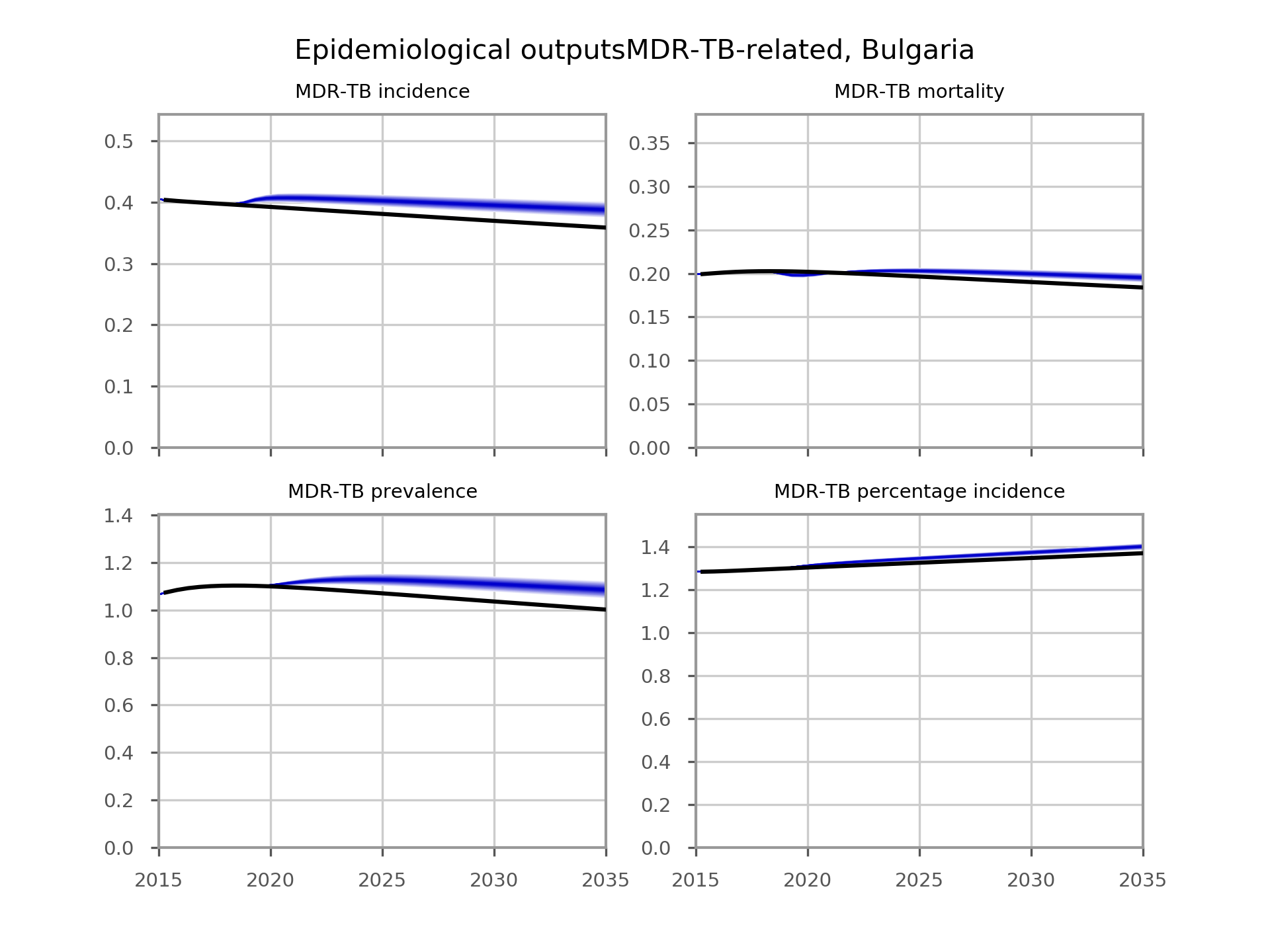


#### **Figure S38. Effect of “Open Doors” on MDR-TB epidemiological outcomes.**

# Discontinue NGO activities

### Background

Active since 2009, NGOs have been playing an essential role in TB control in Bulgaria. Their TB care activities range from mapping of risk groups, awareness raising, coordinating with local hospitals, field activities, primary TB screening, and providing support to patients under treatment, such as home visits to ensure appropriate use of TB medications of patients and accompanying patients to TB clinic when needed. These interventions are predominantly undertaken to support the Roma community.

### Evidence

To date, published data on the epidemiological impact of NGO activities on TB epidemic are lacking. Empirical data from the Bulgaria NTP suggest that nationwide in 2016, 1,603 active TB cases were identified, around 30% of them coming from the Roma community (i.e. around 481 Roma TB patients identified in total). NTP data suggest that in 2016, 76 cases of DS-TB in the Roma community were identified by NGOs. Therefore, we assume that NGOs identified 16% of all Roma TB cases (76/481). In 2016, 2,983 persons with LTBI were identified in Bulgaria. For equivalence to the proportion of active TB, we assume that the Roma community represents 30% of the national LTBI burden. That is, around 895 Roma persons with LTBI were identified in Bulgaria in 2016. NTP data suggest that in 2016, 380 persons with LTBI were identified among Roma community by NGOs. Therefore we assume that NGOs were responsible for detecting 42% of all Roma LTBI patients identified (380/895). In addition to identifying TB cases, involvement in TB care and providing support to patients during treatment are other important roles of NGOs. It is estimated that without the engagement of NGOs in TB care, the treatment success rate would have been reduced by 26%.

### Implementation

Under this intervention, the Open Doors program ceases, with all the effects described in the previous section also occurring under this scenario. In addition, case detection for the Roma community falls, with the calculation of the new case detection ratio being identical to that described in the previous section, but only applied to the Roma population.

### Results

The results of this scenario are similar to those for discontinuation of the Open Doors program, except that the effect on the Roma community is modestly greater (see Figure 3 of main manuscript).


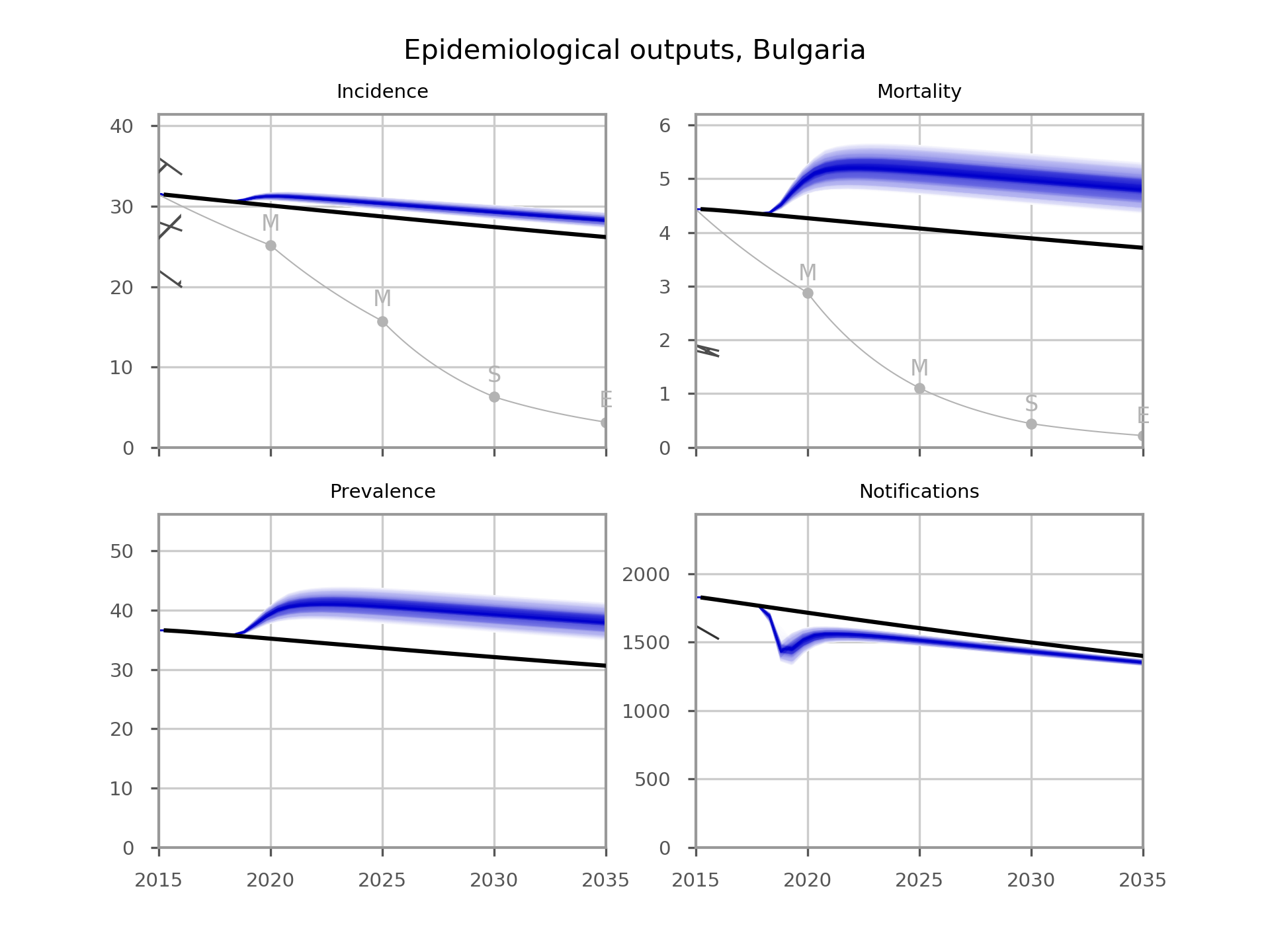


#### **Figure S39. Effects of NGO activities on TB epidemic, with uncertainty.**


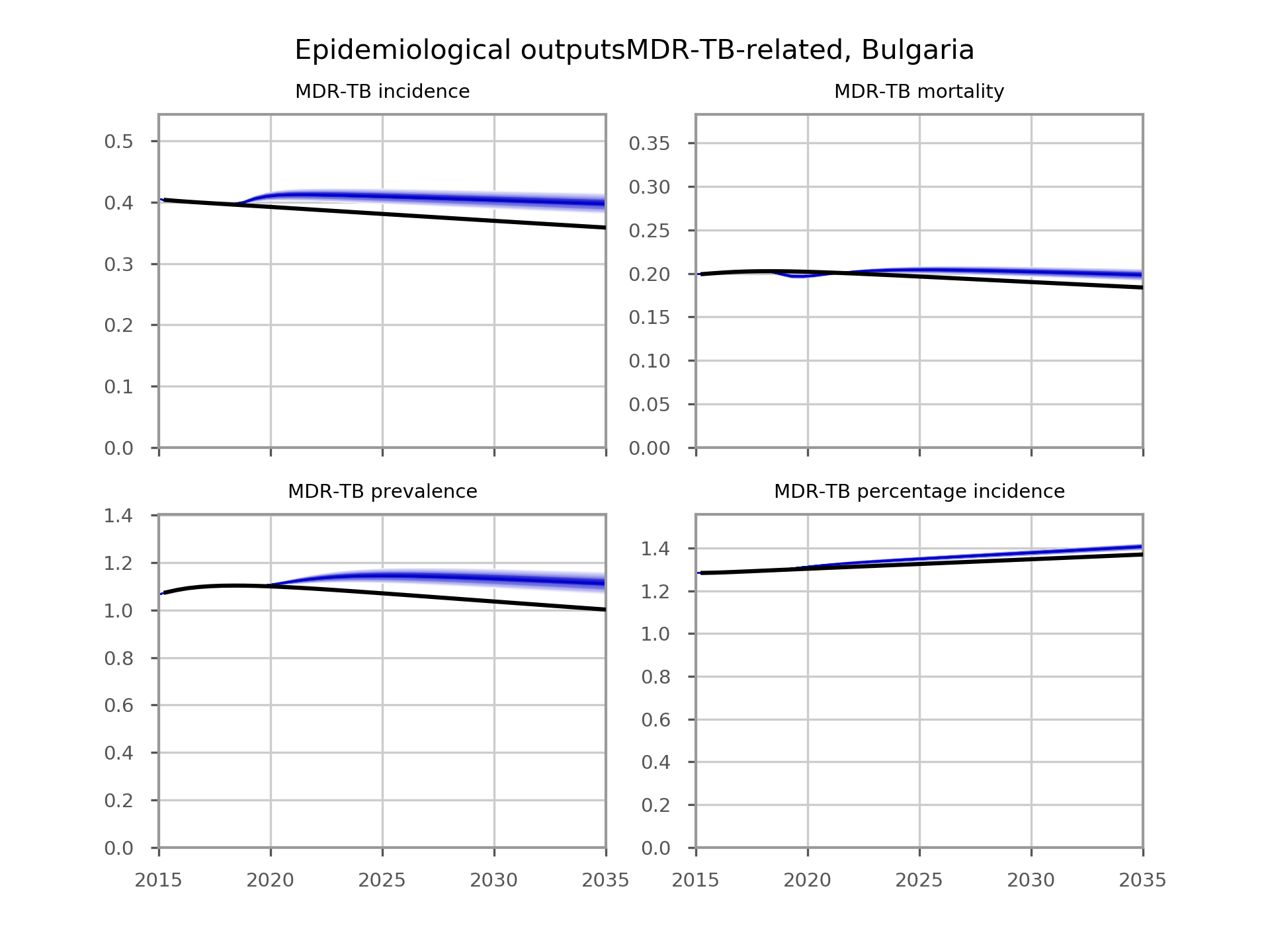


#### **Figure S40. Effects of NGO activities on MDR-TB epidemic, with uncertainty.**

# Intervention uncertainty parameters

The following Table summarises the uncertainty ranges for the parameter values relevant to specific interventions that are varied in intervention uncertainty. This is achieved with a Latin Hypercube sampling approach, whereby 50 samples are taken without replacement from equal divisions of the range of each parameter space listed below.

**Table S4. Uncertainty ranges for parameter values**

| Intervention | Parameters varied | Baseline parameter value and uncertainty range |
| --- | --- | --- |
| 1. Short course MDR-TB regimen | Treatment success proportion | 89 (80 to 95) % |
|  | Treatment duration | 11 (9 to 15) months |
| 2. Scale-up DST coverage | Proportion of smear-positive patients for whom cultures can be obtained | 95 (90 to 100) % |
|  | Proportion of smear-negative patients for whom cultures can be obtained | 50 (35 to 70) % |
| 3. Scale-up food vouchers to reach all patients under treatment | Proportional reduction in unsuccessful treatment outcomes | 43 (40 to 60) % |
| 4. Implement ambulatory care for smear-negative and extrapulmonary DS-TB | Not applicable | |
| 5. Discontinue Open Doors intervention | Proportion of all case detection contributed by the Open Doors program | 8 (5 to 12) % |
| 6. Discontinue NGO activities | Proportion of all case detection contributed by the Open Doors program | 8 (5 to 12) % |
|  | Proportion of case detection in Roma communities contributed by NGOs | 25 (15 to 35) % |

# References

1. **Trauer JM *et al*.** (2017) Modular programming for tuberculosis control, the “AuTuMN” platform. *BMC Infectious Diseases* **17,** 546.

2. **Borissova A-M *et al.*** (2015) Changes in the prevalence of diabetes mellitus in bulgaria (2006-2012). *Clinical Medicine Insights: Endocrinology and Diabetes* **8,** 41–45.

3. **The World Bank.** World development indicators. Available at: http://databank.worldbank.org/data/reports.aspx?source=world-development-indicators. (Accessed: 27th February 2017)

4. **The World Bank**. Life expectancy at birth. Available at: http://data.worldbank.org/indicator/SP.DYN.LE00.IN. (Accessed: 19th June 2017)

5. **Andrews JR *et al.*** (2012) Risk of progression to active tuberculosis following reinfection with Mycobacterium tuberculosis. *Clinical Infectious Diseases* **54,** 784–791.

6. **Verver S *et al.*** (2005) Rate of reinfection tuberculosis after successful treatment is higher than rate of new tuberculosis. *American Journal of Respiratory and Critical Care Medicine* **171,** 1430–1435.

7. **Gomes MGM *et al.*** (2012) How host heterogeneity governs tuberculosis reinfection? *Proceedings of the Royal Society B: Biological Sciences* **279,** 2473–2478.

8. **Trauer JM *et al.*** (2016) Risk of active tuberculosis in the five years following infection ⋯ 15%? *Chest* **149,** 516-525.

9. **Sloot R *et al*.** (2014) Risk of Tuberculosis after Recent Exposure. A 10-Year Follow-up Study of Contacts in Amsterdam. *American Journal of Respiratory and Critical Care Medicine* **190,** 1044–1052.

10. **Ragonnet R *et al.*** Optimally capturing latency dynamics in models of tuberculosis transmission. *Epidemics* **21,** 39–47.

11. **Dowdy DW, Dye C and Cohen T.** (2013) Data Needs for Evidence-Based Decisions: a Tuberculosis Modeler’s ‘Wish List’. *International Journal of Tuberculosis and Lung Disease* **17,** 866–877.

12. **Menzies NA *et al.*** (2018) Progression from latent infection to active disease in dynamic tuberculosis transmission models: a systematic review of the validity of modelling assumptions. *Lancet Infectious Diseases* **18**:e228-e238.

13. **Tiemersma EW et al.** (2011) Natural history of tuberculosis: duration and fatality of untreated pulmonary tuberculosis in HIV negative patients: a systematic review. *PLoS One* **6,** e17601.

14. **World Health Organization.** Global Tuberculosis Report 2016.

15. **Marais BJ *et al.*** (2004) The natural history of childhood intra-thoracic tuberculosis: a critical review of literature from the pre-chemotherapy era. *International Journal of Tuberculosis and Lung Disease* **8,** 392–402.

16. **Cruz AT and Starke JR.** (2007) Clinical manifestations of tuberculosis in children. *Paediatric Respiratory Reviews* **8,** 107–117.

17. **Matlow A, Robb M and Goldman C.** (2003) Infection control and paediatric tuberculosis: A practical guide for the practicing paediatrician. *Paediatrics & Child Health* **8,** 624–6.

18. **Starke JR.** (2001) Transmission of mycobacterium tuberculosis to and from children and adolescents. *Seminars in Pediatric Infectious Diseases* **12,** 115–123.

19. **Hussey G, Chisholm T and Kibel M.** (1991) Miliary tuberculosis in children: a review of 94 cases. *The Pediatric Infectious Disease Journal* **10,** 832–836.

20. **Straetemans M *et al*.** (2011) Assessing tuberculosis case fatality ratio: a meta-analysis. *PLoS One* **6,** e20755.

21. **Pablos-Méndez A, Blustein J and Knirsch CA.** (1997) The role of diabetes mellitus in the higher prevalence of tuberculosis among Hispanics. *American Journal of Public Health* **87,** 574–579.

22. **Alisjahbana B *et al.*** (2006) Diabetes mellitus is strongly associated with tuberculosis in Indonesia. *International Journal of Tuberculosis and Lung Disease* **10,** 696–700.

23. **Kim SJ *et al*.** (1995) Incidence of pulmonary tuberculosis among diabetics. *Tubercle and Lung Disease***76,** 529–533.

24. **Ponce-De-Leon A *et al.*** (2004) Tuberculosis and diabetes in southern Mexico. *Diabetes Care* **27,** 1584–1590.

25. **Pérez A *et al***. (2006) Association between tuberculosis and diabetes in the Mexican border and non-border regions of Texas. *American Journal of Tropical Medicine and Hygiene* **74,** 604–611.

26. **Oluboyo PO and Erasmus RT.** (1990) The significance of glucose intolerance in pulmonary tuberculosis. *Tubercle* **71,** 135–138.

27. **Başoğlu OK *et al.*** (1999) The oral glucose tolerance test in patients with respiratory infections. *Monaldi Archives for Chest Disease* **54,** 307–310.

28. **Jeon CY and Murray MB.** (2008) Diabetes mellitus increases the risk of active tuberculosis: a systematic review of 13 observational studies. *PLoS Medicine* **5,** e152.

29. **Council of Ministers, Republic of Bulgaria.** National Programme for prevention and control of tuberculosis in the republic of Bulgaria for the period 2016-2020.

30. **Trauer JM *et al.*** (2016) Modelling the effect of short-course multidrug-resistant tuberculosis treatment in Karakalpakstan, Uzbekistan. *BMC Medicine* **14,** 187.

31. **Van Deun A *et al.*** (2010) Short, highly effective, and inexpensive standardized treatment of multidrug-resistant tuberculosis. *American Journal of Respiratory and Critical Care Medicine* **182,** 684–692.

32. **Piubello A *et al.*** (2014) High cure rate with standardised short-course multidrug-resistant tuberculosis treatment in Niger: no relapses. *International Journal of Tuberculosis and Lung Disease* **18,** 1188–1194.

33. **Kuaban C *et al.*** (2015) High effectiveness of a 12-month regimen for MDR-TB patients in Cameroon. *International Journal of Tuberculosis and Lung Disease* **19,** 517–524.

34. **Aung KJ *et al.*** (2014) Successful ‘9-month Bangladesh regimen’ for multidrug-resistant tuberculosis among over 500 consecutive patients. *International Journal of Tuberculosis and Lung Disease* **18,** 1180–1187.

35. **Lange C *et al.*** (2016) Limited Benefit of the New Shorter Multidrug-Resistant Tuberculosis Regimen in Europe. *American Journal of Respiratory and Critical Care Medicine* **194,** 1029–1031.

36. **Günther G *et al.*** (2015) Multidrug-Resistant Tuberculosis in Europe, 2010–2011. *Emerging Infectious Diseases* **21,** 409–416.

37. **Martins N, Morris P and Kelly PM.** (2009) Food incentives to improve completion of tuberculosis treatment: randomised controlled trial in Dili, Timor-Leste. *BMJ* **339**, b4248

38. **Lutge E *et al*.** (2013) Economic support to improve tuberculosis treatment outcomes in South Africa: a pragmatic cluster-randomized controlled trial. *Trials* **14,** 154.

39. **Ciobanu A *et al.*** (2014) Do incentives improve tuberculosis treatment outcomes in the Republic of Moldova? *Public Health Action* **4,** 59–63.

40. **Bassili A *et al.*** (2013) A systematic review of the effectiveness of hospital- and ambulatory-based management of multidrug-resistant tuberculosis. *American Journal of Tropical Medicine and Hygiene* **89,** 271–280.

41. **Fitzpatrick C and Floyd K.** (2012) A Systematic Review of the Cost and Cost Effectiveness of Treatment for Multidrug-Resistant Tuberculosis. *Pharmacoeconomics* **30,** 63–80.

42. **Horter S *et al.*** (2014) “Home is where the patient is”: a qualitative analysis of a patient-centred model of care for multi-drug resistant tuberculosis. *BMC Health Services Research* **14,** 81.

43. **Williams AO, Makinde OA and Ojo M.** (2016) Community-based management versus traditional hospitalization in treatment of drug-resistant tuberculosis: a systematic review and meta-analysis. *Global Health Research and Policy* **1,** 10.

44. **LaurenceYV, Griffiths UK and Vassall A.** (2015) Costs to Health Services and the Patient of Treating Tuberculosis: A Systematic Literature Review. *Pharmacoeconomics* **33,** 939–955.

1. Note that we use the term “strain” throughout to refer to forms of *Mtb* that differ according to their drug susceptibility profile. Therefore, these are not necessarily strains in a phylogenetic sense. [↑](#footnote-ref-1)
